# High mtDNA content identifies oxidative phosphorylation-driven acute myeloid leukemias and represents a therapeutic vulnerability

**DOI:** 10.1038/s41392-025-02303-x

**Published:** 2025-07-14

**Authors:** Diego A. Pereira-Martins, Isabel Weinhäuser, Emmanuel Griessinger, Juan L. Coelho-Silva, Douglas R. Silveira, Dominique Sternadt, Ayşegül Erdem, Bruno Kosa L. Duarte, Prodromos Chatzikyriakou, Lynn Quek, Antonio Bruno Alves-Silva, Fabiola Traina, Sara T. Olalla Saad, Jacobien R. Hilberink, Amanda Moreira-Aguiar, Maria L. Salustiano-Bandeira, Marinus M. Lima, Pedro L. Franca-Neto, Marcos A. Bezerra, Nisha K. van der Meer, Emanuele Ammatuna, Eduardo M. Rego, Gerwin Huls, Jan Jacob Schuringa, Antonio R. Lucena-Araujo

**Affiliations:** 1 Department of Genetics, Federal University of Pernambuco, Recife, Brazil; 2 Department of Hematology, University Medical Center Groningen, University of Groningen, Groningen, the Netherlands; 3 Department of Medical Imaging, Haematology, and Oncology, Ribeirão Preto Medical School, University of São Paulo, Ribeirão Preto, SP Brazil; 4 Center for Cell Based Therapy, São Paulo Research Foundation, Ribeirão Preto, SP Brazil; 5 Myeloid Leukaemia Genomics and Biology Group, School of Cancer and Pharmaceutical Sciences, King’s College London, London, UK; 6 Cellular Metabolism and Microenvironment Laboratory, de Duve Institute, UCLouvain, Brussels, Belgium; 7 Hematology and Transfusion Medicine Center, University of Campinas, Campinas, Brazil; 8 Hematology Division, LIM31, Faculdade de Medicina, University of São Paulo, São Paulo, Brazil

**Keywords:** Haematological cancer, Cancer metabolism

## Abstract

Metabolic reprogramming is a hallmark of cancer, with acute myeloid leukemia (AML) being no exception. Mitochondrial function, particularly its role in protecting tumor cells against chemotherapy, is of significant interest in AML chemoresistance. In this study, we identified mitochondrial DNA content (mtDNAc), measured by quantitative PCR, as a simple and precise marker to stratify the metabolic states of AML patients. We show that patients with high mtDNAc are associated with increased mitochondrial metabolism and a higher dependency on oxidative phosphorylation (OXPHOS), often correlating with chemoresistance. Clinically, patients receiving cytarabine and an anthracycline-based regimen (7 + 3 regimen) experienced inferior relapse-free survival and a higher overall rate of leukemia recurrence. Ex vivo experiments using primary AML samples confirmed cytarabine resistance in high mtDNAc patients, which could be overcome by inhibiting mitochondrial complex I. The FDA-approved drug metformin, which targets mitochondrial metabolism, significantly enhanced apoptosis in response to chemotherapy or targeted agents, such as venetoclax, in AML models. However, metformin-treated cells adapted by increasing glycolysis and NAD^+^ production, a resistance mechanism that could be bypassed by targeting the nicotinamide phosphoribosyltransferase (NAMPT) enzyme. In summary, we demonstrated that mtDNAc is an effective tool for assessing the metabolic state of AML cells. This method can be easily implemented in clinical practice to identify chemoresistant patients and guide personalized treatment strategies, including novel combination therapies for those with a high reliance on mitochondrial metabolism.

## Introduction

Acute myeloid leukemia (AML) is a heterogeneous disease driven by genomic abnormalities acquired in myeloid precursor cells, leading to their rapid expansion in the bone marrow (BM).^1^ Despite improvements in remission rates over the last decades, a substantial number of young patients (10–40%) either fail to respond to induction therapy^2–4^ or experience relapse.^2,5,6^

Over the years, many reports have described several mechanisms by which leukemic stem cells (LSC) expand in the BM and escape standard treatment protocols.^7–9^ Amongst others, LSCs have been reported to rewire and adapt their metabolism to meet specific energetic requirements, thereby driving relapse.^10,11^ Furthermore, metabolic reprogramming is often linked to distinct driver mutations, such as *isocitrate dehydrogenase 1* and *2* (*IDH1*/*2*)^12^ and *fms-like tyrosine kinase 3* internal tandem duplication (*FLT3*-ITD). Within this context, we showed that *FLT3*-ITD-mutated AML cells can use lactate as a carbon source to fuel the tricarboxylic acid (TCA) cycle. Consequently, dual inhibition of the lactate transporter MCT1 (encoded by the *SLC16A1* gene) and the electron transport chain (ETC) complex II had strong anti-leukemic effects for this subset of patients.^13^

Recognized as the cell’s energy production site and the driving force of oxidative phosphorylation (OXPHOS), mitochondria are essential for the maintenance of cellular metabolic activity.^14^ The subunits of the mitochondrial ETC complexes are partly (13 in total) encoded by the circular double-stranded mitochondrial DNA (mtDNA), which ensures proper functioning of the respiratory chain. The amount of mtDNA copies per cell, known as the mtDNA content (mtDNAc), can vary, and, like the genomic DNA, mtDNA is prone to replication errors.^15^ To date, qualitative and quantitative alterations of mtDNA have been reported across various types of human cancers, with changes in mtDNA being tissue-dependent. As a result, both increased and reduced mtDNAc have been linked to tumor progression and unfavorable clinical outcomes.^16–20^ For instance, in pediatric AML^21–23^ and non-Hodgkin lymphoma,^24^ patients with high mtDNAc measured in BM mononuclear cells (BMMCs) and whole blood, respectively, exhibited poor prognosis. Contrarily, our group showed that patients with acute promyelocytic leukemia (APL) harboring high mtDNAc in BMMCs at diagnosis were associated with better clinical outcomes.^25^

Here, we demonstrate that high mtDNAc is linked to increased OXPHOS metabolism and poor clinical outcome of non-APL AML patients treated with intensive chemotherapy. We show that primary AML samples with high mtDNAc are more resistant to cytarabine-induced apoptosis in vitro. However, this resistance could be counteracted by the administration of metformin, which downregulates the mitochondrial metabolism. Metformin decreases mitochondrial metabolism in AML cells, thereby increasing chemotherapy-mediated cytotoxicity. However, metabolic plasticity allows AML cells to rewire their metabolism to increase glycolysis and NAD^+^ production (via up-regulation of the nicotinamide phosphoribosyl transferase, NAMPT) upon metformin treatment. The observed metabolic adaptation can be circumvented through the inhibition of the NAMPT by KPT-9274. Altogether, we propose measuring the mtDNAc as a simple and practical readout to determine the metabolic state of AML cells to develop personalized treatment strategies.

## Results

### High mtDNAc drives a more OXPHOS-like metabolic state in AML

**Fig. 1:**
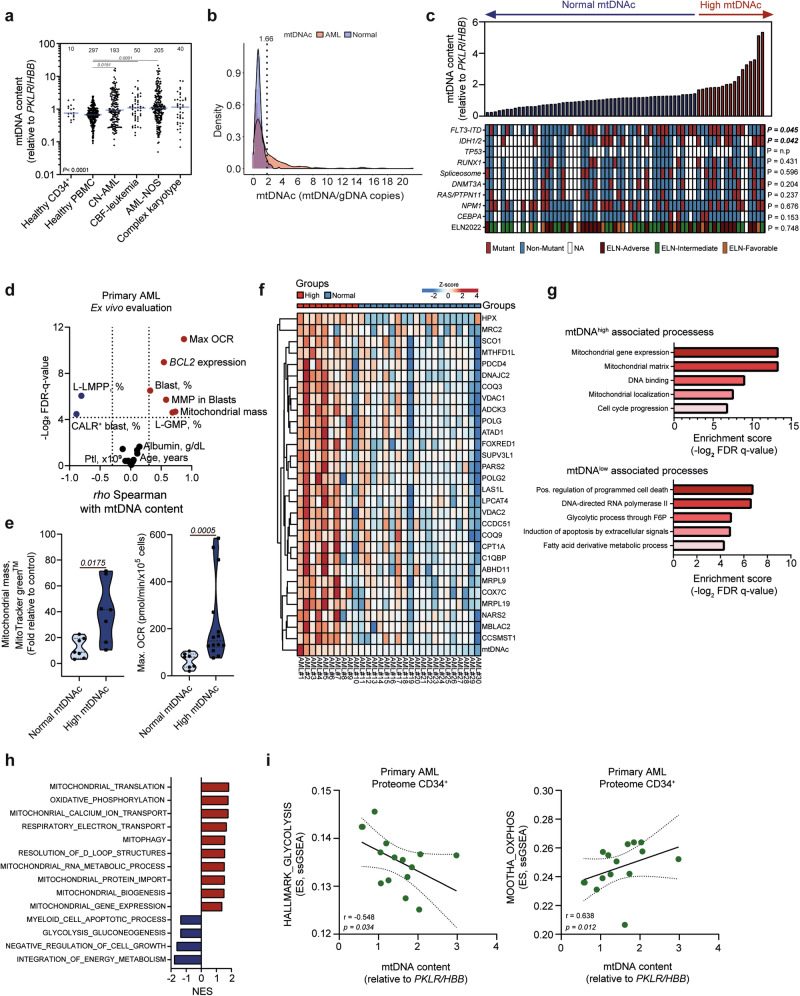
mitochondrial DNA content (mtDNAc) identifies patients with increased mitochondrial metabolism in AML. **a** Relative quantification of mtDNAc was conducted using quantitative real-time PCR, normalized to the single-copy nuclear genes (*PKLR* and *HBB*), in the training cohort (n = 482 AML samples, n = 297 healthy PBMCs, and n = 10 healthy CD34^+^). Horizontal bars indicate the median mtDNAc value. Numbers at the top represent the number of patients per group. Groups were compared using a Kruskal-Wallis H test with Dunn’s multiple comparison test. **b** Overlapped distribution of mtDNAc in healthy controls (displayed in blue, healthy CD34^+^ cells, n = 10 and healthy PBMCs, n = 297) and de novo AML patients (diagnosis samples) from the training cohort (red, n = 482). Y-axis displays the percentage of individuals included and X-axis the absolute mtDNAc quantification (mtDNA/gDNA copies). Dashed line represents the 95^th^ percentile of mtDNAc in the healthy controls, which was used as a cut point to identify AML patients with high mtDNAc. **c** Bar plot displaying the relative mtDNAc in primary AML cells included in the validation cohort (*n* = 70). Oncoprint displaying the baseline mutations and ELN2022-risk stratification of the patients with AML. NA, not available. For comparisons between two groups, the Mann–Whitney U test was used, while comparisons involving more than two groups were analyzed using the Kruskal–Wallis H test followed by Dunn’s multiple comparison post hoc test. **d** Spearman correlation of mtDNAc and ex vivo functionally evaluated parameters in primary AML samples (n = 67). Values were normalized by interexperimental control, and fold-relative to control values were inputted for correlation analysis. Blue and red dots indicate significant negative and positive correlations, respectively. L-LMPP, Lympho-myeloid primed progenitor; L-GMP, Granulocyte-Macrophage Progenitor; CALR, Calreticulin; OCR, oxygen consumption rate; MMP, mitochondrial membrane potential; Plt, Platelets. The comparison with blast percentage was based on the proportion of blasts assessed by the pathologist in bone marrow aspirates. **e** Violin plots illustrating the ex vivo assessment of mitochondrial mass (measured by MitoTracker Green™ staining) and maximum OCR (evaluated using the Seahorse XF96 Pro) in primary AML samples (n = 20), stratified based on their mtDNAc levels. Groups were compared using a Mann–Whitney U test. Proteomic landscape in AML patients regarding their mtDNAc: **f** Heatmap of the top 30 differentially expressed proteins between mtDNAc^low^ and mtDNAc^high^ patients analyzed by label-free quantitative proteome on sorted CD34^+^ AML cells (CD117^+^ for CD34^-^ samples, n = 30). Gene ontology (GO) **g** and gene set enrichment analysis (GSEA) (**h**) of mtDNAc^low^ and mtDNAc^high^ patients analyzed on the proteome of CD34^+^-sorted AML cells. NES, normalized enrichment score; FDR, false discovery rate. **i** Spearman correlation between mtDNAc and single sample GSEA (ssGSEA) enrichment scores (ES) obtained from proteome on sorted CD34^+^ AML cells

To better understand the role of mtDNAc in a hematological context, we compared the mtDNAc between healthy and leukemic samples. mtDNAc was analyzed in BM aspirates of 482 newly diagnosed AML patients (Brazilian cohort, hereafter called training cohort) along with 297 PBMCs samples from healthy age- and sex-matched subjects. Normalized mtDNAc levels (using a healthy control reference sample included in all experiments) ranged from 0.05 to 21.4 in AML samples and 0.1 to 2.45 in healthy volunteers, indicating that mtDNAc is significantly higher in AML patients, particularly those with normal karyotypes (NK), core binding factor (CBF), and not otherwise specified (NOS) AML (Fig. 1a). We found a positive correlation between mtDNAc from both BM and PB samples of the same patient (Pearson correlation coefficient, *r* = 0.79; supplementary Fig. 1a), indicating minimal bias when quantifying mtDNAc from different tissue sources. We validated our analyses in an independent cohort comprehending 105 consecutive patients diagnosed with AML and treated in the Netherlands (validation cohort), which was compared to healthy mobilized CD34^+^ cells (*n* = 10) (supplementary Fig. 1b). Both cohorts included patients with normal karyotypes, CBF, NOS, and complex karyotypes. No difference in mtDNAc was observed among the cytogenetically defined AML subgroups in both cohorts, also not in patients with cytogenetic alterations involving chromosomes 1 and 11, where *PKLR* and *HBB* genes are located, that were used as genomic DNA internal controls (Fig. 1a and supplementary Fig. 1b).

Of the 482 patients included in the training cohort, 163 patients (34%) displayed higher mtDNAc than the healthy control subjects (Fig. 1b). Likewise, in our validation cohort 35/105 patients (33% indicated by red dots in supplementary Fig. 1b) showed higher-than-normal mtDNAc. Using the previously described dichotomization strategy,^25^ we classified AML patients into two groups based on their mtDNAc levels: those with values above the 95^th^ percentile of healthy controls were categorized as high mtDNAc, while those within or below this threshold were categorized as normal mtDNAc (i.e., ≥1.66; Fig. 1b, dashed line). To establish a robust, healthy reference group, we included both healthy CD34^+^ cells and PBMCs. No significant differences were observed in mtDNAc levels between healthy CD34^+^ cells (median: 0.75, range: 0.39–1.9) and PBMCs (median: 0.67, range: 0.09-2.45). Based on this, we combined both healthy sample types into a single reference group for subsequent analysis. Baseline and clinical characteristics revealed that the frequency of *FLT3-*ITD and *IDH1/2* mutations was higher in patients with high mtDNAc (Fig. 1c and Supplementary Fig. 1c–d). To evaluate whether quantifying mtDNAc in BMMCs accurately reflects levels in the leukemic stem/progenitor cell fraction rather than a signal from the tumor microenvironment, we isolated CD34^+^/CD117^+^ cells (enriched for leukemic stem-progenitor cells, LSPCs) and measured the mtDNAc by qPCR. Our findings revealed a positive correlation between mtDNAc measured in BMMCs and CD34^+^ cells (r = 0.81), indicating that the quantification of mtDNAc in BMMCs reliably mirrors the mtDNAc levels in LSPCs (Supplementary Fig. 1e).

To investigate the functional characteristics and cellular processes associated with mtDNAc in AML, we conducted a series of analyses across different cell populations. Gene expression profiling focused on mitochondrial metabolism and drug resistance-related genes, while functional respiration experiments were performed on sorted CD34^+^ cells (or CD117^+^ for CD34^-^ AML cases). Additionally, bulk mononuclear cell fractions from AML patients were analyzed to assess correlations with immune-related factors, and all data were integrated with mtDNAc levels (Supplementary Fig 1f). Our results indicated that leukemic samples from patients with high mtDNAc were more granulocyte-monocyte progenitor (GMP)-like (L-GMP, defined by flow cytometry), with an augmented OXPHOS-driven metabolism coinciding with increased mitochondrial mass, mitochondrial membrane potential (MMP), functional respiration (oxygen consumption rate, OCR), and expression of *BCL2* (associated with chemoresistance in AML^26–28^). Contrarily, patients with low mtDNAc presented with more immature progenitor cell phenotypes, associated with higher dependency on glycolysis^28,29^ (Fig. 1d–e and Supplementary Table S1).

Mutations in genes associated with altered mitochondrial metabolism (*FLT3*-ITD and *IDH1/2*^13,30^), higher frequency of L-GMP type, and increased functional respiration capacity were frequent in AML patients with high mtDNAc. To better understand the metabolic state of leukemic cells with high mtDNAc, we performed an unsupervised label-free quantitative proteome (n = 30) analysis of CD34^+^ sorted cells (or CD117^+^, for CD34^-^ samples) from AML patients with different levels of mtDNAc using LC-MS. We identified 11272 annotated and confirmed proteins. At the protein level, gene ontology (GO) and gene set enrichment analysis (GSEA) associated high mtDNAc patients with the terms “MITOCHONDRIAL_GENE_EXPRESSION/TRANSLATION”, “OXIDATIVE_PHOSPHORYLATION”, and “CELL_CYCLE_PROGRESSION”, while patients with low mtDNAc were enriched for processes such as “POSITIVE_REGULATION_OF_PROGRAMMED_CELL_DEATH”, “MYELOID_CELL_APOPTOTIC_PROCESS”, “NEGATIVE_REGULATION_OF_CELL_GROWTH” and “GLYCOLYSIS_GLYCONEOGENESIS” (Fig. 1f–h). Furthermore, using single-sample GSEA (ssGSEA) analysis, we observed a negative and positive correlation between mtDNAc and the processes “HALLMARK_GLYCOLYSIS” and “MOOTHA_OXPHOS”, respectively (Fig. 1i). Additionally, deconvolution analysis using CIBERSORTx^31^ of the proteomic data confirmed that patients with high mtDNAc were more GMP-like, commonly linked to a more OXPHOS-driven metabolism.^13^ AML cell lines (*n* = 18) presented a high heterogeneity in mtDNAc (Supplementary Fig. 1g) and a positive correlation between mtDNAc and MMP, as well as the maximum OCR in AML cell lines (Supplementary Fig. 1h). Lactate production was negatively correlated with mtDNAc levels, suggesting decreased glycolysis in mtDNAc high AML cell lines. Transcriptome analysis of the AML cell lines^32^ suggested an up-regulation of OXPHOS-related genes in AML cell lines with high mtDNAc, while AML cell lines with low mtDNAc were linked increased expression of apoptosis-inducing genes (Supplementary Fig. 1i), which was further confirmed by GSEA (Supplementary Fig. 1j).

Metabolome data on AML cell lines^33^ indicated high mtDNAc to be associated with fatty acid (FA) degradation (possibly due to active FA oxidation) (Supplementary Fig. 1k–l). Overall, these results suggest that AML cells with high mtDNAc predominantly rely on OXPHOS metabolism, which can be fueled by multiple carbon sources, including FA metabolism.

### High mtDNAc is associated with poor outcomes and can refine AML risk stratification

**Fig. 2:**
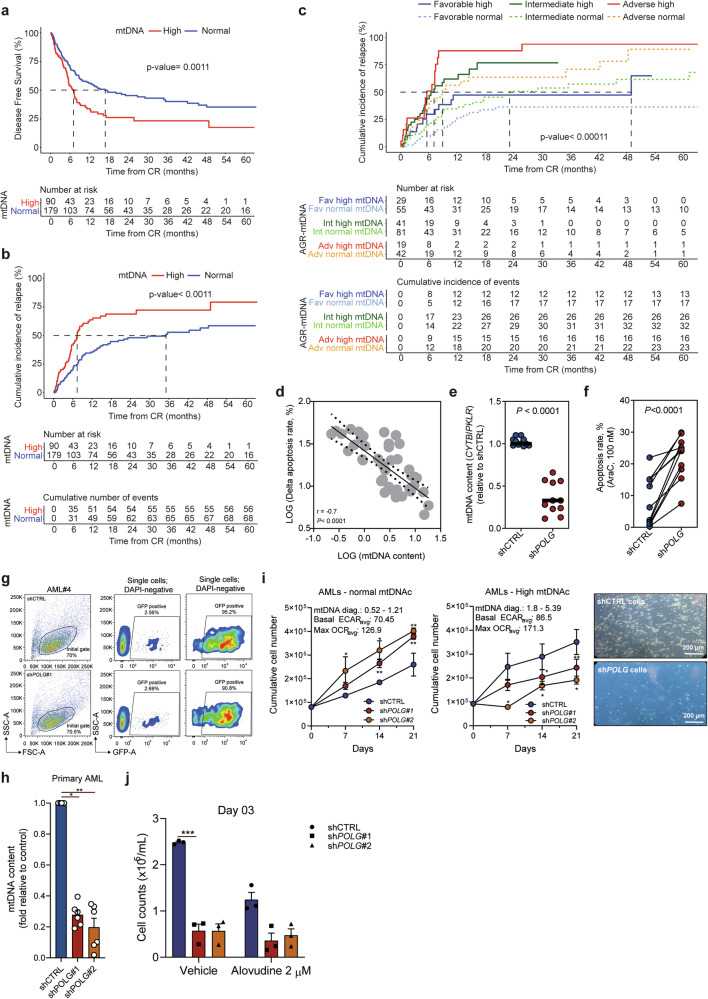
AML cells with high mtDNAc display resistance to cytarabine-induced apoptosis. **a** Disease-free survival (DFS) was assessed in AML patients with high mtDNAc (n = 90) *versus* those with normal mtDNAc, (n = 179), all treated with 3 + 7 based protocols (training cohort). DFS curves were generated using the Kaplan–Meier method, and differences were evaluated with the log-rank test. **b** Cumulative incidence of relapse (CIR) was analyzed considering relapse and non-relapse mortality as competing events. Time to relapse and non-relapse death was calculated from the date of complete remission. **c** CIR was further evaluated based on mtDNAc levels within the Adapted genetic risk (AGR) categories. Patients in each risk group (favorable, intermediate, and adverse) were stratified by mtDNAc status (normal and high mtDNAc). For **a**–**c** the numbers under the *X*-axis indicate the number of patients included in each comparison. **d** Spearman correlation between the ex vivo cytarabine (AraC) induced apoptosis (100 nM, 48 h) and the mtDNAc measured on AML blast population (training cohort, n = 44, SSC^low^CD45^dim^CD33^+^ population). Logarithmic values of the mtDNAc and apoptosis rate were used in the correlations analyses to better fit the data. Delta apoptosis rate was calculated by subtracting the AraC-induced apoptosis to the respective vehicle control. Dot plot panels displaying the mtDNAc (**e**) and the AraC-induced apoptosis (100 nM, 48 h) **f** in primary AML blasts transduced with sh*POLG* (using a single construct with five independent shRNAs targeting the *POLG* gene) and shCTRL as a control (training cohort, n = 11). Cells were cultured in liquid culture conditions for apoptosis assays. Groups were compared using a Mann–Whitney U test. Flow cytometry panels displaying the efficiency of transduction for shCTRL and sh*POLG*#1 in primary AML cells (AML#4) pre- and post-cellular sorting (**g**). Bar plot displaying the mtDNAc **h** in an independent cohort of primary AML blasts transduced with sh*POLG#1* and sh*POLG#2* (sequences indicated in the Supplemental methods) and shCTRL as a control (validation cohort, n = 6). Groups were compared using a Kruskal–Wallis H test with Dunn’s multiple comparison test. **i** Cumulative cell count of transduced primary AML cells (sh*POLG#1* and sh*POLG#2*/shCTRL) cultured for 21 days (n = 3 patient samples for the AMLs with normal and n = 3 for the AMLs with high mtDNAc). Representative pictures from the culture conditions for AML#6 are displayed on the right side. OCR, oxygen consumption rate; ECAR, extracellular acidification rate. **j** Viable cell counts of primary AML cells transduced with sh*POLG*#1, sh*POLG*#2, and shCTRL treated with alovudine (2 µM) for 72 h. Plots display the mean ± standard error of the mean (SEM). Groups were compared using Mixed-effect analysis with Dunn’s multiple comparison test

Next, we evaluated the prognostic impact of mtDNAc in AML patients treated with intensive “7 + 3 regimen” cytotoxic chemotherapy. Out of 482 patients included in our training cohort, 411 underwent this treatment (median age: 71 years, range: 18-93 years). Follow-up information for patients was last revised in July 2023. The median follow-up among survivors was 29 months (95% CI:25–35 months). Overall, 281 out of 411 patients (68%) achieved complete hematological remission (CR), with no significant association observed between mtDNAc and CR status (*P* = 0.478). Patients with high mtDNAc exhibited a lower 3-year disease-free survival (DFS) rate (28%, 95% CI:18–42%) compared to those with normal mtDNAc (47%, 95% CI:38–58%) (Fig. 2a and Supplementary Table S2). This result was consistent in multivariate proportional hazards analysis, where mtDNAc was used as either a categorical (Supplementary Fig. 2a) or continuous variable (Supplementary Fig. 2b), and considering age, leukocyte counts, and genetically defined risk category as confounders.^34^ When accounting for non-relapse mortality as a competing risk, patients with high mtDNAc showed an increased 3-year cumulative incidence of relapse (CIR) rate (Fig. 2b). Due to financial constraints that make implementing next-generation sequencing for ELN2022 stratification challenging in low- and middle-income countries (LMICs), we opted for an alternative risk stratification scheme.^34^ Therefore, we subclassified the training cohort according to clinical outcomes and evaluated the prognostic impact of mtDNAc on CIR separately in patients assigned to the favorable (90 patients), intermediate (128 patients), and adverse risk groups (61 patients). Patients with high mtDNAc were associated with poorer CIR regardless of their genetically defined risk classification (Fig. 2c). Collectively, these results suggest that high mtDNAc is associated to relapse risk rather than induction for conventionally treated patients, primarily impacting relapse rates rather than the initial response to induction therapy in AML.

To validate these findings in an independent patient cohort, we assessed the prognostic impact of mtDNAc in 42 patients treated with intensive chemotherapy at the University Medical Center Groningen, the Netherlands (validation cohort, median age: 56 years, range: 18–86 years). Again, mtDNAc had no impact on CR achievement (*P* = 0.563), but patients with high mtDNAc had a significantly lower DFS (33%, 95% CI:16–37%) when compared to patients with normal mtDNAc (67%, 95% CI:66-99%) (supplementary Fig. 2c). Additionally, to determine whether mtDNAc levels can predict therapy response in non-3 + 7 regimens, we analyzed the prognostic role of mtDNAc in patients treated with hypomethylating agents (HMAs; Decitabine or 5-Azacytidine, n = 9), which revealed no significant impact on prognosis (Supplementary Fig. 2d). Due to the limited sample size, multivariate proportional hazards analysis was not conducted. Further studies with larger patient cohorts treated with these regimens are necessary to draw more robust conclusions.

Finally, to benchmark our mtDNAc-based stratification strategy against established prognostic indicators in AML—molecular risk stratification AGR (training cohort) and ELN2022 (validation cohort)—we conducted area under the curve (AUC) analyses to compare predictive efficacy. Incorporating mtDNAc into multivariate logistic regression models improved survival prediction accuracy compared to molecular risk alone (training cohort: AUROC_mtDNAc_ = 0.67 *vs*. AUROC_AGR_ = 0.62; validation cohort: AUROC_mtDNAc_ = 0.66 *vs*. AUROC_ELN2022_ = 0.61) (Supplementary Fig 2e–f). Altogether, these findings indicate that mtDNAc is associated with increased mitochondrial activity in leukemic cells, which correlates with chemoresistance in AML patients treated with intensive chemotherapy.

### AML cells with high mtDNAc are more resistant to drug induced apoptosis

Given the worse clinical outcome of AML patients with high mtDNAc when treated with 7 + 3 chemotherapy, we decided to study how mtDNAc affects the response of AML cells towards cytarabine (AraC). Ex vivo analysis of primary AML cells treated for 24 hours with 100 nM AraC indicated increased resistance to drug-induced apoptosis in samples with high mtDNAc (Fig. 2d). To validate the link between mtDNAc and increased drug resistance, we performed a genetic knockdown (KD) of the DNA polymerase gamma (*POLG*) gene (through the usage of sh*POLG*) in primary AML cells included in the training cohort (n = 11), which encodes the catalytic subunit of the mitochondrial DNA polymerase. *POLG*-KD resulted in decrease mtDNAc (Fig. 2e) and enhanced the sensitivity to AraC-induced apoptosis compared to their respective control cells (Fig. 2f).

In our validation cohort, *POLG*-KD decreased proliferation rate of primary AML cells with high mtDNAc at baseline, which were in an OXPHOS^high^ state, as indicated by higher OCR values (Fig. 2g–i). Treatment with the POLG inhibitor Alovudine^35^ significantly reduced viable cell counts in scrambled control cells, whereas this effect was absent in shPOLG-transduced cells (Fig. 2j). Overall, these data suggest that mtDNA regulation participates in the dependence of mitochondrial metabolism and that its targeting could represent an effective adjuvant therapeutic option.

### Treatment with complex I inhibitor metformin improves the anti-leukemic effects of conventional AML therapies

Next, we aimed to identify a compound that can block mitochondrial activity to overcome the chemoresistance of AML cells with high mtDNAc. Therefore, we extracted the drug sensitivity scores generated in AML cell lines, for compounds with reported efficacy in AML models deposited in the Depmap portal (n = 30 compounds), and the dataset generated by Lee et al (n = 159 compounds).^36^ Amongst the different drugs, we found that AML cells with high mtDNAc were particularly sensitive to mitocans (drugs that target the mitochondrial metabolism^4,37^) and topoisomerase II inhibitors (supplementary Fig. 3a, b). Since mtDNA encodes various subunits of the ETC, including complex I, we sought to investigate if the complex I inhibitor rotenone could increase the sensitivity to commonly used chemotherapeutic agents in AML. Rotenone treatment (50 nM) increased AraC induced apoptosis in a subset of AML cell lines (MOLM13, and NB4), while it rescued its sensitivity in others (OCI-AML3 and HL60, Supplementary Fig. 3c). We noticed that the additive/antagonistic effect of rotenone in combination with AraC was linked to the primary metabolic dependencies of the tested AML cells (with OCI-AML3 and HL60 being more dependent on glycolysis than MOLM13 cells^28^ Supplementary Fig. 1g). Furthermore, regardless of their primary metabolic dependency, rotenone combined with venetoclax (VEN, a BCL2 inhibitor) showed strong synergistic effects in all tested AML models, which was validated in an ex vivo drug screen using primary AML cells (Supplementary Fig. 3d). Finally, patients with increased mitochondrial metabolism and/or with mutations in *IDH1*/*2* genes, were among the most sensitive to rotenone induced complex I inhibition (Supplementary Fig. 3e, f). These results confirmed that complex I inhibition can increase VEN sensitivity in AML.

**Fig. 3:**
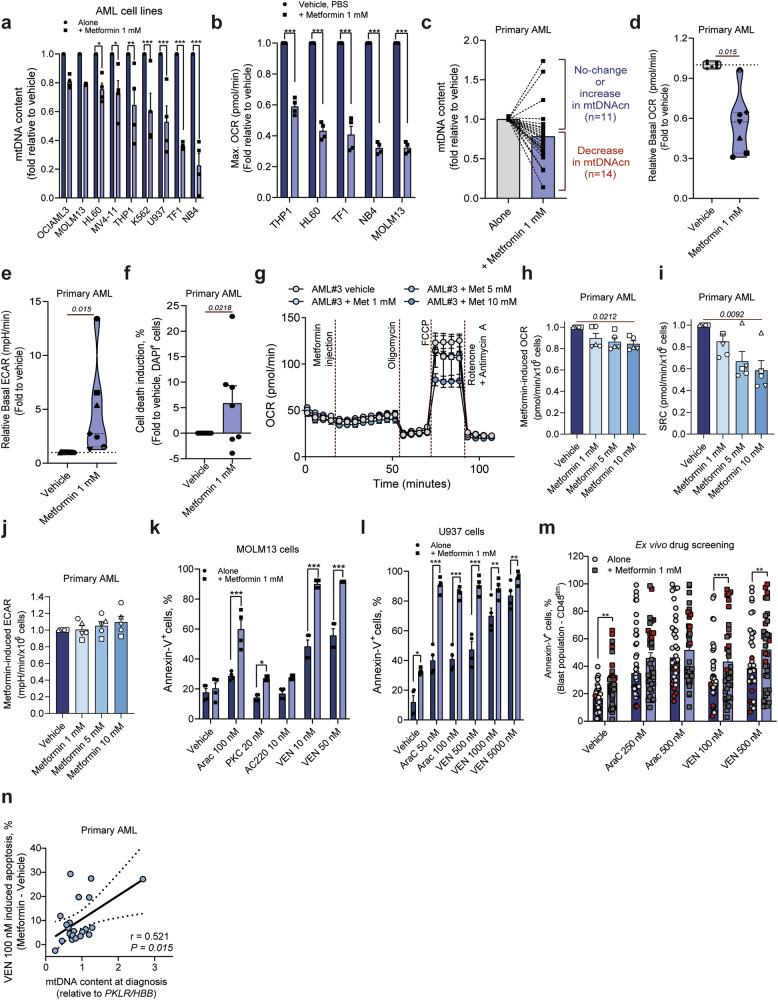
Metformin treatment reduces mtDNAc and mitochondrial metabolism, increasing venetoclax-induced apoptosis in AML cells. Comparison of mtDNA content (**a**) and maximum oxygen consumption rate (max OCR, **b**) between AML cell lines treated with metformin (1 mM, 48 h) or vehicle control. Bar graphs display values normalized to vehicle-treated controls, expressed as percentages for each sample. Data are shown as the mean ± standard error of the mean (SEM) from a minimum of three independent experiments. Groups were compared using Mixed-effect analysis with Dunn’s multiple comparison test. Comparison in ex vivo treated primary AML samples of mtDNA content (**c**), basal OCR (**d**), basal extracellular acidification rate (ECAR) (**e**) and cell death induction (**f**) upon metformin treatment (1 mM, 48 h) or vehicle control. Bar graphs and violin plots represent normalized values expressed as a fold for each sample (represented by a different symbol), normalized to vehicle-treated controls. Data are shown as the mean ± SEM from a minimum of four technical measurements. Groups were compared using a Mann–Whitney U test. **g** Real-time functional respiration analysis of primary AML samples (n = 5) following metformin injection (1, 5, and 10 mM) and vehicle control (PBS), with subsequent addition of compounds to assess mitochondrial respiration under various stress conditions (indicated in the Figure). Bar plots show metformin-induced changes in OCR (difference between measurement 12 and measurement 4, **h** and spare reserve capacity (SRC, **i**) in primary AML samples treated with 1, 5, and 10 mM metformin. Data are presented as mean ± SEM from at least four technical replicates. **j** Metformin-induced ECAR changes (difference between measurement 12 and measurement 4) were analyzed in primary AML samples using the same experimental setup as in panel g. Groups were compared using Kruskal–Wallis H test with Dunn’s multiple comparison test. Drug-induced apoptosis in MOLM13 (**k**) and U937 (**l**), treated with several AML-related drugs (VEN, venetoclax; AraC, cytarabine; PKC, midostaurin and AC220, quizartinib) in the presence or absence of metformin (1 mM, 72 h) detected by flow cytometry using an APC-annexin V/DAPI staining method. (**m**) Apoptosis was assessed by flow cytometry in gated human CD45^dim^CD34^+^ (or CD117^+^ cells for CD34^-^ AML samples) following ex vivo treatment in a co-culture system, using FITC-annexin V/DAPI staining. Cells were treated for 72 h with vehicle, AraC (250 and 500 nM), VEN (100 and 500 nM), with or without metformin (1 mM). Patients displaying high mtDNAc levels (>1.66) are marked in red. Bar graphs show the mean ± SEM from all independently tested patients, with each dot representing an individual patient sample. Statistical significance and cell lines are indicated on the graphs; *p < 0.05; **p < 0.01; ***p < 0.001. Groups were compared using Mixed-effect analysis with Dunn’s multiple comparison test. **n** Spearman correlation between mtDNAc and VEN 100 nM + Metformin induced apoptosis (relative to metformin monotherapy control) on primary CD34^+^ AML cells. The number of biological replicates is indicated by the dots on the plots. Each biological replicate is an average of at least two independent technical replicates

Next, we evaluated the effect of the FDA-approved biguanide metformin, used as a first-line treatment for type 2 diabetes with known downregulation of mitochondrial activity.^30,38^ Like for rotenone, treatment with low dose of metformin (1 mM, 24 h) reduced the mtDNAc in a panel of AML cell lines (Fig. 3a), functionally associated with a decrease in oxygen consumption rate (OCR) (Fig. 3b). This was further validated in a panel of primary AML samples (reduction in 14/25 samples, 56%), where we observed also a decrease in OCR, with an increase in the extracellular acidification rate (ECAR) suggesting metabolic rewiring from OXPHOS to glycolysis as a compensatory mechanism correlating with metformin sensitivity (Fig. 3c, f and supplementary Fig. 3g, h). Metformin has previously been linked to the downregulation of complex I activity in cancer.^39,40^ To assess whether metformin directly inhibits mitochondrial respiration, we performed real-time functional respiration analysis on primary AML cells following incremental metformin administration. No immediate or short-term reduction in OCR was observed upon metformin treatment (Fig. 3g, h). However, mitochondrial stress testing with the OXPHOS uncoupler FCCP revealed a decreased spare respiratory capacity (SRC) in cells treated with higher doses of metformin (5 and 10 mM) compared to a low dose (1 mM) (Fig. 3i). Additionally, no immediate modulation of ECAR was detected upon metformin injection (Fig. 3j), suggesting that metformin-induced metabolic reprogramming toward glycolysis occurs in a time-dependent manner. Collectively, these findings indicate that metformin may impair mitochondrial respiration under stress, potentially offering a strategy to target chemoresistant leukemic cells.

We then investigated whether prolonged treatment with a low dose of metformin (1 mM) for 24, 48, and 72 h could induce cell cycle arrest or apoptosis, potentially explaining the reduced mitochondrial respiration observed in our extracellular flux analysis. However, in a panel of three independent AML cell lines, metformin treatment did not alter cell cycle phase distribution compared to the vehicle control (Supplementary Fig. 3i). Treatment of AML cell lines with AraC, FLT3-inhibitors (midostaurin, PKC and quizartinib, AC220), and VEN in combination with lower dose of metformin significantly increased drug induced apoptosis in all AML models (Fig. 3k–l and supplementary Fig. 3j). In our ex vivo treated primary AML cohort (n = 36 patients), we observed a high heterogeneity in cell death when metformin was combined with AraC, whereby some patients (with high mtDNAc) displayed strong additive effects (9/36 samples, 25%). In the context of VEN, the combination with metformin resulted in a significant increase in drug-induced apoptosis (Fig. 3m, n). Altogether, our data suggests mtDNAc evaluation as a cost-effective predictive tool for drug responses targeting mitochondrial metabolism.

### Metformin combination overcomes venetoclax resistance in AML

Although the introduction of VEN significantly improved the clinical outcome of AML patients ineligible for intensive chemotherapy, a subgroup of patients remains resistant.^41^ Among the most frequent causes associated with poor responses are mutational background,^42,43^ differentiation stage,^44,45^, and enhanced mitochondrial metabolism.^46–48^ Since our results showed increased sensitivity of primary AML samples when treated with metformin in combination with VEN we questioned whether metformin could help overcome VEN resistance. In accordance with previous reports, venetoclax ED_50_ analysis indicated heterogenous responses across AML cell lines, with K562, OCIAML3, and U937 being the most resistant lines (supplementary Fig. 4a). In K562 cells, treatment with a high dose of VEN (1 µM) had no effect on OCR, while metformin alone (1 mM) or in combination with VEN significantly decreased the basal and maximum cellular respiration. Metformin monotherapy slightly increased the ECAR (supplementary Fig. 4b, c). Similar trends regarding the reduction of mitochondrial metabolism were observed in U937 cells (which are more sensitive to VEN in comparison to K562 cells), with a time-dependent decreased in their spare reserve (SRC) capacity upon metformin treatment (supplementary Fig. 4d–j). Employing the Zero Interaction Potency (ZIP) model, we identified a synergistic interaction between metformin and VEN in U937 cells across most dose combinations tested (ZIP score = 10.6). However, in K562 cells, which are known for their high resistance to VEN (average ZIP score: 3.6), synergistic effects with metformin were evident solely at the highest VEN concentration (30 µM) (supplementary Fig. 4k, l).

To further evaluate whether combining metformin with venetoclax enhances therapeutic efficacy in a VEN-resistant AML model, we transplanted K562 cells into NSG mice. Following confirmation of engraftment, mice were randomized into treatment groups and received venetoclax, metformin, or combination therapy. Notably, combination treatment did not impact total body weight, suggesting a favorable therapeutic index. While venetoclax and metformin monotherapies did not significantly alter human CD45^+^ levels in peripheral blood (PB) compared to vehicle-treated controls, the combination therapy significantly reduced leukemia burden in PB, bone marrow, and spleen relative to both vehicle and venetoclax monotherapy groups (Supplementary Fig. 4m–o). In primary AML cells, the effect of VEN monotherapy showed heterogenous results. Contrarily, metformin monotherapy or in combination with VEN consistently reduced mitochondrial respiration and ATP production in primary AML cells (Supplementary Fig. 4p, q). Consistent with our previous data, we also observed an increase in the ECAR upon metformin treatment in primary AML cells, suggesting metabolic rewiring from OXPHOS to glycolysis as a congruent compensatory mechanism in AML (Supplementary Fig. 4r, s).

### Combination therapy of KPT-9274 and metformin successfully targets AML cells with high mtDNAc

**Fig. 4:**
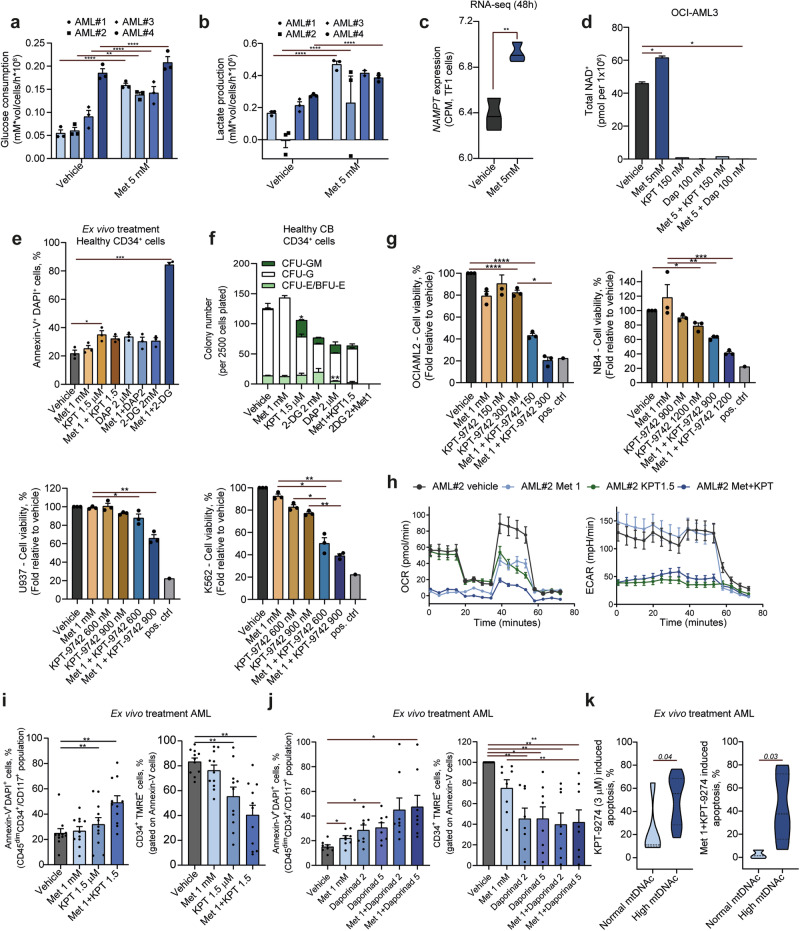
Metformin-induced metabolic rewiring can be overcome by NAMPT inhibition with KPT-9274 in primary AML blasts. Glucose consumption (**a**) and lactate secretion (**b**) rate at of primary AML cells after treatment with metformin (1 mM) and vehicle control for 48 h. Each dot represents an average of four technical replicates of an individual patient sample. Groups were compared using Mixed-effect analysis with Dunn’s multiple comparison test. **c** RNA-sequencing experiment showing *NAMPT* levels (CPM) on TF1 cells treated with metformin (5 mM, 48 h). Groups were compared using a Mann–Whitney U test. **d** Total NAD^+^ levels in OCI-AML3 cells treated with metformin (5 mM), KPT-9274 (150 nM), or daporinad (100 nM) alone or in combination for 48 h. Bars represent the average of 4 independent measurements. Neonatal cord blood (CB) CD34^+^ cells were treated ex vivo for 72 h with described drugs and concentrations, and apoptosis induction (**e**) and clonogenic (CFU-assay) capacity (**f**) were evaluated. For CFU-assay, cells were cultured in cytokine-enriched methylcellulose with either vehicle or the indicated drugs. Colonies were counted after 8–14 days, and results are expressed as a percentage relative to vehicle-treated controls. Bars represent the mean ± SD from a minimum of three assays. **g** Dose-response cytotoxicity was evaluated by Annexin-V/DAPI staining and flow cytometry in a set of myeloid leukemia cell lines (OCI-AML2, NB4, U937, and K562) after 72 h of treatment with specified drugs and concentrations. Results are presented as the percentage of viable cells (Annexin-V⁻/DAPI⁻) relative to vehicle-treated controls. A positive control (pos. ctrl) was included for each cell line (boiled cells up to 95 ˚°C for 30 minutes). Groups were compared using Mixed-effect analysis with Dunn’s multiple comparison test. **h** Oxygen consumption rate (OCR, left panel) was measured in vehicle- or metformin- (1 mM), KPT-9274- (1.5 μM) or combination treatment in primary blasts isolated from AML#2 (ELN2022-adverse with multiple mutations) using an extracellular flux analysis. A representative line graph showing oxygen consumption rate (OCR) following the sequential addition of oligomycin (Oligo A), carbonyl cyanide-p-trifluoromethoxyphenylhydrazone (FCCP), and rotenone combined with antimycin A (Rot + Anti A) is presented; OCR was recorded continuously over time. The extracellular acidification rate (ECAR, right panel) was assessed in the same samples used for OCR analysis using the Seahorse XF96 analyzer. A representative line graph depicts ECAR following the sequential addition of glucose, oligomycin (Oligo A), and 2-deoxy-D-glucose (2-DG); measurements were taken continuously over time. Data was displayed as mean ±95% confidence interval (CI) of three independent biological replicates plated in four independent technical replicates. Combination therapy between NAMPT inhibitors and metformin: (i) Bar plot illustrating apoptosis induction following treatment with metformin (1 mM), KPT-9274 (1.5 µM), and their combination. **j** Bar plot showing apoptosis induction upon treatment with metformin (1 mM), daporinad (2 and 5 µM), and their combination. Each dot represents an individual patient sample. TMRE staining was used to assess mitochondrial membrane potential in the samples analyzed in (**i**, **j**). **k** Apoptosis levels in ex vivo treated primary AML samples with KPT-9274 (3 µM, left panel) and KPT-9274 (1.5 µM) + metformin (1 mM, right panel) according to mtDNAc levels (normal mtDNAc = 5 patients and high mtDNAc = 9 patients). The number of biological replicates is indicated by the dots on the plots. Each biological replicate is an average of at least two independent technical replicates. For comparisons between two groups, the Mann–Whitney U test was used, while comparisons involving more than two groups were analyzed using the Kruskal–Wallis H test followed by Dunn’s multiple comparison post hoc test

Our extracellular flux analysis indicated that AML cells can rewire their metabolism upon treatment with metformin, possibly as a mechanism to overcome the reduction in mitochondrial energy generation (Fig. 3c–f and supplementary Fig. 4m). Functional respiration assays confirmed increased glucose consumption and lactate production rate in primary AML samples when treated with metformin (Fig. 4a-b). Given that inhibition of mitochondrial respiration by metformin dysregulate the NAD^+^/NADH homeostasis (disrupting the conversion of NADH to NAD^+^, with NADH accumulation and decreased NAD^+^ availability), resulting in compensatory *NAMPT* expression,^48,49^ we questioned whether the same mechanisms could be observed in AML cells. Transcriptomic analysis of metformin-treated TF1 AML cells revealed an increase in *NAMPT* expression upon metformin treatment (Fig. 4c). Subsequently, total intracellular measurements of NAD levels (NAD^+^ and NADH) showed an accumulation of NAD^+^ in OCI-AML3 cells upon metformin treatment, which was strongly diminished when OCI-AML3 cells were treated with the NAMPT inhibitors KPT-9274 and daporinad (±metformin) (Fig. 4d). We and others (Sternadt et al. *under review*), have shown that metformin treatment promotes ferroptosis in AML cells, particularly the ones carrying *FLT3* or *IDH1*/*2* mutations. Given that NAMPT inhibitors are also known to induce ferroptosis in AML,^49^ their combination with metformin could potentially enhance the therapeutic efficacy of metformin in this context.

These findings implied that the combination of metformin with Pyruvate dehydrogenase kinase 1 (PDK1) and Hexokinase-2 (HK2) (related to the glycolysis process) or NAMPT inhibitors could potentially intensify the cytotoxic effects of metformin by obstructing the metabolic rewiring capacity and potentiating the mechanism of action of metformin (associated with ferroptosis induction) of AML cells. First, we evaluated which drug combination would provide the best therapeutic window in the context of healthy hematopoiesis. Healthy cord blood (CB)-derived CD34^+^ cells were treated with the combinations tested in AML samples. Our results showed that while the monotherapies elicited minimal to no cytotoxic effects on healthy CD34^+^ cells, the combination of metformin (1 mM) plus 2-deoxyglucose (2-DG, 2 mM) exhibited strong cytotoxicity (Fig. 4e), also in colony assays (Fig. 4f). However, the combination of KPT-9274 with metformin displayed marginal effects on normal CD34^+^ cells (Fig. 4e, f). Therefore, we concluded that dual inhibition of mitochondrial respiration and NAMPT would provide the best therapeutic window. Thus, we treated several AML models with metformin in combination with KPT-9274, to avoid metabolic rewiring and further inhibit one of the main sources of energy production. The combination of metformin with KPT-9742 increased drug-induced apoptosis in all AML cell lines except for U937, where metformin only enhanced apoptosis when combined with KPT-9274 at the highest dosage (Fig. 4g). Functional respiration analysis indicated a decrease in OCR when primary AML samples were treated with metformin or KPT-9274 monotherapies as well as in combination, while the metformin-enhanced ECAR was now diminished upon KPT-9274 treatment (Fig. 4h). We next evaluated the efficacy of the combination therapies in our cohort of ex vivo treated primary AML samples (n = 11). Treatment with NAMPT inhibitors KPT-9274 (1.5 µM) or daporinad (2 and 5 µM) with metformin (1 mM) significantly increased apoptosis and reduced mitochondrial membrane potential in primary AML cells (Fig. 4i, j). Given the correlation between mtDNAc and the ex vivo responses to metformin and VEN, we wondered if mtDNAc levels could serve as a stratification tool to predict the response to KPT-9274/metformin combination regimen. Indeed, our results indicated that AML samples with high mtDNAc exhibited increased sensitivity to KPT-9274, particularly in combination with metformin, when compared to normal mtDNAc AML samples (Fig. 4k).

## Discussion

Here, we identified a new metabolic subgroup of patients with high mtDNAc associated with enhanced OXPHOS metabolism and increased resistance to chemotherapy, translating into poor clinical outcomes. We demonstrate that inhibition of mitochondrial respiration by metformin effectively re-sensitized AML cells with high mtDNAc to VEN and AraC-induced apoptosis. Furthermore, our findings underscore that the induction of apoptosis by metformin was significantly potentiated when combined with the NAMPT inhibitor KPT-9274, thereby impeding metabolic rewiring.

Analysis of mitochondrial genomes in human cancers revealed that patients with myeloid neoplasms have lower mtDNAc compared to most solid tumors, which generally exhibit a higher mtDNA mutational burden and greater dependence on OXPHOS metabolism.^50^ Of note, mutations in the electron transport chain (both germline and somatic) can influence mitochondrial number and/or mass, primarily through compensatory biogenesis mechanisms triggered by impaired respiration and elevated cellular stress. However, it is important to highlight that increased mitochondrial content does not necessarily indicate enhanced mitochondrial function, as the quality of these mitochondria may be compromised, underscoring that quantity alone does not reflect bioenergetic fitness.^12,14,50^ A link between OXPHOS and drug resistance has been suggested by several studies in solid tumors and in AML, whereby an OXPHOS-driven metabolism was associated with an up-regulation of the anti-apoptotic protein BCL2.^51–54^ Our results show an association between mtDNAc and *BCL2* levels, which can potentially contribute to chemoresistance. It is important to note that, while high mtDNAc levels are observed in patients with a more L-GMP phenotype and are associated with chemoresistance, our data simply define a new subgroup of AML patients with poor prognosis. This subgroup is not mutually exclusive with other markers, such as stem-like features, which are also linked to poor clinical response in AML patients.^28,55,56^ Moreover, we and others have shown that AML cells are able to hijack functional mitochondria from the niche, more specifically from mesenchymal stem cells as well as from immune cells such as macrophages, to protect themselves against chemotherapy.^29,57,58^ These data suggest that leukemic cells drive metabolic rewiring towards a more OXPHOS-like state in part via the increase of mtDNAc or by hijacking foreign mitochondria to withdraw energy and promote therapy resistance. In both cohorts, high mtDNAc was associated with chemoresistance and inferior DFS, despite no differences in CR rates. Although we have not explored this lack of association between mtDNAc and CR achievement, it is conservable that induction therapy can effectively eliminate bulk leukemia cells while sparing a small population of resistant clones that drive relapse.^59–61^ Additionally, CR does not account for minimal residual disease (MRD), which may be higher in high mtDNAc patients, contributing to inferior long-term outcomes despite similar initial remission rates.

Nowadays, VEN-based regimens are the most successful strategy used in the clinic for AML treatment by targeting metabolism-related features.^7,41,47,48^ However, relapse still occurs due to compensatory metabolic pathways upregulated in LSCs. In a study published by Jones et al. the authors showed that LSCs were reliant on amino acid metabolism to drive OXPHOS at diagnosis, while LSCs from relapse samples were able to rewire their metabolism towards increased fatty acid (FA) uptake.^62^ Other mechanisms that were reported to confer VEN resistance include the upregulation of nicotinamide metabolism.^48,63^ Together, these studies suggest that metabolic plasticity imposes a significant challenge to successfully eradicate LSCs.

We and others have previously shown that certain AML subtypes, such as those with *IDH1*/*2* and *FLT3*-ITD mutations, exhibit a greater dependence on mitochondrial metabolism.^12,13,30,64,65^ In particular, complex I inhibition by IACS-010759 selectively targets and eliminates the *IDH1*-mutant LSC population.^66^ However, a recent clinical trial evaluating IACS-010759 in solid tumors and AML raised significant concerns about the risks and feasibility of mitotoxic agents in cancer therapy.^10,67^ Given its well-established safety in clinical settings, metformin, when combined with cytotoxic chemotherapy or VEN-based regimens, may help mitigate the adverse effects commonly associated with metabolism-targeting therapies.

Early findings from the VenCM trial (NCT06537843), which investigates metformin in combination with venetoclax and cytarabine in relapsed/refractory AML, reported no grade 3-5 non-hematological toxicity and a 6-month overall survival rate of 68%, significantly higher than the historical median survival rate of 3 months.^68^ Notably, the metformin dose used in the VenCM trial (2550 mg/day) is substantially lower than the concentrations employed in our in vitro experiments, based on prior studies estimating metformin serum levels in prediabetic patients treated with 1500 mg/day.^69^ This discrepancy highlights the challenges in translating preclinical findings into clinical applications. Ongoing studies are exploring whether mtDNAc levels can improve patient stratification and predict responses to metformin-based therapeutic protocols.

Here, we show that metformin can successfully decrease mitochondrial metabolism and increase drug-induced apoptosis when combined with several anti-leukemic drugs, like AraC and VEN. We also noticed a decrease in mtDNAc in a subset of treated samples. This suggests a potential selection process, where cells with higher mtDNAc exhibit increased sensitivity to metformin, leaving behind predominantly those with lower mtDNAc levels following treatment. In line with previous reports, we observed that metformin-induced inhibition of mitochondrial metabolism led to a metabolic shift toward glycolysis.^39,70,71^ This metabolic adaptation may serve as a mechanism for cells to counteract the cytotoxic effects of metformin. In addition to the increase in glycolysis, we noted an increase in *NAMPT* expression and total NAD^+^ levels upon metformin treatment. A study published by Parisotto et al. suggested that NAD^+^/NADH homeostasis affects metformin sensitivity. As a result, blocking NAMPT with the NAMPT inhibitor FK866 decreased the NAD^+^ pool and enhanced metformin-mediated cytotoxicity by activating the p53 and oxidative stress pathways.^72^ Zabka et al demonstrated that several NAMPT inhibitors (APO-866, GMX-1777, and GMX-1778) induced retinal toxicity in rodents, potentially limiting their clinical applicability.^73^ However, the recent Phase 1 clinical trial NCT02702492, which evaluated the NAMPT inhibitor KPT-9274 in patients with advanced solid tumors, reported a low incidence of dose-limiting toxicities and adverse events, suggesting a potential therapeutic window for NAMPT inhibition. Furthermore, NAMPT inhibition selectively targeted AML blast cells with high mtDNAc, leading to metabolic collapse while sparing healthy HSPCs, suggesting a favorable therapeutic index.

One limitation of our study is the reliance on pharmacological interventions, introducing the possibility of unintended off-target effects. While we addressed this concern through rescue experiments and the use of structurally distinct compounds to emphasize target specificity, the potential for pleiotropic outcomes, especially with drugs like metformin, cannot be entirely ruled out. Second, the inclusion of samples for the functional assays was mainly dictated by the availability of viable cells in sufficient numbers to perform the described experiments. Third, it is clear that, compared to conventional qPCR as used here, higher precision methodology, such as digital droplet PCR, is currently available to quantify mtDNAc.^74^ While clearly more quantitative, such technology is also expensive and not available in all diagnostics centers worldwide, certainly not in middle- and low-income countries. Overall, we demonstrate that a simple and cost-effective method for measuring mtDNAc can effectively identify a subgroup of AML patients, within the high metabolic heterogeneity, who are dependent on OXPHOS metabolism and therefore more susceptible to mitochondria-targeting therapies. Incorporating mtDNAc as a prognostic marker could facilitate clinical decision-making when administering drugs targeting mitochondrial-related metabolic pathways. Finally, the combination of metformin and NAMPT inhibitor KPT-9274 could be an efficient therapeutic alternative for patients with high mtDNAc who are resistant to conventional chemotherapy.

## Materials and methods

### Study approval and patient samples

BM or peripheral blood (PB, for patients with unavailable BM aspirates) samples of AML patients used for mtDNAc analysis and for ex vivo functional experiments were studied after informed consent and protocol approval by the Ethical Committee in accordance with the Declaration of Helsinki (process number #5.536.539; CAAE: 47769821.7.0000.5208). Mononuclear cells (MNCs) from diagnostic samples were obtained via Ficoll density gradient separation (Sigma-Aldrich) and subsequently cryopreserved. Peripheral blood mononuclear cells (PBMCs) from healthy, sex- and age-matched donors were collected, and total genomic DNA was extracted from these isolated PBMCs.

### Determination of mtDNA content (mtDNAc) and gene expression analysis

Following total genomic DNA extraction (NucleoSpin Tissue, BiOkE, NL), mtDNAc was measured by real-time quantitative PCR (qPCR), targeting the mitochondrial cytochrome B (*CYTB*) gene as a marker for mtDNA. The nuclear DNA was quantified using primers for the single-copy nuclear gene pyruvate kinase (*PKLR*) and hemoglobin subunit beta (*HBB*).^25,75^

### Proteome of AML blasts and correlation analysis with the mtDNAc

Proteomic analyses were performed as previously described (available at PRIDE under PXD030463).^28^ The proteomic landscape of the sorted AML blasts was correlated with the mtDNAc of the same patient sample. Details on the statistical analysis can be found in the section—Gene Ontology (GO)/Gene Set Enrichment Analysis (GSEA).

### Cell lines

All cell cultures were maintained in a humidified atmosphere at 37°C with 5% CO2. Mycoplasma contamination was routinely tested. All leukemia cell lines were authenticated by short tandem repeat analysis. Cells were obtained from their correspondent biobank sources as stated in the resource table and were cultured according to the guidelines offered by the supplier. Cytarabine (AraC), rotenone (Rot), antimycin A (AA), oligomycin A (Oligo), 2-deoxy-D-glucose (2-DG), 2,2-dichloroacetophenone (DAP), midostaurin (PKC), and metformin were obtained from Sigma-Aldrich (St. Louis, USA). Venetoclax (VEN), quizartinib (AC220), and the NAMPT inhibitor KPT-9274 were obtained from Selleckchem (Houston, USA). (E)-Daporinad and alovudine were obtained from MedChemExpress (Groningen, NL).

### In vivo assays using K562 cells

For AML cell line-derived xenograft models, 8-week-old male and female NSG mice (005557, The Jackson Laboratory) were pre-conditioned with a single dose of busulfan (20 mg/kg, Sigma-Aldrich) one day before transplantation. The following day, 1 × 10⁵ K562 cells were intravenously injected via the tail vein. Seven days post-transplant, human CD45+ chimerism was assessed in the peripheral blood (PB), and based on engraftment levels and body weight, mice were randomly assigned to four treatment groups (n = 6 per group): vehicle, venetoclax (20 mg/kg in mineral oil, oral gavage, Sigma-Aldrich), metformin (125 mg/kg in PBS, intraperitoneally, Sigma-Aldrich), or the combination. Leukemic burden in the PB, leukocyte counts, and body weight were monitored weekly. Mice were euthanized upon reaching humane endpoints, including (1) a sustained weight loss of more than 15% for 2 consecutive days, (2) PB chimerism exceeding 40%, or (3) signs of impaired movement or visible distress due to disease progression. At termination, K562 cell chimerism (human viable CD45^+^) in the spleen, PB, and bone marrow was analyzed by flow cytometry. Only those with viable material (bone marrow or spleen) were included in subsequent chimerism and bone marrow count analyses. All animals were housed under specific pathogen-free conditions in individually ventilated cages and maintained in accordance with the Guide for the Care and Use of Laboratory Animals (National Research Council, USA) and the National Council of Animal Experiment Control guidelines. The study was approved by the Animal Ethics Committee of the University of São Paulo (#032/2021).

### Ex vivo drug screening in primary AML samples

Cryopreserved MNC fractions of AML patients were thawed and prepared as previously described in the section “Flow cytometry”, and resuspended in IMDM + 20% FCS, + 20 ng/mL of G-CSF, IL-3, and N-plate. Cells were plated at a cellular density of 1.5 million cells/mL for 48 h, to remove cellular debris that remained after the thawing procedure. For the ex vivo drug screening, cells were washed once in IMDM + 20% FCS and plated at 1.5 × 105 cells/mL in 48-well plates and treated with a dose-range of the different compounds (described in the Fig. legends) used to evaluate the cytotoxic effects on leukemic blasts. To analyze the cytotoxicity in the different fractions of the bulk treated AML cells, treated MNCs were blocked with human FcR blocking reagent (Miltenyi Biotec) for 5 min and stained with the following antibodies: CD45-APC-Cy7, TMRE, CD14-PerCP, CD34-Pe-Cy7 (or CD117-PE for CD34^-^ samples), and CD11b-APC for 20 min at 37 °C. After incubation, cells were washed once in PBS and at the end resuspended in IMDM + 20% FCS supplemented with 10% of Ca^2+^ buffer (10X, BD biosciences, CA, USA) plus Annexin-V FITC (Biolegend, CA, USA) and the viability marker DAPI. Fluorescence was measured on the BD LSRII and analyzed using Flow Jo (Tree Star, Inc.). The apoptosis induction and modulation of the mitochondrial membrane potential were evaluated in the leukemic blast population (CD34^+^ or CD117^+^). A more mature myeloid population was detected based on the CD45 staining positive for CD14 and CD11b and negative for CD34/CD117.

Further information is provided in the Supplementary Materials and Methods

## Supplementary information

Supplementary Materials

## Data Availability

All the datasets presented in the paper are available in the supplemental material or deposited in the indicated repositories as described in the methods section. All RNA sequencing data supporting this study are available for download from the King’s Open Research Data System (KORDS).
