# Supplementary material for: High mtDNA content identifies oxidative phosphorylation-driven acute myeloid leukemias and represents a therapeutic vulnerability
Source: Signal Transduct Target Ther. 2025 Jul 14;10:222. doi: 10.1038/s41392-025-02303-x (PMC12256626; doi:10.1038/s41392-025-02303-x)
Supplement: Supplementary file 1 — Supplementary Materials [file 41392_2025_2303_MOESM1_ESM.docx]

Supplementary Materials for

High mtDNA content identifies oxidative phosphorylation-driven acute myeloid leukemias and represents a therapeutic vulnerability

Diego A Pereira-Martins,^1,2,3^ Isabel Weinhäuser,^2,3^ Emmanuel Griessinger,^2^ Juan L Coelho-Silva,^1,3^ Douglas R Silveira,^4^ Dominique Sternadt,^2^ Ayşegül Erdem,^2,5^ Bruno Kosa L Duarte,^6^ Prodromos Chatzikyriakou,^4^ Lynn Quek,^4^ Antonio Bruno Alves-Silva,^3^ Fabiola Traina,^3^ Sara T Olalla Saad,^6^ Jacobien R Hilberink,^2^ Amanda Moreira-Aguiar,^1^ Maria L Salustiano-Bandeira,^1,7^ Marinus M Lima,^1^ Pedro L Franca-Neto,^1^ Marcos A Bezerra,^1^ Nisha K van der Meer, ^2^ Emanuele Ammatuna,^2^ Eduardo M Rego,^3,7^ Gerwin Huls,^2^ Jan Jacob Schuringa,^2*^ Antonio R Lucena-Araujo^1*^

Correspondence to: [j.j.schuringa@umcg.nl](mailto:j.j.schuringa@umcg.nl)

**This PDF file includes:**

Materials and Methods

Supplementary Text

Figures. S1 to S4

Tables S1 to S2

Materials and Methods

Resources table

| **REAGENT or RESOURCE** | **SOURCE** | **IDENTIFIER** |
| --- | --- | --- |
| **Antibodies** | | |
| Anti-Human CD45 FITC (1:50 dilution) | BioLegend | 368508 |
| Anti-Human CD45 APC-Cy7 (1:100 dilution) | BioLegend | 304014 |
| Anti-Human CD14 PercP Cy5 (1:50 dilution) | BioLegend | 301848 |
| Anti-Human CD117 APC (1:50 dilution) | BD Biosciences | 550412 |
| Anti-Human CD34 PE-Cy7 (1:50 dilution) | BioLegend | 343516 |
| Anti-Human CD38 APC (1:50 dilution) | BioLegend | 303510 |
| Anti-Human CD123 PE-Cy7 (1:50 dilution) | BioLegend | 983702 |
| Anti-Human CD45RA BV421 (1:50 dilution) | BioLegend | 304130 |
| Anti-human CD11b APC (1:50 dilution) | BioLegend | 101212 |
| Anti-human CD11b FITC (1:20 dilution) | Immunotools | 21279113X2 |
| Annexin FITC (1:200) | Immunotools | 31490013X2 |
| Annexin APC (1:200) | Immunotools | 31490016X2 |
| CALR Polyclonal Antibody (FACS/CM) | ThermoFisher | PA3-900 |
| Donkey anti-Rabbit (H+L) AF647 | ThermoFisher | A32795 |
| Rabbit anti-Mouse (H+L) AF594 | ThermoFisher | A27027 |
| **Bacterial and virus strains** | | |
| **Biological samples** | | |
| Human AML blast cells | UMCG/REC/UNICAMP | Ethical committee NL43844.042.13 |
| **Chemicals, peptides, and recombinant proteins** | | |
| 4′,6-diamidino-2-phenylindole | Sigma-Aldrich | 28718-90-3 |
| Paraformaldehyde | Sigma-Aldrich | 30525-89-4 |
| RNAse |  |  |
| DNase I | Roche | 11284932001 |
| MgSO4 | Sigma-Aldrich | M7506 |
| Heparin | Sigma-Aldrich | 60800-63-7 |
| Verapamil hydrochloride | Sigma-Aldrich | 152-11-4 |
| Cytarabine | Sigma-Aldrich | 147-94-4 |
| Arsenic Trioxide | Sigma-Aldrich | 1327-53-3 |
| *All Trans* Retinoic Acid | Sigma-Aldrich | 302-79-4 |
| Midostaurin | Sigma-Aldrich | M1323 |
| Quizartinib | Selleckchem | S1526 |
| Venetoclax | Selleckchem | S8048 |
| Rotenone | Sigma-Aldrich | 83-79-4 |
| Antimycin A | Sigma-Aldrich | 1397-94-0 |
| Metformin | Sigma-Aldrich | 317240 |
| Oligomycin A | Sigma-Aldrich | 579-13-5 |
| 2,2-dichloroacetophenone (DAP) | Sigma-Aldrich | D54850 |
| 2-deoxy-D-glucose (2-DG) | Sigma-Aldrich | 154-17-6 |
| KPT-9274 (ATG-019) | Selleckchem | S8444 |
| (E)-Daporinad (FK866) | MedChemExpress | HY-50876 |
| Human Interleukin 6 | Peprotech | 200-06 |
| Human Interleukin 3 | Peprotech | 200-03 |
| Human Granulocyte colony-stimulating factor | Peprotech | 300-23 |
| Human Thrombopoietin | Amgen |  |
| β-mercaptoethanol | Merck Sharp & Dohme BV | 60-24-2 |
| SsoAdvanced Universal SYBR® Green Supermix | BioRad | 1725274 |
| iScript cDNA synthesis Kit | BioRad | 1708891BUN |
| Tetramethylrhodamine, Ethyl Ester, Perchlorate | Thermofisher | T669 |
| MitoTracker DeepRedTM | Thermofisher | M22426 |
| MitoTracker GreenTM | Thermofisher | M7514 |
| RetroNectin® Pro (Recombinant Human Fibronectin Fragment) | Takara Bio | T101A |
| **Critical commercial assays** | | |
| Seahorse XFe96 Flux Analyzer | Agilent |  |
| **Deposited data** | | |
| Label Free proteome on primary AML blasts (CD34^+^) | De Boer et al., 2018 | PXD030463 |
| **Experimental models: Cell lines** | | |
| MOLM13 | DSMZ | ACC 554 |
| MV4-11 | ATCC | CRL-9591™ |
| HL60 | ATCC | CCL-240™ |
| OCI-AML3 | DSMZ | ACC 582 |
| NB4 | Harvard Medical School | Prof. Pier Paolo Pandolfi |
| NB4-R2 | Harvard Medical School | Prof. Pier Paolo Pandolfi |
| MS-5 | DSMZ | ACC 441 |
| Lenti-X 293T^TM^ | Takara | CRL-3216 |
| MOLM14 | UMCG | Dr. Emmanuel F Griessinger |
| ASE2 | Nagasaki University School of Medicine | Dr. M. Tomonaga |
| KBM7 | Brummelkamp lab | Dr. Thijn Brummelkamp |
| Kasumi-1 | DSMZ | ACC 220 |
| HEL | DSMZ | ACC 11 |
| KG1 | DSMZ | ACC 14 |
| TF1 | DSMZ | ACC 334 |
| U937 | DSMZ | ACC 5 |
| THP1 | ATCC | TIB-202™ |
| K562 | ATCC | CCL-243 |
| OCI-AML2 | DSMZ | ACC 99 |
| SET2 | DSMZ | ACC 608 |
| IMS-M2 | Cellosaurus | CVCL_RL93 |
| **Experimental models: Organisms/strains** | | |
| NOD.Cg-Prkdcscid Il2rgtm1Wjl/SzJ | The Jackon Laboratory | RRID:IMSR_JAX:005557 |
| **Oligonucleotides** | | |
| *CYTB* Forward primer | Eurofins | CACGATTCTTTACCTTTCACTTCATC |
| *CYTB* Reverse primer | Eurofins | TGATCCCGTTTCGTGCAAG |
| *HPRT1* Forward primer | Eurofins | GAACGTCTTGCTCGAGATGTGA |
| *HPRT1* Reverse primer | Eurofins | TCCAGCAGGTCAGCAAAGAAT |
| *ACTB* Forward primer | Eurofins | AGGCCAACCGCAAGAAG |
| *ACTB* Reverse primer | Eurofins | ACAGCCTGGATAGCAACGTACA |
| *RPL30* Forward primer | Eurofins | ACTGCCCAGCTTTGAGGAAAT |
| *RPL30* Reverse primer | Eurofins | TGCCACTGTAGTGATGGACAC |
| *PKLR* Forward primer | Eurofins | AGCCCAAATGGCCTTGAA |
| *PKLR* Reverse primer | Eurofins | AGAGACAGAATGCCAGTGAGC |
| *HBB* Forward primer | Eurofins | ACCTCAAGGGCACCTTTGC |
| *HBB* Reverse primer | Eurofins | AAAACATCAAGCGTCCCATAGAC |
| *BCL2* Forward primer | Eurofins | TGTGGATGACTGAGTACCTGA |
| *BCL2* Reverse primer | Eurofins | ACAAAGGCATCCCAGCCT |
| *POLG* Forward primer | Eurofins | GCTGCCTCACAAGGATGGTA |
| *POLG* Reverse primer | Eurofins | TTGATTTCCAGAGCACGGGG |
| **Recombinant DNA** | | |
| PLKO-Puro-GFP_shPOLG (plasmid) | Van den Boom et al., 2016 | GFP version of pLKO.1-Puro |
| **Software and algorithms** | | |
| FlowJo v10.0.6 | Treestar | http://www.flowjo.com/ |
| Prism 9 | GraphPad | http://www.graphpad.com/ |
| SPSS Statistical package 19.1 | IBM | https://www.ibm.com/ |
| Wave | Agilent | https://www.agilent.com/ |
| RStudio | CRAN | www.r-project.org |
| GSEA 4.0.1 | Broad Institute | https://software.broadinstitute.org/gsea/ |
| Cytoscape 3.4 |  | http://apps.cytoscape.org/apps/bingo |
| SynergyFinder |  | https://synergyfinder.aittokallio.group/20250412122454195361/\ |
| **Other** | | |
| FcR Blocking reagent, human | Miltenyi Biotec | 130-059-901  RRID: AB_2892112 |
| CD34 MicroBeads Kit UltraPure, Human | Miltenyi Biotec | 130-100-453 |
| CD117 MicroBeads Kit, Human | Miltenyi Biotec | 130-091-332 |
| CD3 MicroBeads, Human | Miltenyi Biotec | 130-050-101 |
| MethoCult™ | Stemcell | H4435 |
| FuGENE HD Transfection Reagent | Promega | E2312 |
| Amicon Ultra-15 Centrifugal Filter Unit – 100 KDa | Merck | UFC910024 |
| NAD/NADH Assay Kit (Colorimetric) | Abcam | Ab65348 |
| NucleoSpin® Tissue DNA extraction Kit | BioKE | 740952.250 |
| KAPA RNA HyperPrep Kit with RiboErase (HMR) | Roche | 08098131702 |
| BD CycletestTM Plus DNA Reagent Kit | Becton-Dickinson | 340242 |

Resource availability

**Lead contact**

Further information and requests for resources and reagents should be directed to and will be fulfilled by the lead contact, Jan Jacob Schuringa ([j.j.schuringa@umcg.nl](mailto:j.j.schuringa@umcg.nl)).

**Materials availability**

All the datasets presented in the paper are available in the supplemental material or deposited in the indicated repositories as described in the methods section. All RNA sequencing data supporting this study are available for download from the King ’s Open Research Data System (KORDS).

**Study approval and patient samples**

Overall, we enrolled 482 *de novo* AML samples in the Brazilian cohort (training cohort) and 105 *de novo* AML samples in the cohort of patients from the Netherlands (validation cohort). Out of the 482 patients included in the training cohort, 411 were treated with 3+7 based intensive chemotherapy and those were considered for clinical outcome analysis. Molecular data for *NPM1*, *IDH1*/2 and *FLT3*-ITD mutations was determined as previously described. ^1^ For the validation cohort, 36/105 patients were treated with 3+7 based protocols and 09/105 were treated with hypomethylating agents (decitabine and/or 5-Azacytidine). Mutational analysis was performed using target NGS panel including all the genes recommended by the European Leukemia-Net 2022 guideline. ^2^ Neonatal cord blood (CB) was obtained from healthy full-term pregnancies from the Obstetrics departments of the University Medical Center and Martini Hospital in Groningen, The Netherlands, after informed consent. The protocol was approved by the Medical Ethical Committee of the UMCG. Donors are informed about procedures and studies performed with CB by an information sheet that is read and signed by the donor, in line with regulations of the Medical Ethical Committee of the UMCG (protocol #NL43844.042.13). Peripheral blood mononuclear cell derived CD34^+^ stem cells (PBMSCs) and CB derived CD34^+^ cells were isolated by density gradient separation, followed by a hematopoietic progenitor magnetic associated cell sorting kit from Miltenyi Biotech (#130-046-702) according to the manufacturer’s instructions (purity superior to 90%, by flow cytometry). All CD34^+^ healthy cells were pre-stimulated for 24-48hrs prior to experimental use for *ex vivo* assays (or otherwise directly stored in cell lysis buffer for DNA extraction, to evaluate the mtDNAc). CB derived cells were pre-stimulated with Stemline II hematopoietic medium (SigmaAldrich; #S0192), 1% penicillin/streptomycin (PS) supplemented with SCF (255-SC, Novus Biologicals), FLT3-Ligand (Amgen) and N-plate (TPO) (Amgen) (all 100 ng/ml). PBMSC CD34^+^ cells were pre-stimulated with Stemline II, 1% PS, 20% FCS along with SCF, FLT3-Ligand, N-plate (all 100 ng/ml) and IL-3 (Sandoz) and IL-6 (both 20 ng/ml). Primary AMLs were grown on MS5 stromal cells with G-CSF (Amgen), N-Plate and IL-3, all 20 ng/ml.

**Transcriptomic and metabolomic analysis in AML cell lines**

The RNA sequencing and metabolomic analysis were performed on 13 AML cell lines at the Broad Institute included into the Cancer Cell Line Encyclopedia datasets. ^3,4^ Data were retrieved via the cBioPortal platform. ^5^

**METHOD DETAILS**

**Flow cytometry**

Cryopreserved MNC fractions of AML patients were thawed, resuspended in newborn calf serum (NCS) supplemented with DNase I (20 Units/mL), 4 μM MgSO_4_ and heparin (5 Units/mL) and incubated at 37°C for 15 minutes (min). To analyze the hematopoietic stem progenitor cell (HSPC) populations of the AML bulk samples, 5x10^5^ mononuclear cells were blocked with human FcR blocking reagent (Miltenyi Biotec) for 5 min and stained with the following antibodies: CD45-FITC, CD34-PE, CD38-ACP, CD123-Pe-Cy7, CD45RA-BV421, and viability marker 7-AAD for 20 min at 4°C. The different HSPC populations were identified based on their CD-marker expression as follows: L-HSC: CD45^dim^CD34^+^CD38^-^CD45RA^-^; L-LMPP: CD45^dim^CD34^+^CD38^-^CD45RA^+^; L-GMP: CD45^dim^CD34^+^CD38^+^CD45RA^+^CD123^+^; L-CMP: CD45^dim^CD34^+^CD38^+^CD45RA^-^CD123^+^; L-MEP: CD45^dim^CD34^+^CD38^+^CD45RA^-^CD123^-^. Additionally, we analyzed the total mitochondrial mass (measured by MitoTracker Green^TM^) and mitochondrial membrane potential (measured by the Tetramethylrhodamine, ethyl ester; TMRE) and the surface Calreticulin (CALR) expression. All staining procedures were performed in the presence of verapamil hydrochloride (12.5 µM) to avoid efflux of the mitochondrial related probes. Fluorescence was measured on the BD LSRII or FACS Symphony A5 and analyzed using Flow Jo (Tree Star, Inc). For each sample a minimum of 20000 events were acquired inside the SSC-A^low^CD45^dim^7-AAD^-^ population.

**Cell cycle analysis**

Cell cycle phases were determined by BD CycletestTM Plus DNA Reagent Kit (Becton-Dickinson) according to the manufacturer’s instructions. A total of 4 × 10^5^ AML cells were seeded in 24-well plates, treated with metformin (1 mM) and vehicle control and collected and fixed at distinct timepoints: 24, 48 and 72 h. DNA content distribution was acquired with the BD LSRFortessa^TM^ cytometer (Becton-Dickinson) and analyzed with the FlowJo software (Treestar, Inc., USA).

**Clinical endpoint and statistical analysis**

Survival analyzes were performed in AML patients treated with intensive chemotherapy (3+7 scheme) as an induction protocol. ^1^ For the Brazilian cohort (training cohort), we first scored the mtDNA content (mtDNAc) of all AML samples and compared them with the healthy donors (CD34^+^ and PBMCs). Next, we used the 95^th^ percentile for the mtDNAc of the healthy donor cohort to dichotomize the AML patients into normal mtDNAc and high mtDNAc groups. The same strategy was then used for the validation cohort. Overall survival (OS) was defined as the time from diagnosis to death from any cause related to the disease, those alive or lost to follow-up were censored at the date last known alive. For patients who achieved complete remission (CR), disease-free survival (DFS) was defined as the time from CR achievement to the first adverse event: relapse, development of secondary malignancy, or death from any cause, whichever occurred first. Univariate and multivariate proportional hazards regression analysis was performed for potential prognostic factors for OS. Potential prognostic factors examined and included in multivariable regression analysis were Adapted Genetic Risk (AGR) stratification^6^ (for the training cohort), ELN2022 risk stratification^2^ (for the validation cohort), age at diagnosis (analyzed as continuous variable), gender, and the mtDNAc (as a continuous and categorical variable). The proportional hazards (PH) assumption for each continuous variable of interest was tested. Linearity assumption for all continuous variables was examined in logistic and PH models using restricted cubic spline estimates of the relationship between the continuous variable and log relative hazard/risk. Descriptive analyses were performed for patient baseline features. Fisher’s exact test or Chi-square test, as appropriate, was used to compare categorical variables. Mann-Whitney or Kruskal-Wallis test was used to compare continuous variables. Details of the statistical analysis and clinical endpoints were described elsewhere. All P values were two sided with a significance level of 0.05. All statistical analyses were performed using the statistical package for the social sciences (SPSS) 19.0 and R 3.3.2 (The CRAN project, www.r-project.org) software.

**Real Time quantitative polymerase chain reaction (qPCR) for determination of mtDNA content (mtDNAc) and gene expression analysis**

Real Time quantitative PCR assays were performed in triplicate using sample-derived DNA on CFX384 Touch Real-Time PCR Detection System (Bio-Rad). The reaction solution was prepared by combining the SsoAdvanced SYBR Green Supermix (Bio-Rad) and 320 nM each of primers. Negative controls without template were run for each gene. At the end of the amplification process, the amplification specificity of the gene was assessed by a melting curve between 55 and 95 °C. The corresponding real-time PCR efficiencies for each mitochondrial and nuclear gene amplification were calculated according to the equation: E = 10^(-1/slope)^ -1. The efficiency of *CYTB*, *PKLR* and *HBB* genes was higher than 97%.

The relative mtDNAc was defined as the total amount of mtDNA divided by the total amount of nuclear DNA (mtDNA amount/nDNA amount). Next, the ratio for each sample was normalized to a reference DNA (a genomic DNA sample from a healthy control subject) and then defined as the measurement of relative mtDNAc. Importantly, the same reference DNA was used as an internal control in all experiments to ensure that the results of different experiments could be comparable. Following standardization between different runs, the relative mtDNAc was obtained using the comparative cycle threshold (∆Ct) method, and the results were expressed using 2^-ΔΔCt^, in which ΔΔCt = ΔCt ^patients^ – ΔCt ^healthy volunteer^.

For the *BCL2* and *POLG* gene expression analysis by qPCR, RNA was reverse transcribed using the iScript cDNA synthesis kit (Bio-Rad) and amplified using SsoAdvanced SYBR Green Supermix (Bio-Rad) on a CFX384 Touch Real-Time PCR Detection System (Bio-Rad). The *ACTB*, *HPRT1* and *RPL30* were used as housekeeping genes. Primer sequences are listed in the key resources table.

**Lentiviral vectors and lentivirus production**

*POLG* knockdown was performed in patients from both the training and validation cohorts. For the training cohort, a single pLKO-based construct was designed, incorporating five independent shRNA sequences targeting the *POLG* gene (sequences sourced from Sigma-Aldrich and Dharmacon reagents, used in experiments shown in Fig. 2E-F). In the validation cohort, two distinct *POLG*-targeting sequences were individually cloned into separate shPOLG constructs (shPOLG#1 – target sequence: GCGCTTACTAATGCAGTTTAA and shPOLG#2 – target sequence: GAGATCGCTGGGCCTCTTC, obtained from Sigma-Aldrich and Dharmacon reagents). For all experiments, a pLKO-PURO-GFP backbone plasmid was used, and lentivirus was produced using various lentiviral backbone plasmids, as detailed in the resource table. Lentivirus generation was performed in Lenti-X™ 293T cells following the standard three-plasmid packaging procedure. ^7^ Lentiviral particles were concentrated using Amicon Ultra-15 centrifugal filter unit columns (Merck, CA, USA). Cells were sorted based on their GFP protein expression and posteriorly used for *in vitro* assays. The efficiency of infection was further confirmed by mtDNAc quantification. A shRNA sequence that does not target human genes (referred to as scrambled) was used as a control.

***In vitro* primary AML cell proliferation upon *POLG*-knockdown**

Cryopreserved MNC fractions of AML patients were thawed as described in the section “Flow cytometry”. CD34^+^ cells were isolated from primary AML patients on the autoMACS using a magnetically activated cell-sorting progenitor kit (Miltenyi Biotech). In case of *NPM1* mutated AMLs with CD34 expression <1%, the CD117^+^ blast cells were isolated.

A total of 1x10^5^-2.5x10^5^ primary AML were twice lentivirally transduced using retronectin coated plates (Takara Bio Europe SAS), with short hairpin RNA targeting the *POLG* gene (shPOLG#1 and shPOLG#2) and with a shRNA sequence that does not target human genes as a control (shCTRL). Two days after the second transduction, GFP positive cells were sorted by flow cytometry and expanded in culture for 21 days. The co-cultures were performed in Gartner’s medium consisting of IMDM (Thermo Scientific) supplemented with 20% fetal calf serum (Gibco), 1% penicillin and streptomycin, 2 mM glutamine (Gibco), 57.2 mM β-mercaptoethanol (Merck Sharp & Dohme BV), and 20 ng/mL G-CSF, N-plate (TPO), and IL-3. Co-cultures were grown at 37°C and 5% CO_2_ and demi-populated after counting if necessary. Cell proliferation was assessed with a hemocytometer until 21 days of co-culture and cross validated by counting the viable cell population (DAPI^-^ cells), which were CD45^dim^ by flow cytometry evaluation using the NovoCyte Quanteon System (Agilent, CA, USA).

**Functional assays in healthy cord blood (CB)-derived CD34^+^ cells**

PBMCs were isolated by a density gradient using Ficoll (Sigma-Aldrich) from CB. MNCs were washed once at 450g with PBS-EDTA (5 mM) and resuspended in 300 μL of PBS. Next, 100 μL of FcR blocking reagent and 100 μL of CD34 MicroBeads (Miltenyi Biotech) were added to the suspension and incubated for 30 min at 4°C. After incubation cells were washed for 10 min at 450g and resuspended in 2 mL of PBS–EDTA (5 mM). Cells were passed through a cell strainer (70 µm) and isolated by magnetic separation on the autoMACS (Program – Possedels, Miltenyi Biotech). The purity of the isolated cells was routinely evaluated by FACS and in the range of 85% to 95%.

CB isolated CD34^+^ were next expanded in Stemcell II medium supplemented with 100 ng/ml SCF, 50 ng/ml FLT3-Ligand, 30 ng/ml GM-CSF and 10 ng/ml IL-6. For colony formation assays, a total of 300 CB CD34^+^ cells were seeded on methylcellulose (H4230, Stem Cell Technologies, Vancouver, Canada) supplemented with SCF, FLT3-Ligand, N-plate (all 100 ng/mL), and EPO, IL-3, and IL-6 (all 20 ng/mL) in the presence of vehicle or metformin (1 mM), DAP (2 µM), 2-DG (2 mM), KPT-9274 (1.5 µM) and the combinations. After 8 days for CFU-E/BFU-E and 14 days for CFU-G/GM, colonies were identified and counted. All cell cultures were grown at 37°C and 5% CO_2_.

**Oxygen consumption (OCR) and extracellular acidification rate (ECAR) measurements**

Oxygen consumption rate (OCR) and Extra Cellular Acidification Rate (ECAR) were measured using Seahorse XF96 analyzer (Seahorse Bioscience, Agilent, US) at 37 °C. Treated AML cell lines and sorted CD34^+^ or CD117^+^ from primary AML patients (drugs and time-points indicated in the plots), 1x10^5^ and 2x10^5^ viable cells (DAPI^-^) were seeded per well in poly-L-lysine (Sigma-Aldrich) coated Seahorse XF96 plates in 180 μL XF Assay Medium (Modified DMEM, Seahorse Bioscience), respectively. For OCR measurements, XF Assay Medium was supplemented with 10 mM Glucose and 2.5 µM oligomycin A (Port A), 2.5 µM FCCP (carbonyl cyanide-4-(trifluorometh oxy) phenylhydrazone) (Port B) and 2 µM antimycin A together with 2 µM Rotenone (Port C) were sequentially injected in 20 µL volume to measure basal and maximal OCR levels (all reagents from Sigma-Aldrich). For ECAR measurements, Glucose-free XF Assay medium was added to the cells and 10 mM Glucose (Port A), 2.5 µM oligomycin A (Port B) and 100 mM 2-deoxy-D-glucose (Port C) (all reagents from Sigma-Aldrich). In parallel, AML cells were evaluated with the mitochondrial markers – MitoTracker Green^TM^, MitoTracker DeepRed and Tetramethylrhodamine, Ethyl Ester, Perchlorate (TMRE; ThermoFisher) by flow cytometry (LSRII or Symphony A5). For measuring the metabolic consequences on treated AML cells upon VEN (1 µM), KPT-9274 (1.5 µM), and metformin (1 mM), cells were plated directly on Seahorse plates (1x10^5^ cells per well, in 4 technical replicates) and treated with the inhibitors for 48 hours prior the assay. All XF96 protocols consisted of 4 times mix (2 min) and measurement (2 min) cycles, allowing for determination of OCR at basal and in between injections. For the assays involving metformin injections, metformin was added as the first injection of the assay (final concentration – 1 and 5 mM) and OCR and ECAR were measured over time for 8 cycles. Both basal and maximal OCR levels were calculated by assessing metabolic response of the cells in accordance with the manufacturer’s suggestions. The OCR measurements were normalized to the viable number of cells used for the assay.

**TF1 RNA-sequencing experiments**

RNA samples for sequencing were prepared for TF1 cells treated with metformin (5 mM) or vehicle (DMSO) for 48 hours. Cells were collected after and remaining viable cells were isolated for posterior RNA extraction. Total RNA was isolated using the RNeasy Mini Kit from Qiagen (Venlo, The Netherlands) according to the manufacturer’s recommendations. The obtained cDNA fragment libraries were sequenced on an Illumina NextSeq500 using default parameters (25M reads per sample). Sequencing reads were mapped to hg38 with STAR version 2.7.3a ^8^ using the default parameters filtered for uniquely mapping reads with the following modifications: ‘--outFilterType BySJout --outFilterMultimapNmax 20 --outFilterMismatchNoverLmax 0.04 --outSAMtype BAM sorted --outSJfilterReads Unique --chimSegmentMin 20’. Read counts were normalized as counts per million (CPM) and log2 transformed (Log2CPM). We used a filtering approach to eliminate non-expressed or marginally expressed genes from the 60,649 genes defined in ENSEMBL annotation. We retained genes that had a CPM > 8 in at least half of the samples of at least one of the experimental conditions considered. Thus, we retained 10,326 genes in our analysis.

We generated gene expression profiles by computing differential expressed genes (DEG), computing the log2-fold changes (Log2FC), p-values of differential expression (Wilcoxon), and the false discovery rate (FDR)–adjusted p-values (Benjamini and Hochberg) of DEG in all the profiles. The statistical significance was set as FDR < 0.01. Differentially expressed genes were clustered using unsupervised hierarchical clustering with Euclidean distances (complete) (stats package in R). Data is available for download from the King ’s Open Research Data System (KORDS).

**Gene ontology (GO) and gene set enrichment analyzes (GSEA)**

Gene set enrichment analysis (GSEA) was performed using the Broad Institute software (http://software.broadinstitute.org/gsea/index.jsp). Gene ontology (GO) was evaluated using the gene ontology resource (http://geneontology.org/) and the BinGO plugin using the Cytoscape software v3.8.2 (NIGMS, USA). For the proteomic datasets (as described in Supplementary fig. 1f), protein expression was correlated with the mtDNAc values, and ranked lists based on the Pearson correlation values were used to perform the GSEA analysis. Using single-sample GSEA (ssGSEA), we generated enrichment scores (ES) for all the other 35k signatures present in the MSigDB ^9^ and used the ES values to correlate the genetic signatures with the mtDNAc levels (in Fig. 1h). All genes from the RNA-seq of the different experimental groups (metformin treated *versus* vehicle control) cohort were pre-ranked according to their differential expression (fold change). Enrichment scores (ES) were obtained with the Kolmogorov-Smirnov statistic, tested for significance using 1000 permutations, and normalized (NES) to consider the size of each gene set. As suggested by the GSEA, a false discovery rate (FDR) cut-off of 25% (FDR q-value < 0.25) was used. ^9^ Data visualization was performed with the ClustVis platform. ^10^

**Enzymatic activity assays**

Both extracellular lactate and glucose concentrations were determined from the cell culture medium by monitoring NAD(P)H increase occurring during specific enzymatic reactions for each metabolite at 340 nm wavelength, as described elsewhere. ^11–13^ Briefly, extracellular lactate concentrations were determined by the lactate dehydrogenase (LDH) enzymatic reaction in the cell culture medium taken at 0-hour post-treatment of AML cells with metformin (5 mM) or vehicle control, for 48 hours. For baseline measurements, AML cells (cell lines and primary AML cells) were seeded in liquid culture and culture medium was taken after 24 hours. Extracellular lactate was converted by L-Lactic Dehydrogenase (LDH, Sigma Aldrich) reaction in freshly prepared 25 mM NAD^+^ and 87.7 U/mL LDH in 0.4 M hydrazine (Sigma Aldrich)/0.5M glycine assay buffer (pH 9). 20 µl samples (diluted according to standard curve) and sodium L-lactate (Sigma Aldrich) standards were pipetted into 130 µL reagent mix in 96 wells plate format and the reaction was carried out for 30 min at 37 °C. Glucose consumption was detected using an enzymatic reagent mix consisting of 75 μL 100 mM PIPES buffer, 2.5 μL 40 mM NADP, 2 μL 10 mM ATP, 1 μL 500 mM MgSO_4_, 0.15 μL hexokinase, 0.15 μL glucose-6-phosphate-dehyrogenase, and 44.3 μL H20 per well was prepared and 125 μL added to both medium and glucose standard wells. The plate was incubated for 30 min at 37 °C. Values of consumption and release of extracellular metabolites for each sample were normalized by cell number and incubation time considering the exponential growth curve. The NAD/NADH quantification colorimetric kit (abcam, CB, UK) was used to quantify total NAD levels in OCI-AML3 cells treated with metformin (5 mM), KPT-9274 (150 nM) and daporinad (100 nM) for 48 hours prior to the measurements. Assay was performed as previously described. ^14^ Briefly, cells were washed twice in PBS, counted, and pelleted by centrifugation. Cell pellets were immediately used for analysis, following the manufacturer’s instructions. The total NAD level in each sample was measured and normalized based on the input cell number.

**References**

1. Bezerra, M. F. *et al.* Co-occurrence of DNMT3A, NPM1, FLT3 mutations identifies a subset of acute myeloid leukemia with adverse prognosis. *Blood* **135**, 870–875 (2020).

2. Döhner, H. *et al.* Diagnosis and management of AML in adults: 2022 recommendations from an international expert panel on behalf of the ELN. *Blood* **140**, 1345–1377 (2022).

3. Barretina, J. *et al.* The Cancer Cell Line Encyclopedia enables predictive modelling of anticancer drug sensitivity. *Nature* **483**, 603–7 (2012).

4. Nusinow, D. P. *et al.* Quantitative Proteomics of the Cancer Cell Line Encyclopedia. *Cell* **180**, 387-402.e16 (2020).

5. Gao, J. *et al.* Integrative analysis of complex cancer genomics and clinical profiles using the cBioPortal. *Sci. Signal.* **6**, pl1 (2013).

6. Silveira, D. R. A. *et al.* Integrating clinical features with genetic factors enhances survival prediction for adults with acute myeloid leukemia. *Blood Adv.* **4**, 2339–2350 (2020).

7. Pereira-Martins, D. A. *et al.* MLL5 improves ATRA driven differentiation and promotes xenotransplant engraftment in acute promyelocytic leukemia model. *Cell Death Dis.* **12**, (2021).

8. Dobin, A. *et al.* STAR: ultrafast universal RNA-seq aligner. *Bioinformatics* **29**, 15–21 (2013).

9. Subramanian, A. *et al.* Gene set enrichment analysis: A knowledge-based approach for interpreting genome-wide expression profiles. *Proc. Natl. Acad. Sci. U. S. A.* **102**, 15545–15550 (2005).

10. Metsalu, T. & Vilo, J. ClustVis: a web tool for visualizing clustering of multivariate data using Principal Component Analysis and heatmap. *Nucleic Acids Res.* **43**, W566-70 (2015).

11. Erdem, A. *et al.* Inhibition of the succinyl dehydrogenase complex in acute myeloid leukemia leads to a lactate-fuelled respiratory metabolic vulnerability. *Nat. Commun.* **13**, 2013 (2022).

12. Weinhäuser, I. *et al.* M2 macrophages drive leukemic transformation by imposing resistance to phagocytosis and improving mitochondrial metabolism. *Sci. Adv.* **9**, eadf8522 (2023).

13. Weinhäuser, I. *et al.* Thiostrepton induces cell death of acute myeloid leukemia blasts and the associated macrophage population. *Haematologica* **109**, 639–645 (2024).

14. Subedi, A. *et al.* Nicotinamide phosphoribosyltransferase inhibitors selectively induce apoptosis of AML stem cells by disrupting lipid homeostasis. *Cell Stem Cell* **28**, 1851-1867.e8 (2021).

**
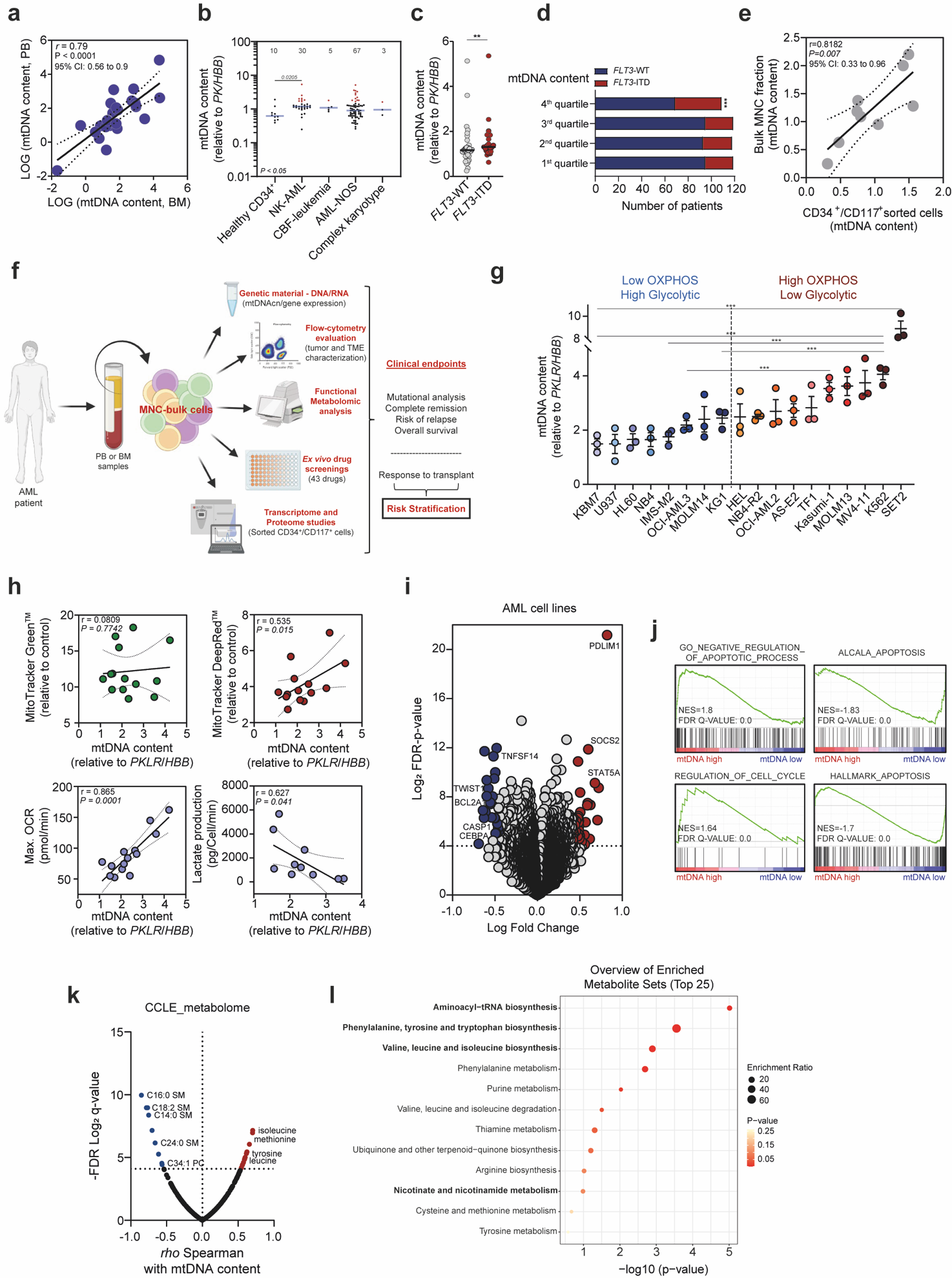
**

**Figure. S1.**

**High mitochondrial DNA content (mtDNAc) is associated with *FLT3*-ITD mutations. AML cell lines faithfully recapitulate the metabolic heterogeneity observed in primary AML samples.** (a) Correlation of mtDNAc of 25 paired AML samples from which BM and PB samples were available at the time of diagnosis. Logarithmic values of the mtDNA content from BM and PB were used in the correlations analyses to better fit the data. (b) The relative quantification of mtDNAc relative to a single-copy nuclear gene (*PKLR* and *HBB*) in the validation cohort (n=105 AML samples and n=10 healthy CD34^+^). The horizontal bars represent the median value of mtDNAc. Groups were compared using Kruskal-Wallis H test with Dunn`s multiple comparison test. Dot plot shows the distribution of mtDNAc in AML samples from the validation cohort (c) and training cohort (d) according to the *FLT3*-ITD mutational status. WT, wild type (validation cohort, n=32); ITD, in tandem duplication (validation cohort, n=23). Groups were compared using Mann–Whitney U test when comparing two groups or Kruskal-Wallis H test with Dunn`s multiple comparison test when comparing more than two groups. (e) Correlation between mtDNAc in paired bulk MNC and sorted CD34^+^/CD117^+^ (for CD34^-^ cells) in AML patients included in the validation cohort (n=9). (f) Schematic workflow representation of the functional *ex vivo* evaluation of AML samples included in the validation cohort. Details regarding the included measurements and statistical analysis performed can be found in the supplementary methods section. Created with BioRender.com. (g) The quantification of mtDNAc relative to a single-copy nuclear gene (*PKLR* and *HBB*) in a panel of AML cell lines (n=18 lines). Cell lines were compared using Mixed-effect analysis with Dunn`s multiple comparison test. Cell lines are described and indicated in the graph. Spearman correlations between the mtDNAc and the (h) mitochondrial mass (measured with the MitoTracker Green^TM^ probe, upper left panel) and mitochondrial membrane potential (measured with MitoTracker DeepRed^TM^ probe, upper right panel), maximum oxygen consumption rate (OCR, lower left panel), and lactate secretion (lower right panel) in a panel of AML cell lines (n=14 lines, except for lactate production – n=10). (i) Volcano plot displaying the differentially expressed genes in AML cell lines with high mtDNAc versus normal mtDNAc using the RNA-seq data from AML cell lines (CCLE dataset). (j) GSEA analysis using the Pearson correlations depicted in (i). NES and FDR-q are indicated. (k) Spearman correlation of mtDNAc and endo metabolites obtained AML cell lines (CCLE dataset). Blue and red dots indicate significant negative and positive correlations, respectively. (l) Dot plots showing the metabolite pathway analysis on significantly associated metabolites with mtDNAc in the AML cell lines, as determined by Metaboanalyst software analysis. P-value represents adjusted P value after multiple comparison Benjamini–Hochberg test. The number of biological replicates is indicated by the dots on the plots. Each biological replicate is an average of at least two independent technical replicates.


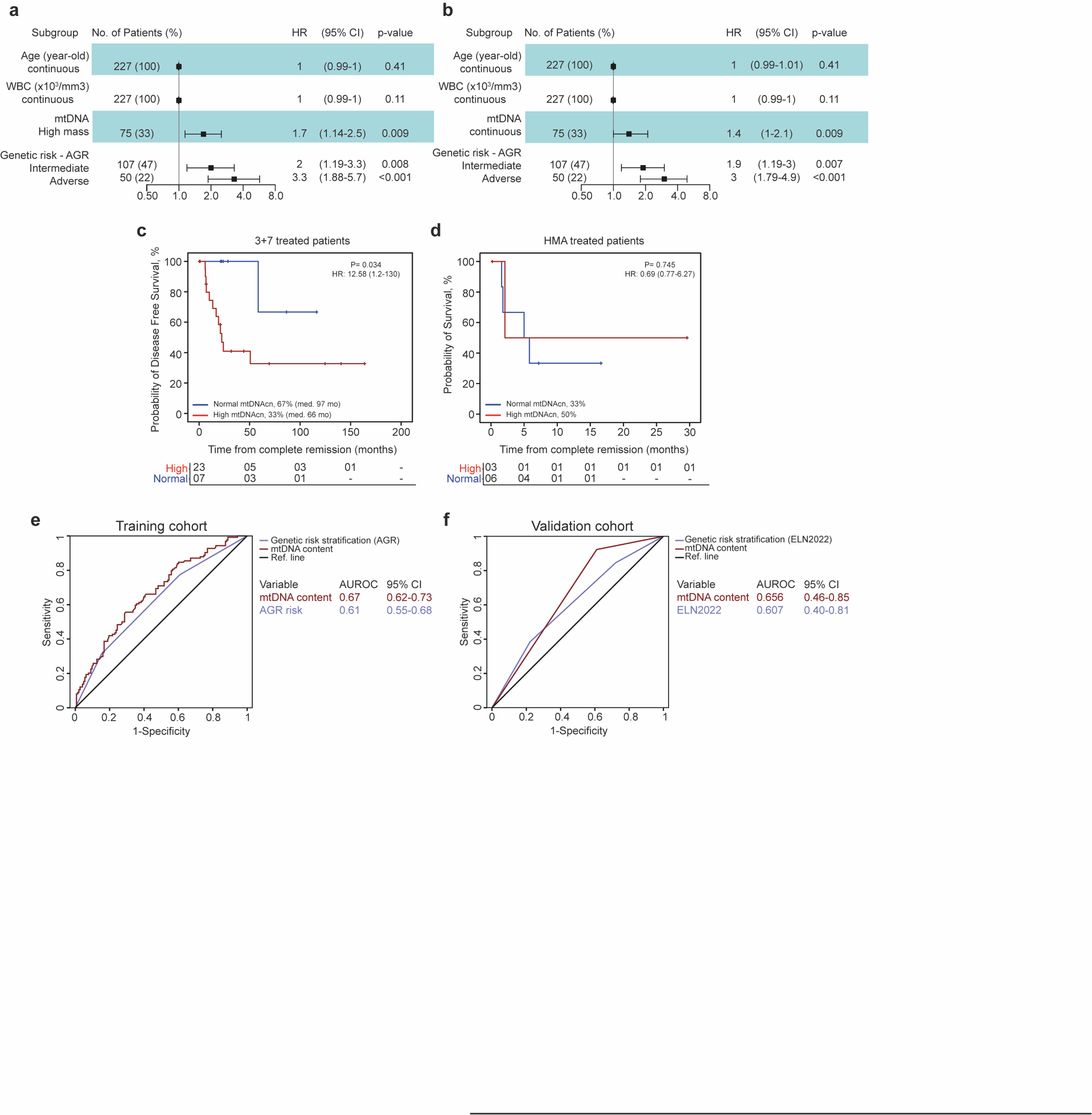


Figure. S2.

**mtDNAc predicts responses to intensive chemotherapy but not hypomethylating agents in AML.** Forest plot representing the multivariate Cox proportional hazard model for the DFS considering the mtDNAc as a categorical (a) and continuous parameter (b) in AML patients according to the mtDNAc. Confounding variables included in the model were mtDNAc (high mass versus normal mass), AGR genetic risk stratification (Favorable versus Intermediate versus Adverse), age and white blood cells counts (continuous variables). (c) The probability of disease-free survival, DFS in AML patients (validation cohort) treated with 3+7 based protocols (c) or with hypomethylating agents (HMAs, Decitabine or 5-Azacytidine) (d) according to the mtDNAc (validation cohort). DFS curves were estimated using the Kaplan–Meier method, and the log‐rank test was used for comparison. Panels (e) and (f) display the area under the curve (AUROC) values assessing the predictive ability of mtDNAc (red curve) and genetic risk stratification (blue curve) for DFS in the training and validation cohorts, respectively. Genetic risk was determined using AGR for the training cohort and ELN2022 for the validation cohort. CI = confidence interval. AUROC = 1.0 indicates perfect prediction, while AUROC = 0.5 suggests no predictive ability.


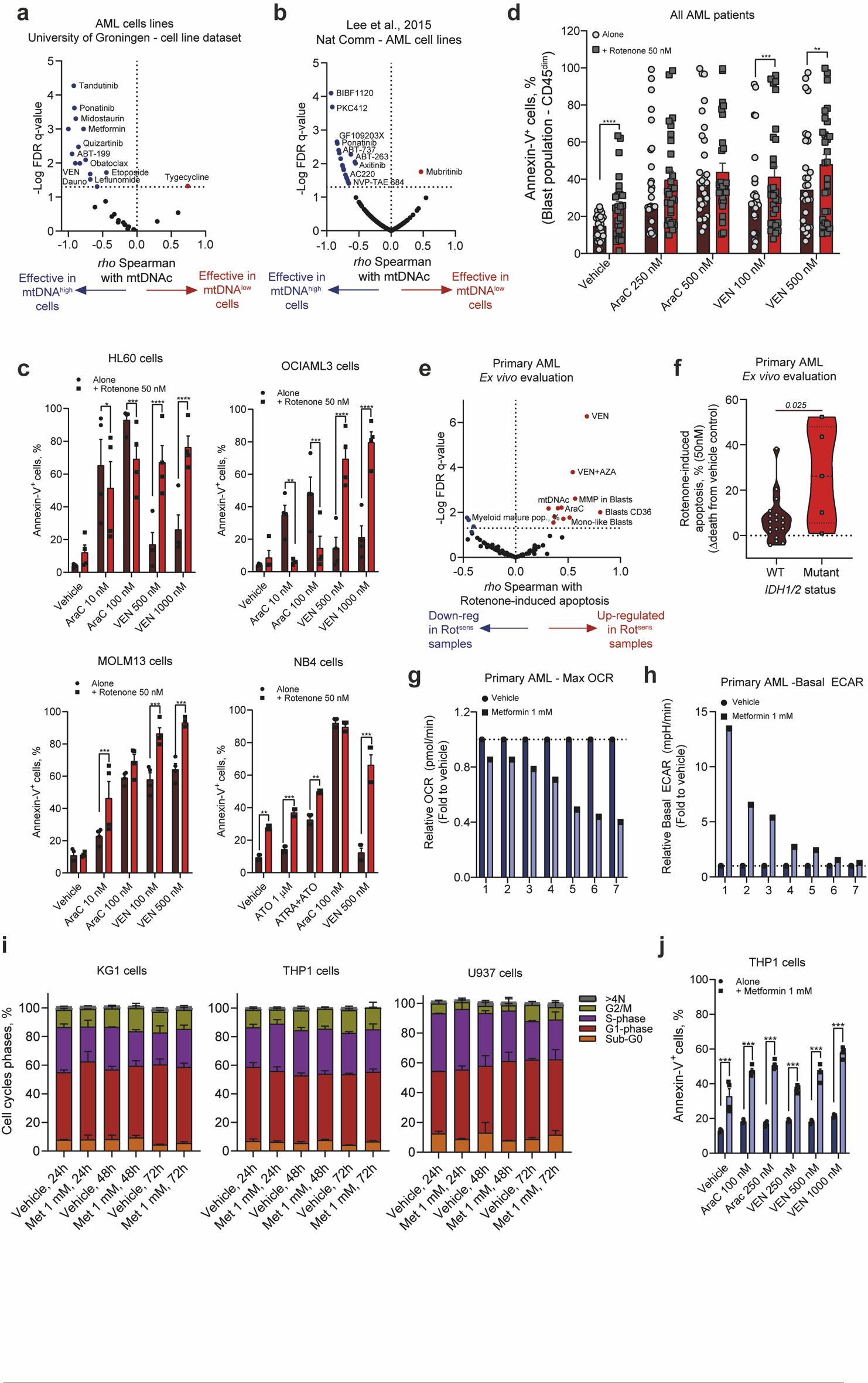

Figure. S3.

**AML cells with high mtDNAc display increased sensitivity to mitotoxic drugs (mitocans) and topoisomerase II inhibitors.** Spearman correlations between the mtDNAc and the drug-response (computed as area under the curve values) of drugs targeting the mitochondrial metabolism (mitocans,), topoisomerase II, the pyrimidine pathway and the purine pathway in a panel of AML cell lines (n=10 lines) extracted from the Depmap portal (a) and Lee et al., 2015 dataset (b). Negative correlations indicate sensitivity to the drug in relation to mtDNAc. Drug-induced apoptosis in HL60, OCIAML3, MOLM13, and NB4 (c) cells treated with AML-related drugs (AraC and VEN, concentrations indicated in the plots) in the presence or absence of rotenone (50 nM, 72 h) detected by flow cytometry using an APC-annexin V/DAPI staining method. (d) Apoptosis was detected by flow cytometry in gated human CD45^dim^ of *ex vivo* treated AML samples in a co-culture system using a FITC-annexin V/DAPI staining method. Cells were treated with vehicle, cytarabine (AraC, 250 and 500 nM), venetoclax (VEN, 100 and 500 nM) in the presence or absence of rotenone (50 nM) for 72 h. Bar graphs represent the mean ± SEM of all the independent patients screened, each point represents a patient. The p-values and cell lines are indicated in the graphs; *p < 0.05; **p < 0.01; ***p < 0.001, Groups were compared using Mixed-effect analysis with Dunn`s multiple comparison test. (e) Spearman correlations between the rotenone induced apoptosis and the functional readouts evaluated in primary AML cells treated *ex vivo*. (f) Violin plots depicting the rotenone induced apoptosis in primary AML cells dichotomized based on the *IDH1*/*2* mutational status. Comparison of maximum oxygen consumption rate (max. OCR) (g) and basal extracellular acidification rate (ECAR) (h) upon metformin treatment (1 mM, 48 hours) between primary AML samples treated with metformin (1 mM, 48 h) or vehicle control. Bar graphs represent normalized values expressed as a fold for each sample relative to vehicle-treated controls. Results are presented as the mean ± SEM of at least four technical measurements. (i) Cell cycle distribution was assessed by DNA content analysis using flow cytometry in KG1, THP1, and U937 cells following treatment with vehicle or metformin (1 mM) for 24, 48, and 72 hours. The bar graph represents the mean ± SD from at least three independent experiments. Cell cycle phases were compared using Mixed-effect analysis with Dunn`s multiple comparison test. (j) Drug-induced apoptosis in THP1 cells with AML-related drugs (VEN, venetoclax and AraC, cytarabine) in the presence or absence of metformin (1 mM, 72 h) detected by flow cytometry using an APC-annexin V/DAPI staining method. The number of biological replicates is indicated by the dots on the plots. Each biological replicate is an average of at least two independent technical replicates. Groups were compared using Mixed-effect analysis with Dunn`s multiple comparison test.


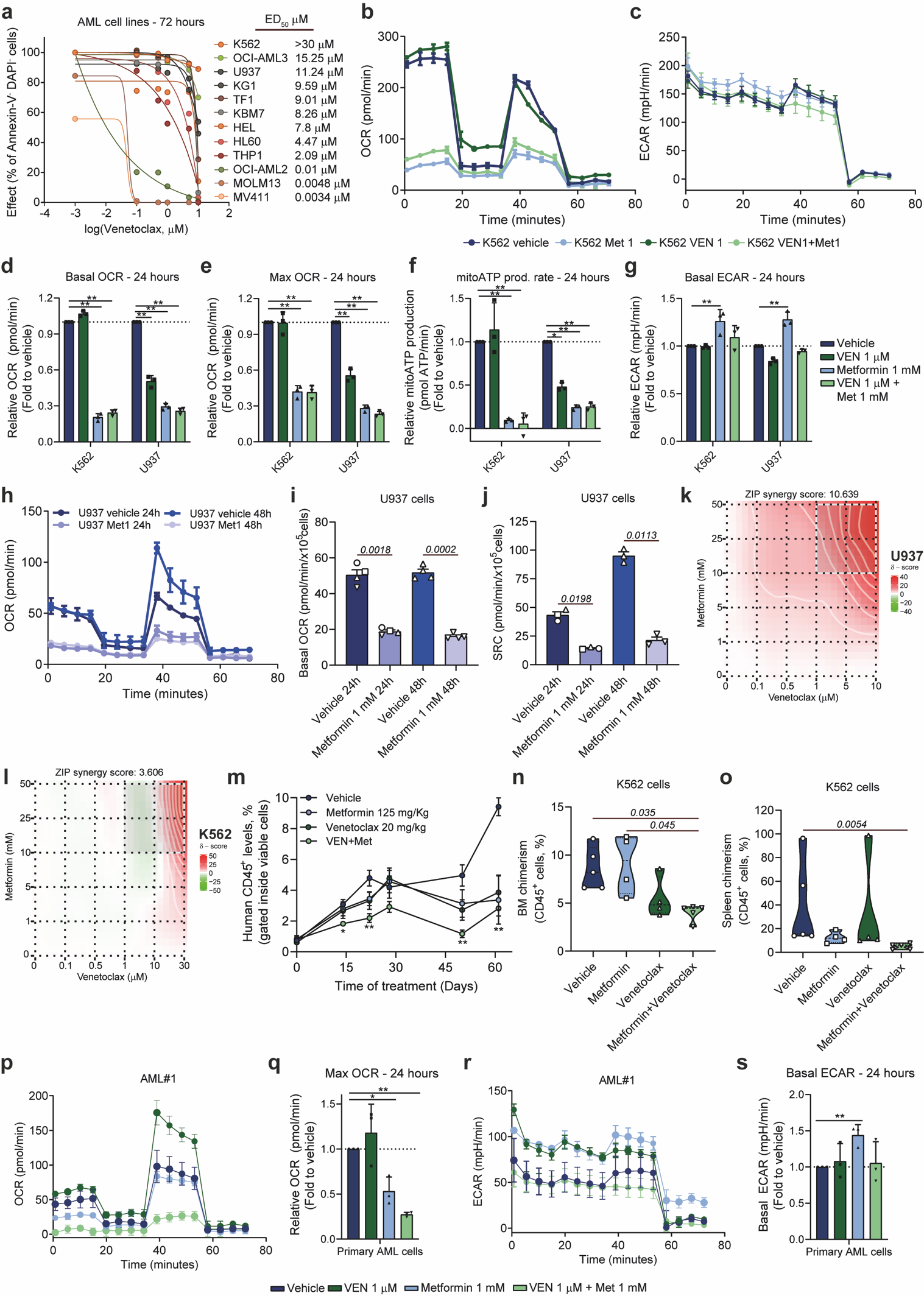


Figure. S4.

**Inhibition of mitochondrial respiration with metformin results in metabolic adaptation and increased glycolysis in AML cells.** (a) Dose-response cytotoxicity was analyzed using a Annexin-V/DAPI staining method in a panel of AML cell lines treated with vehicle or increasing concentrations of VEN for 72 h. Values are expressed as the percentage of viable cells for each condition relative to vehicle-treated cells. The ED50 values and leukemia cell lines used are described in the Figure. Oxygen consumption rate (OCR, b) was determined in vehicle- or metformin- (1 mM), VEN (1 μM) or combination treatment in K562 cells using a high-resolution respirometry. OCR was measured over time. Extracellular acidification rate (ECAR, c) was evaluated in the same set as OCR by Seahorse XF96 analyzer. Bar plots summarizing the data regarding the basal OCR (d), maximum OCR (e), mitochondrial ATP production (f), and basal ECAR (g) for K562 and U937 cells. Bar graphs represent normalized values expressed as a percentage for each sample relative to vehicle-treated controls. Groups were compared using Mixed-effect analysis with Dunn`s multiple comparison test. (h) Oxygen consumption rate (OCR) was measured in U937 cells treated with vehicle or metformin (1 mM) for 24 and 48 hours using high-resolution respirometry. Bar plots summarize basal OCR (i) and spare respiratory capacity (SRC, j) in U937 cells under the same conditions. Groups were compared using Mixed-effect analysis with Dunn`s multiple comparison test. (k, l) U937 and K562 cells were treated for 72 hours with increasing concentrations of venetoclax (VEN) and metformin, with synergy assessed using the Bliss coefficient (ZIP Score > 10 indicating synergism). (m) Weekly peripheral blood sampling of K562-transplanted NSG mice was performed to monitor human CD45^+^ chimerism and disease progression. At sacrifice, human CD45^+^ K562 cells in bone marrow and spleen were analyzed by flow cytometry. Violin plots depict K562 cell engraftment in the bone marrow (n) and spleen (o) at sacrifice. Data are presented as median values. * P<0.05, ** P<0.01, *** P<0.001. Groups were compared using Mixed-effect analysis with Dunn`s multiple comparison test. (p) Oxygen Consumption Rate (OCR) was measured in primary AML cells treated with vehicle, metformin (1 mM), venetoclax (VEN, 1 μM), or their combination using high-resolution respirometry (representative OCR plot for AML#1). A bar plot summarizes the maximum OCR values (q). (r) Extracellular acidification rate (ECAR) was assessed in the same experimental setup as in panel p. (s) A bar plot presents the summarized ECAR measurements from three independent AML samples. OCR and ECAR were evaluated using a Seahorse XF96 analyzer. Bar graphs display normalized values as a percentage relative to vehicle-treated controls. Dots on the plots indicate the number of biological replicates, with each representing the average of at least four independent technical replicates plated for Seahorse experiments. Groups were compared using Mixed-effect analysis with Dunn`s multiple comparison test.

Table S1.

Raw data supporting figure 1d

Table S2.

De-anonymized patient data related to figures 2a-c

| **Seq** | **Sex (M/F)** | **Treatment** | **Age** | **Risk_AGR** | **CR** | **OS_**  **months** | **OS_**  **status** | **DFS_**  **months** | **DFS_**  **status** | **CIR_**  **status** | **mtDNA_**  **continuous** | **mtDNA_**  **groups** | **WBC** |
| --- | --- | --- | --- | --- | --- | --- | --- | --- | --- | --- | --- | --- | --- |
| 1 | M | 3+7 | 39.83 | Intermediate | 0 | 0.59 | 1 |  |  | 2 | 0.637 | 0 | 96400 |
| 2 | M | 3+7 | 20.16 | Intermediate | 1 | 45.09 | 1 | 9.23 | 1 | 1 | 1.569 | 0 | 50200 |
| 3 | F | 3+7 | 66.66 | Favorable | 0 | 0.1 | 1 |  |  | 2 | 0.616 | 0 | 166100 |
| 4 | F | 3+7 | 21.54 | Adverse | 1 | 5.81 | 1 | 3.42 | 1 | 1 | 1.102 | 0 | 45500 |
| 5 | F | 3+7 | 44.05 | Favorable | 1 | 35.07 | 0 | 7.75 | 1 | 1 | 0.57 | 0 | 62600 |
| 6 | F | 3+7 | 69.75 | Intermediate | 0 | 1.71 | 1 |  |  | 2 | 1.079 | 0 | 61200 |
| 7 | M | Palliative care | 57.18 | Intermediate | 0 | 0.76 | 1 |  |  | 2 | 0.57 | 0 | 2600 |
| 8 | F | 3+7 | 33.35 | Intermediate | 0 | 1.67 | 1 |  |  | 2 | 1.376 | 0 | 47500 |
| 9 | F | 3+7 | 69.69 | Intermediate | 1 | 22.07 | 1 | 12.38 | 1 | 1 | 0.737 | 0 | 2800 |
| 10 | M | 3+7 | 21.82 | Favorable | 1 | 9.13 | 0 | 6.93 | 0 | 0 | 0.415 | 0 | 71200 |
| 11 | F | Palliative care | 65.12 | Adverse | 0 | 0.53 | 1 |  |  | 2 | 0.507 | 0 | 118500 |
| 12 | F | Palliative care | 77.91 | Intermediate | 0 | 1.35 | 1 |  |  | 2 | 1.149 | 0 | 66000 |
| 13 | F | 3+7 | 69.39 | Adverse | 0 | 0.79 | 1 |  |  | 2 | 0.418 | 0 | 112800 |
| 14 | F | Palliative care | 72.56 | Intermediate | 0 | 1.71 | 1 |  |  | 2 | 1.647 | 1 | 3900 |
| 15 | F | Palliative care | 70.64 | Intermediate | 1 | 6.24 | 1 | 3.15 | 1 | 1 | 0.222 | 0 | 36400 |
| 16 | F | 3+7 | 19.12 | Favorable | 0 | 1.97 | 1 |  |  | 2 | 3.945 | 1 | 63400 |
| 17 | F | 3+7 | 34.6 | Favorable | 1 | 16.32 | 1 | 4.76 | 1 | 1 | 1.765 | 1 | 54300 |
| 18 | M | Palliative care | 32.6 | Intermediate | 0 | 0.53 | 1 |  |  | 2 |  |  | 5200 |
| 19 | F | Palliative care | 46.59 | Favorable | 0 | 0.69 | 1 |  |  | 2 | 0.946 | 0 | 113900 |
| 20 | F | 3+7 | 21.78 | Intermediate | 1 | 3.09 | 1 | 1.51 | 1 | 1 | 0.986 | 0 | 25800 |
| 21 | M | 3+7 | 35.13 | Intermediate | 1 | 53.46 | 1 | 35.53 | 1 | 1 | 0.237 | 0 | 1900 |
| 22 | M | 3+7 | 30.42 | Intermediate | 1 | 46.54 | 1 | 21.94 | 1 | 1 | 0.398 | 0 | 11600 |
| 23 | M | 3+7 | 69.68 | Favorable | 0 | 2.66 | 1 |  |  | 2 | 0.412 | 0 | 17600 |
| 24 | M | 3+7 | 58.54 | Intermediate | 0 | 2.43 | 1 |  |  | 2 | 0.521 | 0 | 1700 |
| 25 | M | 3+7 | 56.02 | Favorable | 1 | 12.78 | 0 | 7.13 | 1 | 1 | 0.629 | 0 | 22000 |
| 26 | F | Palliative care | 60.94 | Intermediate | 0 | 0.3 | 1 |  |  | 2 | 0.774 | 0 | 59000 |
| 27 | M | Palliative care | 63.39 | Intermediate | 0 | 1.08 | 1 |  |  | 2 | 3.34 | 1 | 217870 |
| 28 | M | Palliative care | 57.04 | Favorable | 0 | 0.1 | 1 |  |  | 2 | 1.338 | 0 | 187620 |
| 29 | F | Palliative care | 81.56 | Adverse | 0 | 2.33 | 1 |  |  | 2 | 3.63 | 1 | 1550 |
| 30 | F | 3+7 | 46.9 | Intermediate | 0 | 1.41 | 1 |  |  | 2 | 0.291 | 0 | 35200 |
| 31 | M | Palliative care | 61 | Favorable | 0 | 0.72 | 1 |  |  | 2 | 0.914 | 0 | 137700 |
| 32 | F | 3+7 | 51.05 | Adverse | 1 | 3.45 | 1 | 0.92 | 1 | 1 | 0.237 | 0 | 79900 |
| 33 | M | 3+7 | 38.73 | Favorable | 1 | 9.89 | 0 | 6.4 | 0 | 0 | 0.785 | 0 | 1800 |
| 34 | M | 3+7 | 19.56 | Intermediate | 1 | 65.78 | 1 | 1.18 | 1 | 1 | 0.927 | 0 | 285700 |
| 35 | F | Palliative care | 83.35 | Intermediate | 1 | 32.68 | 0 | 31.69 | 0 | 0 | 0.476 | 0 | 1900 |
| 36 | F | 3+7 | 24.79 | Favorable | 0 | 1.02 | 1 |  |  | 2 | 0.629 | 0 | 13900 |
| 37 | M | 3+7 | 52.5 | Intermediate | 0 | 1.22 | 1 |  |  | 2 | 0.536 | 0 | 117000 |
| 38 | F | Palliative care | 68.73 | Intermediate | 0 | 2.1 | 1 |  |  | 2 | 6.821 | 1 | 31100 |
| 39 | M | 3+7 | 64.45 | Intermediate | 1 | 16.29 | 1 | 13.73 | 1 | 1 | 1.464 | 0 | 116500 |
| 40 | F | Palliative care | 71.78 | Intermediate | 1 | 31.53 | 0 | 30.54 | 0 | 0 | 0.966 | 0 | 1300 |
| 41 | F | 3+7 | 49.87 | Intermediate | 1 | 8.57 | 1 | 5.29 | 1 | 1 | 0.5 | 0 | 1200 |
| 42 | M | 3+7 | 24.24 | Favorable | 1 | 21.94 | 0 | 11.66 | 1 | 1 | 0.742 | 0 | 5700 |
| 43 | F | 3+7 | 66.47 | Intermediate | 1 | 4.43 | 1 | 0.79 | 1 | 1 | 0.277 | 0 | 3200 |
| 44 | M | 3+7 | 43.15 | Adverse | 0 | 1.38 | 1 |  |  | 2 | 0.551 | 0 | 62000 |
| 45 | M | 3+7 | 57.85 | Intermediate | 0 | 1.02 | 1 |  |  | 2 | 1.028 | 0 | 18800 |
| 46 | F | Palliative care | 18.38 | Favorable | 0 | 0.16 | 1 |  |  | 2 | 0.57 | 0 | 192950 |
| 47 | M | Palliative care | 74.44 | Intermediate | 1 | 7.22 | 1 | 1.22 | 1 | 1 | 1.149 | 0 | 23800 |
| 48 | M | Palliative care | 26.18 | Favorable | 0 | 0.33 | 1 |  |  | 2 |  |  | 113180 |
| 49 | M | 3+7 | 31.58 | Adverse | 0 | 0.82 | 1 |  |  | 2 | 0.463 | 0 | 29900 |
| 50 | F | 3+7 | 40.25 | Favorable | 0 | 1.48 | 1 |  |  | 2 | 0.966 | 0 | 109800 |
| 51 | F | Palliative care | 18.18 | Favorable | 0 | 0.1 | 1 |  |  | 2 | 1.133 | 0 | 211230 |
| 52 | F | Palliative care | 57.71 | Favorable | 0 | 0.36 | 1 |  |  | 2 | 0.92 | 0 | 13700 |
| 53 | F | 3+7 | 64.01 | Adverse | 1 | 28.57 | 1 | 12.87 | 1 | 1 | 0.369 | 0 | 33200 |
| 54 | M | 3+7 | 39.32 | Adverse | 0 | 0.59 | 1 |  |  | 2 | 0.732 | 0 | 60900 |
| 55 | M | Palliative care | 74.62 | Intermediate | 0 | 0.43 | 1 |  |  | 2 | 0.493 | 0 | 2300 |
| 56 | M | Palliative care | 78.42 | Favorable | 0 | 1.67 | 1 |  |  | 2 | 0.859 | 0 | 31100 |
| 57 | F | 3+7 | 22.07 | Intermediate | 1 | 6.27 | 1 | 2.04 | 1 | 1 | 0.46 | 0 | 3600 |
| 58 | M | 3+7 | 49.65 | Adverse | 1 | 6.14 | 1 | 2.04 | 1 | 1 | 1.149 | 0 | 13300 |
| 59 | F | 3+7 | 58.12 | Favorable | 1 | 15.44 | 1 | 2.79 | 1 | 1 | 1.625 | 0 | 2820 |
| 60 | F | 3+7 | 26.23 | Intermediate | 1 | 27.59 | 0 | 26.6 | 0 | 0 | 0.374 | 0 | 14100 |
| 61 | M | Palliative care | 65.06 | Intermediate | 1 | 3.91 | 1 | 1.22 | 0 | 2 | 1.548 | 0 | 26000 |
| 62 | M | Palliative care | 76.67 | Intermediate | 1 | 26.86 | 0 | 25.85 | 0 | 0 | 1.149 | 0 | 490 |
| 63 | F | Palliative care | 59.07 | Favorable | 0 | 0.03 | 1 |  |  | 2 | 1.753 | 1 | 181230 |
| 64 | F | 3+7 | 24.36 | Intermediate | 0 | 4.5 | 1 |  |  | 2 | 2.158 | 1 | 16500 |
| 65 | M | 3+7 | 49.12 | Favorable | 1 | 71.53 | 0 | 69.43 | 0 | 0 | 0.283 | 0 | 8460 |
| 66 | M | 3+7 | 62.25 | Favorable | 0 | 1.51 | 1 |  |  | 2 | 1.141 | 0 | 60640 |
| 67 | F | Palliative care | 71.44 | Adverse | 1 | 3.74 | 1 | 1.22 | 0 | 2 | 1.815 | 1 | 29800 |
| 68 | M | Palliative care | 74.79 | Intermediate | 0 | 0.46 | 1 |  |  | 2 | 1.11 | 0 | 109200 |
| 69 | F | Palliative care | 71.07 | Intermediate | 0 | 1.81 | 1 |  |  | 2 | 1.434 | 0 | 43900 |
| 70 | M | 3+7 | 37.77 | Adverse | 1 | 3.22 | 1 | 1.38 | 1 | 1 | 1.464 | 0 | 48800 |
| 71 | M | 3+7 | 56.35 | Intermediate | 0 | 4.43 | 1 |  |  | 2 | 1.636 | 1 | 40300 |
| 72 | F | 3+7 | 63.1 | Favorable | 1 | 7.91 | 1 | 5.52 | 1 | 1 | 2.585 | 1 | 40870 |
| 73 | F | 3+7 | 47.8 | Intermediate | 1 | 12.15 | 1 | 4.56 | 1 | 1 | 1.292 | 0 | 49200 |
| 74 | M | Palliative care | 44.63 | Intermediate | 1 | 3.97 | 1 | 1.22 | 0 | 2 | 1.454 | 0 | 10900 |
| 75 | F | 3+7 | 60.69 | Intermediate | 0 | 8.9 | 1 |  |  | 2 | 1.338 | 0 | 140350 |
| 76 | M | 3+7 | 33.94 | Adverse | 1 | 5.39 | 0 | 0.53 | 0 | 0 | 0.747 | 0 | 49500 |
| 77 | M | Palliative care | 87.78 | Intermediate | 1 | 4.6 | 0 | 4.63 | 0 | 0 | 0.796 | 0 | 26300 |
| 78 | M | Palliative care | 76.7 | Favorable | 1 | 3.58 | 1 | 1.22 | 0 | 2 | 1.444 | 0 | 4970 |
| 79 | F | Palliative care | 37.47 | Favorable | 0 | 0.53 | 1 |  |  | 2 | 0.966 | 0 | 18150 |
| 80 | F | 3+7 | 61.58 | Intermediate | 1 | 8.34 | 0 | 4.66 | 0 | 0 | 0.245 | 0 | 242500 |
| 81 | M | 3+7 | 39.99 | Adverse | 1 | 1.51 | 0 | 0.1 | 0 | 0 | 1.613 | 0 | 156000 |
| 82 | F | 3+7 | 50.02 | Adverse | 1 | 7.52 | 1 | 5.68 | 1 | 1 | 0.807 | 0 | 180900 |
| 83 | F | Palliative care | 80.34 | Favorable | 0 | 2.07 | 1 |  |  | 2 | 1.729 | 1 | 35800 |
| 84 | M | Palliative care | 67.69 | Intermediate | 0 | 0.53 | 1 |  |  | 2 | 0.582 | 0 | 23930 |
| 85 | M | 3+7 | 65.19 | Intermediate | 1 | 16.75 | 0 | 15.76 | 0 | 0 | 0.48 | 0 | 2760 |
| 86 | F | 3+7 | 56.62 | Favorable | 1 | 64.3 | 0 | 13.4 | 1 | 1 | 1.157 | 0 | 108020 |
| 87 | M | 3+7 | 28.25 | Adverse | 1 | 7.32 | 0 | 1.18 | 1 | 1 | 0.774 | 0 | 23900 |
| 88 | F | 3+7 | 60.36 | Intermediate | 0 | 2.63 | 1 |  |  | 2 | 0.678 | 0 | 18850 |
| 89 | M | Palliative care | 75.38 | Intermediate | 0 | 1.48 | 1 |  |  | 2 | 1.11 | 0 | 70290 |
| 90 | F | 3+7 | 32.82 | Favorable | 1 | 3.22 | 1 | 1.18 | 1 | 1 | 1.064 | 0 | 39200 |
| 91 | M | 3+7 | 49.36 | Intermediate | 1 | 19.18 | 1 | 15.37 | 1 | 1 | 1.385 | 0 | 7000 |
| 92 | M | 3+7 | 45.95 | Intermediate | 1 | 6.73 | 0 | 5.48 | 0 | 0 | 2.042 | 1 | 164260 |
| 93 | F | 3+7 | 25.69 | Favorable | 1 | 14.48 | 1 | 9.98 | 1 | 1 | 0.946 | 0 | 231570 |
| 94 | F | Palliative care | 62.08 | Intermediate | 0 | 0.95 | 1 |  |  | 2 | 1.064 | 0 | 100000 |
| 95 | M | 3+7 | 47.79 | Intermediate | 1 | 55.04 | 0 | 6.11 | 1 | 1 | 14.42 | 1 | 16200 |
| 96 | F | 3+7 | 67.34 | Favorable | 1 | 6.9 | 0 | 6.24 | 0 | 0 | 1.125 | 0 | 3290 |
| 97 | M | 3+7 | 28.98 | Adverse | 1 | 7.59 | 0 | 6.01 | 0 | 0 | 1.395 | 0 | 19200 |
| 98 | F | Palliative care | 65.07 | Intermediate | 0 | 0.62 | 1 |  |  | 2 | 1.558 | 0 | 79860 |
| 99 | M | 3+7 | 61.56 | Intermediate | 0 | 1.44 | 1 |  |  | 2 | 1.064 | 0 | 2050 |
| 100 | M | Palliative care | 60.66 | Intermediate | 0 | 0.2 | 1 |  |  | 2 | 1.028 | 0 | 247600 |
| 101 | M | 3+7 | 56.05 | Intermediate | 1 | 7.65 | 0 | 4.04 | 0 | 0 | 1.919 | 1 | 600 |
| 102 | F | 3+7 | 64.54 | Intermediate | 1 | 22.07 | 0 | 19.93 | 0 | 0 | 1.206 | 0 | 22000 |
| 103 | F | Palliative care | 76.44 | Intermediate | 1 | 8.9 | 1 | 1.22 | 1 | 1 | 1.404 | 0 | 1600 |
| 104 | M | 3+7 | 56.46 | Adverse | 0 | 1.74 | 1 |  |  | 2 | 2.297 | 1 | 178800 |
| 105 | M | 3+7 | 51.7 | Intermediate | 0 | 2.43 | 1 |  |  | 2 | 2.144 | 1 | 175100 |
| 106 | F | 3+7 | 42.06 | Adverse | 1 | 5.06 | 0 | 2.99 | 0 | 0 | 4.17 | 1 | 3670 |
| 107 | F | Palliative care | 44 | Favorable | 0 | 0.53 | 1 |  |  | 2 | 0.877 | 0 | 312500 |
| 108 | F | Palliative care | 39.06 | Intermediate | 0 | 0.56 | 1 |  |  | 2 | 0.633 | 0 | 73700 |
| 109 | F | 3+7 | 66.67 | Intermediate | 1 | 19.9 | 1 | 17.64 | 1 | 1 | 0.595 | 0 | 37500 |
| 110 | M | 3+7 | 64.48 | Adverse | 1 | 2.46 | 1 | 0.13 | 1 | 1 | 14.42 | 1 | 167500 |
| 111 | M | 3+7 | 23.35 | Adverse | 1 | 8.87 | 1 | 1.38 | 1 | 1 | 1.094 | 0 | 124000 |
| 112 | M | 3+7 | 26.6 | Favorable | 1 | 6.21 | 0 | 4.6 | 0 | 0 | 8.282 | 1 | 8500 |
| 113 | F | Palliative care | 90.84 | Adverse | 0 | 0.07 | 1 |  |  | 2 | 1.866 | 1 | 234700 |
| 114 | F | 3+7 | 20.94 | Intermediate | 1 | 25.32 | 0 | 3.02 | 1 | 1 | 2.129 | 1 | 64210 |
| 115 | M | 3+7 | 37.71 | Intermediate | 1 | 6.57 | 1 | 5.02 | 1 | 1 | 1.125 | 0 | 55700 |
| 116 | M | 3+7 | 42.54 | Favorable | 1 | 3.02 | 0 | 1.44 | 0 | 0 | 0.16 | 0 | 31300 |
| 117 | F | 3+7 | 64.19 | Intermediate | 1 | 9.52 | 0 | 6.5 | 0 | 0 | 1.189 | 0 | 34230 |
| 118 | M | Palliative care | 93.76 | Intermediate | 0 | 0.49 | 1 |  |  | 2 | 3.387 | 1 | 99100 |
| 119 | M | Palliative care | 41.56 | Intermediate | 0 | 0.56 | 1 |  |  | 2 | 1.189 | 0 | 93080 |
| 120 | F | 3+7 | 50.99 | Favorable | 1 | 17.93 | 0 | 13.04 | 0 | 0 | 1.007 | 0 | 74400 |
| 121 | M | Palliative care | 68.51 | Favorable | 0 | 0.85 | 1 |  |  | 2 | 1.659 | 1 | 90200 |
| 122 | F | Palliative care | 81.22 | Intermediate | 0 | 0.1 | 1 |  |  | 2 | 1.548 | 0 | 61600 |
| 123 | F | 3+7 | 26.42 | Intermediate | 0 | 1.18 | 1 |  |  | 2 | 0.801 | 0 | 36500 |
| 124 | F | 3+7 | 64.39 | Intermediate | 1 | 2.5 | 0 | 1.58 | 0 | 0 | 0.847 | 0 | 78900 |
| 125 | F | 3+7 | 66.34 | Adverse | 1 | 6.24 | 1 | 5.25 | 1 | 1 | 14.723 | 1 |  |
| 126 | M | 3+7 | 57.75 | Favorable | 1 | 3.45 | 0 | 2 | 0 | 0 | 1.385 | 0 | 120690 |
| 127 | M | 3+7 | 67.78 | Intermediate | 1 | 3.05 | 0 | 1.58 | 0 | 0 | 1.404 | 0 | 50220 |
| 128 | M | 3+7 | 49.96 | Intermediate | 1 | 2.53 | 0 | 1.12 | 0 | 0 | 2.969 | 1 | 60250 |
| 129 | M | 3+7 | 33.47 | Favorable | 1 | 2.53 | 0 | 1.12 | 0 | 0 | 1.149 | 0 | 186700 |
| 130 | M | 3+7 | 31.09 | Intermediate | 1 | 9.92 | 1 | 7.52 | 1 | 1 | 0.953 | 0 | 300000 |
| 131 | F | 3+7 | 58.54 | Intermediate | 0 | 1.97 | 1 |  |  | 2 | 0.747 | 0 | 36800 |
| 132 | M | 3+7 | 21.8 | Favorable | 1 | 32.35 | 0 | 3.28 | 1 | 1 | 0.669 | 0 | 14800 |
| 133 | M |  | 64.37 | Favorable | 1 | 36.06 | 0 | 25.62 | 0 | 0 | 0.901 | 0 | 2960 |
| 134 | F |  | 65.48 | Favorable | 0 | 0.26 | 1 |  |  | 2 | 0.779 | 0 | 46780 |
| 135 | F | 3+7 | 29.07 | Intermediate | 1 | 4.83 | 1 | 0.46 | 1 | 1 | 0.642 | 0 | 58910 |
| 136 | F | 3+7 | 25.49 | Intermediate | 1 | 24.17 | 0 | 22.33 | 0 | 0 | 0.097 | 0 | 51790 |
| 137 | M | 3+7 | 61.55 | Adverse | 0 | 1.25 | 1 |  |  | 2 | 1.376 | 0 | 60060 |
| 138 | F | Palliative care | 61.39 | Favorable | 1 | 8.08 | 0 | 2.2 | 1 | 1 | 1.558 | 0 |  |
| 139 | M | Palliative care | 74.36 | Adverse | 1 | 8.64 | 1 | 7.65 | 1 | 1 | 2.549 | 1 | 174000 |
| 140 | M | 3+7 | 63.84 | Adverse | 0 | 3.45 | 1 |  |  | 2 | 1.853 | 1 | 13100 |
| 141 | F | 3+7 | 27.84 | Favorable | 1 | 22.56 | 1 | 7.36 | 1 | 1 | 7.311 | 1 | 18000 |
| 142 | M | 3+7 | 67.19 | Intermediate | 1 | 10.9 | 1 | 0.36 | 1 | 1 | 2.949 | 1 | 79430 |
| 143 | F |  | 80.01 | Adverse | 0 | 0.07 | 1 |  |  | 2 | 0.392 | 0 | 22600 |
| 144 | F | Palliative care | 35.51 | Intermediate | 0 | 0.3 | 1 |  |  | 2 | 2.158 | 1 | 435940 |
| 145 | F | 3+7 | 47.48 | Favorable | 1 | 24.2 | 0 | 21.71 | 0 | 0 | 1.892 | 1 | 119300 |
| 146 | M |  | 38.42 | Favorable | 1 | 7.88 | 0 | 6.77 | 0 | 0 | 1.24 | 0 |  |
| 147 | M | Palliative care | 59.39 | Intermediate | 0 | 1.25 | 1 |  |  | 2 | 2.297 | 1 | 91740 |
| 148 | F | 3+7 | 63.35 | Intermediate | 1 | 27.95 | 0 | 1.44 | 0 | 0 | 1.905 | 1 | 1670 |
| 149 | M |  | 40.05 | Favorable | 1 | 24.76 | 0 | 23.84 | 0 | 0 | 4.79 | 1 | 120000 |
| 150 | M | 3+7 | 36.07 | Favorable | 1 | 15.53 | 0 | 14.25 | 0 | 0 | 0.514 | 0 | 165900 |
| 151 | F | 3+7 | 21.36 | Intermediate | 0 | 2.13 | 1 |  |  | 2 | 4.579 | 1 | 69210 |
| 152 | F | Palliative care | 61.25 | Intermediate | 0 | 0.53 | 1 |  |  | 0 | 3.492 | 1 | 87270 |
| 153 | M | 3+7 | 58.57 | Intermediate | 1 | 2.86 | 1 | 1.22 | 1 | 1 | 3.482 | 1 | 11890 |
| 154 | F | 3+7 | 43.61 | Intermediate | 0 | 5.29 | 1 |  |  | 2 | 5.352 | 1 | 28460 |
| 155 | M | Palliative care | 61.61 | Intermediate | 0 | 0.46 | 1 |  |  | 2 | 8.491 | 1 | 31150 |
| 156 | F | 3+7 | 35.15 | Favorable | 1 | 12.15 | 1 | 3.61 | 1 | 1 | 3.681 | 1 | 78290 |
| 157 | M | Palliative care | 69.47 | Intermediate | 1 | 20.2 | 0 | 19.24 | 0 | 0 | 0.463 | 0 | 227550 |
| 158 | F | 3+7 | 52.45 | Intermediate | 1 | 5.55 | 0 | 1.12 | 0 | 0 | 0.068 | 0 | 1890 |
| 159 | F |  | 49.55 | Favorable | 0 | 1.25 | 1 |  |  | 2 |  |  | 63720 |
| 160 | F | Palliative care | 61.98 | Intermediate | 0 | 0.33 | 1 |  |  | 0 |  |  | 21630 |
| 161 | M | Palliative care | 72.94 | Intermediate | 0 | 1.15 | 1 |  |  | 0 |  |  | 93310 |
| 162 | M | Palliative care | 70.05 | Favorable | 0 | 1.97 | 1 |  |  | 0 |  |  | 12630 |
| 163 | F | Palliative care | 84.97 | Favorable | 0 | 0.33 | 1 |  |  | 0 |  |  | 145140 |
| 164 | F | 3+7 | 42 | Adverse | 1 | 5.39 | 1 | 2.59 | 0 | 2 | 6.063 | 1 |  |
| 165 | F | 3+7 | 24 | Adverse | 0 | 11.69 | 1 |  |  | 2 | 7.311 | 1 |  |
| 166 | M | 3+7 | 28 | Adverse | 1 | 46.77 | 1 | 42.3 | 1 | 1 | 0.139 | 0 |  |
| 167 | M | 3+7 | 59 | Intermediate | 1 | 7.55 | 1 | 5.98 | 1 | 1 | 0.801 | 0 |  |
| 168 | F | 3+7 | 38 | Favorable | 1 | 5.25 | 1 | 3.97 | 0 | 2 | 1.972 | 1 |  |
| 169 | F | 3+7 | 46 | Adverse | 1 | 28.01 | 1 | 26.86 | 0 | 2 | 1.007 | 0 |  |
| 170 | F | 3+7 | 48 | Intermediate | 0 | 18.23 | 1 |  |  | 2 | 0.818 | 0 |  |
| 171 | F | 3+7 | 41 | Favorable | 0 | 3.02 | 1 |  |  | 2 | 2.158 | 1 |  |
| 172 | F | 3+7 | 45 | Intermediate | 1 | 3.88 | 1 | 1.31 | 1 | 1 | 0.332 | 0 |  |
| 173 | M | 3+7 | 40 | Intermediate | 1 | 12.32 | 1 | 11.17 | 0 | 2 | 0.877 | 0 |  |
| 174 | M | 3+7 | 22.18 | Intermediate | 0 | 10.8 | 1 |  |  | 2 | 0.272 | 0 |  |
| 175 | F | 3+7 | 27.09 | Intermediate | 1 | 5.94 | 1 | 3.65 | 0 | 2 | 0.418 | 0 |  |
| 176 | M | 3+7 | 53.84 | Adverse | 0 | 3.25 | 1 |  |  | 2 | 7.16 | 1 |  |
| 177 | F | 3+7 | 18 | Intermediate | 1 | 23.71 | 1 | 6.24 | 1 | 1 | 0.219 | 0 |  |
| 178 | M | 3+7 | 58.35 | Favorable | 0 | 0.59 | 1 |  |  | 2 | 0.192 | 0 |  |
| 179 | F | 3+7 | 60.44 | Intermediate | 0 | 17.18 | 1 |  |  | 2 | 1.028 | 0 |  |
| 180 | M | 3+7 | 35.14 | Intermediate | 0 | 1.48 | 1 |  |  | 2 |  |  |  |
| 181 | M | 3+7 | 24.69 | Intermediate | 0 | 1.87 | 1 |  |  | 2 | 0.08 | 0 |  |
| 182 | M | 3+7 | 48.58 | Favorable | 0 | 0.16 | 1 |  |  | 2 | 0.171 | 0 |  |
| 183 | F | 3+7 | 48.74 | Favorable | 0 | 8.64 | 1 |  |  | 2 | 0.204 | 0 |  |
| 184 | F | 3+7 | 40.51 | Intermediate | 0 | 0.1 | 1 |  |  | 2 | 0.049 | 0 |  |
| 185 | F | 3+7 | 30.58 | Favorable | 0 | 1.15 | 1 |  |  | 2 | 0.157 | 0 |  |
| 186 | M | 3+7 | 37 | Adverse | 1 | 11.2 | 1 | 5.29 | 1 | 1 | 0.233 | 0 |  |
| 187 | F | 3+7 | 38 | Intermediate | 0 | 0.66 | 1 |  |  | 2 | 6.869 | 1 |  |
| 188 | M | 3+7 | 43.21 | Adverse | 0 | 1.28 | 1 |  |  | 2 | 0.578 | 0 |  |
| 189 | F | 3+7 | 24.39 | Adverse | 0 | 12.38 | 1 |  |  | 2 | 0.069 | 0 |  |
| 190 | M | 3+7 | 21.54 | Favorable | 0 | 0.03 | 1 |  |  | 2 | 0.192 | 0 |  |
| 191 | M | 3+7 | 41.23 | Favorable | 1 | 39.18 | 1 | 20.2 | 1 | 1 | 0.299 | 0 |  |
| 192 | F | 3+7 | 50.1 | Adverse | 1 | 13.4 | 1 | 7.49 | 1 | 1 | 1.853 | 1 |  |
| 193 | M | 3+7 | 58.77 | Adverse | 0 | 0.39 | 1 |  |  | 2 | 0.871 | 0 |  |
| 194 | M | 3+7 | 28.19 | Adverse | 0 | 0.1 | 1 |  |  | 2 | 0.476 | 0 |  |
| 195 | M | 3+7 | 52.41 | Intermediate | 1 | 4.07 | 1 | 2.82 | 0 | 2 | 0.92 | 0 |  |
| 196 | F | 3+7 | 26.23 | Adverse | 0 | 0.56 | 1 |  |  | 2 | 1.231 | 0 |  |
| 197 | F | 3+7 | 24.79 | Intermediate | 0 | 2.23 | 1 |  |  | 2 | 0.351 | 0 |  |
| 198 | M | 3+7 | 31.68 | Favorable | 1 | 2.92 | 1 | 0.95 | 1 | 1 | 2.329 | 1 |  |
| 199 | M | 3+7 | 29.22 | Adverse | 1 | 36.58 | 1 | 35.04 | 1 | 1 | 0.801 | 0 |  |
| 200 | M | 3+7 | 22.97 | Adverse | 0 | 2.79 | 1 |  |  | 2 | 4.757 | 1 |  |
| 201 | F | 3+7 | 45 | Adverse | 0 | 7.36 | 1 |  |  | 2 | 1.197 | 0 |  |
| 202 | F | 3+7 | 22 | Adverse | 1 | 6.24 | 0 | 2.99 | 0 | 0 | 0.57 | 0 |  |
| 203 | F | 3+7 | 47 | Intermediate | 1 | 57.14 | 0 | 40.16 | 0 | 0 | 0.262 | 0 |  |
| 204 | F | 3+7 | 46 | Intermediate | 1 | 52.91 | 0 | 29.03 | 0 | 0 | 0.785 | 0 |  |
| 205 | M | 3+7 | 18 | Favorable | 1 | 36.09 | 0 | 33.92 | 0 | 0 | 0.274 | 0 |  |
| 206 | M | 3+7 | 37.04 | Intermediate | 1 | 102.04 | 0 | 99.74 | 0 | 0 | 1.028 | 0 |  |
| 207 | F | 3+7 | 40.13 | Favorable | 1 | 106.54 | 0 | 14.65 | 1 | 1 | 0.92 | 0 |  |
| 208 | F | 3+7 | 24.29 | Intermediate | 1 | 101.77 | 0 | 100.49 | 0 | 0 | 0.012 | 0 |  |
| 209 | F | 3+7 | 25.72 | Favorable | 1 | 100.89 | 0 | 98.82 | 0 | 0 | 0.707 | 0 |  |
| 210 | M | 3+7 | 22.03 | Favorable | 1 | 95.67 | 0 | 94.75 | 0 | 0 | 0.21 | 0 |  |
| 211 | M | 3+7 | 35 | Favorable | 1 | 92.38 | 0 | 48.93 | 1 | 1 | 4.084 | 1 |  |
| 212 | F | 3+7 | 44.11 | Favorable | 1 | 92.22 | 0 | 90.77 | 0 | 0 | 1.329 | 0 |  |
| 213 | F | 3+7 | 41.19 | Intermediate | 1 | 79.74 | 0 | 78.62 | 0 | 0 | 0.973 | 0 |  |
| 214 | F | 3+7 | 29.61 | Favorable | 1 | 67.95 | 0 | 66.31 | 0 | 0 | 0.049 | 0 |  |
| 215 | F | 3+7 | 59 | Favorable | 1 | 57.5 | 0 | 56.29 | 0 | 0 | 0.403 | 0 |  |
| 216 | M | 3+7 | 59.94 | Adverse | 0 | 0.26 | 1 |  |  | 2 | 1.959 | 1 |  |
| 217 | F | 3+7 | 22.64 | Intermediate | 1 | 2.04 | 0 | 0.89 | 1 | 1 | 4.097 | 1 | 16780 |
| 218 | M | 3+7 | 36.24 | Favorable | 1 | 25.06 | 0 | 8.14 | 1 | 1 | 3.141 | 1 |  |
| 219 | M | 3+7 | 42.12 | Favorable | 0 | 0.95 | 1 |  |  | 2 | 6.061 | 1 | 27290 |
| 220 | M | 3+7 | 45.49 | Adverse | 1 | 0.76 | 1 | 0.49 | 1 | 1 | 4.098 | 1 |  |
| 221 | M | 3+7 | 18.17 | Favorable | 1 | 31.92 | 0 | 1.48 | 1 | 1 | 5.086 | 1 |  |
| 222 | M | 3+7 | 58.51 | Adverse | 0 | 4.3 | 1 |  |  | 2 | 0.078 | 0 | 34010 |
| 223 | F | 3+7 | 64.33 | Intermediate | 1 | 2.3 | 1 | 0.99 | 1 | 1 | 3.295 | 1 | 136770 |
| 224 | M | 3+7 | 60.17 | Intermediate | 1 | 15.01 | 0 | 2.27 | 1 | 1 | 6.341 | 1 |  |
| 225 | M | 3+7 | 20.04 | Favorable | 1 | 17.5 | 0 | 16.12 | 0 | 0 | 3.054 | 1 | 12360 |
| 226 | M | 3+7 | 56.03 | Intermediate | 1 | 13.3 | 0 | 11.76 | 0 | 0 | 4.71 | 1 | 4070 |
| 227 | F | 3+7 | 51.59 | Adverse | 1 | 11.79 | 0 | 5.06 | 1 | 1 | 3.331 | 1 | 1180 |
| 228 | M | 3+7 | 52.72 | Favorable | 0 | 0.99 | 1 |  |  | 2 | 2.004 | 1 | 7300 |
| 229 | F | 3+7 | 44.53 | Intermediate | 1 | 8.24 | 0 | 4.96 | 1 | 1 | 17.164 | 1 |  |
| 230 | F | 3+7 | 56.66 | Intermediate | 0 | 3.28 | 1 |  |  | 2 | 17.361 | 1 | 259370 |
| 231 | F | 3+7 | 63.65 | Adverse | 1 | 10.11 | 1 | 7.88 | 1 | 1 | 1.906 | 1 |  |
| 232 | M | 3+7 | 51.26 | Intermediate | 1 | 5.58 | 0 | 2.53 | 0 | 0 | 0.248 | 0 | 33780 |
| 233 | M | 3+7 | 35.22 | Intermediate | 1 | 11.66 | 1 | 5.12 | 1 | 1 | 2.902 | 1 | 114000 |
| 234 | M | 3+7 | 51.33 |  | 1 | 6.4 | 0 | 3.74 | 1 | 1 | 8.454 | 1 |  |
| 235 | F | 3+7 | 51.16 | Intermediate | 1 | 5.42 | 0 | 15.89 | 0 | 0 | 1.919 | 1 | 2900 |
| 236 | F | 3+7 | 60.99 | Favorable | 1 | 22.99 | 0 | 21.41 | 0 | 0 | 3.418 | 1 | 21900 |
| 237 | M | 3+7 | 54.06 | Favorable | 1 | 5.81 | 1 | 3.84 | 0 | 2 | 2.29 | 1 | 2060 |
| 238 | F | 3+7 | 59.12 | Favorable | 1 | 18.78 | 1 | 17.18 | 0 | 2 | 2.156 | 1 | 3090 |
| 239 | F | 3+7 | 51.79 | Favorable | 1 | 23.19 | 0 | 21.9 | 0 | 0 | 3.261 | 1 | 12770 |
| 240 | F | 3+7 | 50.03 | Favorable | 1 | 20.82 | 0 | 19.77 | 0 | 0 | 4.626 | 1 | 65680 |
| 241 | F | 3+7 | 27.39 | Intermediate | 1 | 13.63 | 1 | 12.45 | 0 | 2 | 3.975 | 1 | 4170 |
| 242 | F | 3+7 | 33.55 | Intermediate | 0 | 13.66 | 1 |  |  | 2 | 4.307 | 1 | 9400 |
| 243 | M | 3+7 | 39.02 | Intermediate | 1 | 10.25 | 0 | 9.13 | 0 | 0 | 3.598 | 1 | 154700 |
| 244 | F | 3+7 | 51.2 | Intermediate | 1 | 21.48 | 0 | 20.3 | 0 | 0 | 3.553 | 1 | 52530 |
| 245 | M | 3+7 | 62.84 | Intermediate | 0 | 8.7 | 1 |  |  | 2 | 3.915 | 1 | 208900 |
| 246 | F | 3+7 | 33.43 | Adverse | 1 | 4.14 | 1 | 0.82 | 1 | 1 | 1.009 | 0 | 15000 |
| 247 | F | 3+7 | 59.07 | Adverse | 0 | 0.82 | 1 |  |  | 2 | 1.024 | 0 | 1200 |
| 248 | F | 3+7 | 37.59 | Intermediate | 1 | 26.8 | 0 | 25.55 | 0 | 0 | 2.792 | 1 | 5600 |
| 249 | M | 3+7 | 42.44 | Intermediate | 1 | 20.69 | 0 | 14.61 | 1 | 1 | 9.436 | 1 | 201800 |
| 250 | M | 3+7 | 60.56 | Intermediate | 1 | 18.33 | 0 | 12.25 | 1 | 1 | 2.542 | 1 | 138200 |
| 251 | F | 3+7 | 61.45 | Intermediate | 1 | 9.79 | 1 | 6.47 | 1 | 1 | 2.433 | 1 | 52490 |
| 252 | F | 3+7 | 25.17 | Adverse | 0 | 35.17 | 0 |  |  | 0 | 1.909 | 1 | 3670 |
| 253 | F | 3+7 | 63.81 | Favorable | 0 | 0.76 | 1 |  |  | 2 | 1.943 | 1 | 7390 |
| 254 | F | 3+7 | 57.75 | Adverse | 1 | 1.67 | 1 | 0.62 | 1 | 1 | 1.974 | 1 | 10560 |
| 255 | F | 3+7 | 61.45 | Intermediate | 1 | 10.48 | 1 | 3.68 | 1 | 1 | 3.123 | 1 | 52490 |
| 256 | F | 3+7 | 53.77 | Intermediate | 0 | 0.69 | 1 |  |  | 2 | 2.353 | 1 | 1120 |
| 257 | M | 3+7 | 23.39 | Favorable | 1 | 21.61 | 0 | 19.93 | 0 | 0 | 3.044 | 1 | 138200 |
| 258 | F | 3+7 | 22.77 | Favorable | 1 | 46.17 | 0 | 44.96 | 0 | 0 | 2.799 | 1 | 2000 |
| 259 | F | 3+7 | 50.79 | Favorable | 0 | 9.46 | 1 |  |  | 2 | 1.282 | 0 | 6900 |
| 260 | M | 3+7 | 26.67 | Intermediate | 1 | 2.4 | 1 | 1.02 | 1 | 1 | 6.717 | 1 | 15000 |
| 261 | M | 3+7 | 33.97 | Intermediate | 0 | 4.11 | 1 |  |  | 2 | 1.051 | 0 | 51800 |
| 262 | F | 3+7 | 50.63 | Intermediate | 1 | 35.44 | 0 | 33.43 | 0 | 0 | 2.509 | 1 | 15200 |
| 263 | F | 3+7 | 21.89 | Adverse | 1 | 3.45 | 1 | 1.41 | 1 | 1 | 3.906 | 1 | 1500 |
| 264 | M | 3+7 | 27.62 | Adverse | 0 | 10.64 | 1 |  |  | 2 | 1.887 | 1 | 1740 |
| 265 | F | 3+7 | 37.59 | Intermediate | 1 | 10.25 | 0 | 16.49 | 0 | 0 | 1.03 | 0 | 5600 |
| 266 | F | 3+7 | 28.31 | Intermediate | 1 | 7 | 1 | 4.43 | 1 | 1 | 1.327 | 0 | 10450 |
| 267 | M | 3+7 | 61.15 | Intermediate | 0 | 18.75 | 1 |  |  | 2 | 2.459 | 1 | 1540 |
| 268 | F | 3+7 | 53.97 | Favorable | 1 | 12.74 | 1 | 3.42 | 1 | 1 | 2.564 | 1 | 10180 |
| 269 | F | 3+7 | 66.47 | Favorable | 0 | 1.87 | 1 |  |  | 2 | 2.09 | 1 | 5560 |
| 270 | F | 3+7 | 26.88 | Favorable | 1 | 7.26 | 0 | 21.87 | 0 | 0 | 1.159 | 0 | 3180 |
| 271 | F | 3+7 | 62.46 | Intermediate | 1 | 9.79 | 1 | 7.95 | 1 | 1 | 0.536 | 0 | 10040 |
| 272 | F | 3+7 | 53.7 | Favorable | 1 | 29.39 | 0 | 2.56 | 0 | 0 | 1.805 | 1 | 6210 |
| 273 | F | 3+7 | 69.75 | Favorable | 1 | 12.28 | 0 | 11.2 | 1 | 1 | 2.835 | 1 | 1900 |
| 274 | M | 3+7 | 74.93 | Favorable | 0 | 1.02 | 1 |  |  | 2 | 2.457 | 1 | 26200 |
| 275 | M | 3+7 | 30.95 | Intermediate | 1 | 16.45 | 0 | 18.39 | 0 | 0 | 1.372 | 0 | 26510 |
| 276 | M | 3+7 | 42.44 | Favorable | 1 | 20.69 | 0 | 19.7 | 0 | 0 | 0.708 | 0 | 201800 |
| 277 | F | 3+7 | 63.81 | Favorable | 0 | 0.76 | 1 |  |  | 2 | 1.019 | 0 | 7390 |
| 278 | F | 3+7 | 20.35 | Adverse | 1 | 27.42 | 0 | 25.78 | 1 | 1 | 9.095 | 1 | 14470 |
| 279 | M | 3+7 | 29.19 | Intermediate | 1 | 17.7 | 1 | 4.17 | 1 | 1 | 5.899 | 1 | 206140 |
| 280 | F | 3+7 | 54.78 | Intermediate | 1 | 3.68 | 0 | 10.25 | 0 | 0 | 2.66 | 1 | 6390 |
| 281 | F | 3+7 | 45.81 | Favorable | 1 | 4.99 | 0 | 3.97 | 0 | 0 | 2.735 | 1 | 70650 |
| 282 | F | Palliative care | 80.37 | Adverse | 1 | 120.39 | 0 | 119.05 | 0 | 0 | 0.013 | 0 | 21800 |
| 283 | F | Other | 48.13 | Favorable | 1 | 124.2 | 0 | 71.2 | 1 | 1 | 0.56 | 0 | 11000 |
| 284 | F | Other | 28.99 | Intermediate | 1 | 22.66 | 1 | 7.13 | 1 | 1 | 14.621 | 1 | 3100 |
| 285 | F | Other | 41.07 | Intermediate | 1 | 31.36 | 1 | 29.16 | 1 | 1 | 0.459 | 0 | 30600 |
| 286 | F | Other | 29.34 | Adverse | 1 | 8.37 | 1 | 4.14 | 1 | 1 | 0.242 | 0 | 65900 |
| 287 | M | Other | 35.9 | Adverse | 0 | 0.56 | 1 |  |  | 2 |  |  | 4700 |
| 288 | F | Other | 34.72 | Intermediate | 1 | 7.32 | 1 | 5.94 | 0 | 2 |  |  | 1250 |
| 289 | F | Other | 43.92 | Favorable | 1 | 111.46 | 0 | 110.38 | 0 | 0 |  |  | 5000 |
| 290 | F | Palliative care | 78.78 |  | 0 | 0.69 | 1 |  |  | 2 |  |  | 2000 |
| 291 | F | Palliative care | 93.12 | Intermediate | 0 | 0.36 | 1 |  |  | 2 |  |  | 117000 |
| 292 | F | 3+7 | 38.21 | Favorable | 1 | 106.11 | 0 | 105.16 | 0 | 0 | 0.575 | 0 | 25500 |
| 293 | M | Palliative care | 75.86 | Adverse | 0 | 2.69 | 1 |  |  | 2 |  |  | 5100 |
| 294 | F | 3+7 | 58.51 | Adverse | 0 | 0.76 | 1 |  |  | 2 |  |  | 29700 |
| 295 | M | 3+7 | 49.32 | Intermediate | 0 | 0.1 | 1 |  |  | 2 |  |  | 33300 |
| 296 | F | 3+7 | 59.66 | Intermediate | 0 | 0.56 | 1 |  |  | 2 |  |  | 15700 |
| 297 | F | 3+7 | 27.3 |  | 0 | 2.33 | 1 |  |  | 2 |  |  | 8200 |
| 298 | F | 3+7 | 60.34 | Intermediate | 1 | 10.67 | 1 | 8.87 | 1 | 1 | 2.735 | 1 | 8400 |
| 299 | F | 3+7 | 43.56 | Intermediate | 1 | 7.22 | 1 | 5.45 | 0 | 2 | 0.013 | 0 | 24900 |
| 300 | F | 3+7 | 66.29 | Intermediate | 1 | 86.21 | 1 | 45.75 | 1 | 1 | 0.56 | 0 | 126500 |
| 301 | M | Palliative care | 92.97 | Adverse | 0 | 0.26 | 1 |  |  | 2 | 1.247 | 0 | 18600 |
| 302 | M | 3+7 | 53.41 | Adverse | 1 | 11.07 | 1 | 9.72 | 1 | 1 | 0.775 | 0 | 1500 |
| 303 | F | 3+7 | 39.98 | Intermediate | 1 | 74.38 | 1 | 61.22 | 1 | 1 |  |  | 3900 |
| 304 | M | 3+7 | 29.36 | Adverse | 1 | 53.69 | 1 | 48.28 | 1 | 1 | 0.625 | 0 | 62000 |
| 305 | F | Palliative care | 78.37 | Adverse | 0 | 0.03 | 1 |  |  | 2 | 0.398 | 0 | 97900 |
| 306 | F | 3+7 | 28.15 | Adverse | 1 | 3.19 | 1 | 2.17 | 0 | 2 | 0.297 | 0 | 281000 |
| 307 | M | 3+7 | 54.5 | Intermediate | 0 | 0.72 | 1 |  |  | 2 |  |  | 121700 |
| 308 | F | 3+7 | 38.7 | Intermediate | 1 | 7.75 | 1 | 6.57 | 0 | 2 |  |  | 6800 |
| 309 | F | 3+7 | 59.03 | Adverse | 1 | 16.45 | 1 | 14.38 | 1 | 1 | 0.225 | 0 | 8500 |
| 310 | F | 3+7 | 61.09 | Favorable | 0 | 1.05 | 1 |  |  | 2 | 0.275 | 0 | 80000 |
| 311 | F | 3+7 | 24.11 | Favorable | 1 | 7.16 | 1 | 5.98 | 0 | 2 | 1.193 | 0 | 18400 |
| 312 | M | 3+7 | 51.82 | Intermediate | 0 | 0.03 | 1 |  |  | 2 |  |  | 37100 |
| 313 | F | 3+7 | 57.03 | Intermediate | 1 | 60.66 | 0 | 59.24 | 0 | 0 |  |  | 102700 |
| 314 | M | 3+7 | 31.78 | Favorable | 1 | 19.21 | 1 | 9.29 | 1 | 1 |  |  | 4600 |
| 315 | F | Palliative care | 73.75 | Intermediate | 1 | 18.06 | 1 | 7.32 | 1 | 1 | 5.295 | 1 | 2300 |
| 316 | M | Palliative care | 86.92 | Adverse | 0 | 10.64 | 1 |  |  | 2 | 0.116 | 0 | 790000 |
| 317 | F | 3+7 | 39.97 | Intermediate | 1 | 9.06 | 1 | 3.19 | 1 | 1 | 0.278 | 0 | 59500 |
| 318 | M | 3+7 | 42.29 | Intermediate | 1 | 38.39 | 0 | 37.01 | 0 | 0 | 0.246 | 0 | 121900 |
| 319 | M | 3+7 | 45.34 | Favorable | 1 | 35.67 | 0 | 33.99 | 0 | 0 | 0.187 | 0 | 12400 |
| 320 | M | 3+7 | 32.17 | Intermediate | 0 | 0.53 | 1 |  |  | 2 | 0.32 | 0 | 34900 |
| 321 | M | 3+7 | 22.8 | Intermediate | 1 | 34.06 | 0 | 32.91 | 0 | 0 | 0.482 | 0 | 17500 |
| 322 | M | 3+7 | 63.93 | Adverse | 1 | 34.78 | 0 | 33.56 | 0 | 0 | 0.484 | 0 | 26600 |
| 323 | M | 3+7 | 41.49 | Favorable | 1 | 33.1 | 0 | 31.89 | 0 | 0 |  |  | 30300 |
| 324 | F | 3+7 | 65.83 | Favorable | 0 | 1.28 | 1 |  |  | 2 | 0.808 | 0 | 119500 |
| 325 | M | Palliative care | 87.79 | Intermediate | 1 | 36.55 | 0 | 35.99 | 1 | 1 | 0.264 | 0 | 1300 |
| 326 | M | 3+7 | 67.95 | Intermediate | 1 | 64.63 | 0 | 61.22 | 0 | 0 | 0.297 | 0 | 41000 |
| 327 | M | 3+7 | 23.34 | Intermediate | 1 | 80.59 | 0 | 79.44 | 0 | 0 | 0.294 | 0 | 2100 |
| 328 | M | 3+7 | 53.74 | Intermediate | 0 | 1.12 | 1 |  |  | 2 | 1.024 | 0 | 3900 |
| 329 | F | 3+7 | 55.57 | Intermediate | 1 | 9.29 | 1 | 1.67 | 1 | 1 | 4.416 | 1 | 2000 |
| 330 | F | 3+7 | 59.22 | Favorable | 0 | 0.62 | 1 |  |  | 2 | 0.177 | 0 | 40500 |
| 331 | F | Palliative care | 76.28 | Favorable | 0 | 0.2 | 1 |  |  | 2 | 3.723 | 1 | 6600 |
| 332 | F | Palliative care | 79.88 | Favorable | 0 | 1.08 | 1 |  |  | 2 | 0.952 | 0 | 61500 |
| 333 | F |  | 78.24 | Intermediate | 1 | 70.94 | 0 | 69.23 | 0 | 0 | 3.503 | 1 | 1300 |
| 334 | F | 3+7 | 56.86 | Intermediate | 0 | 0.59 | 1 |  |  | 2 | 3.92 | 1 | 6400 |
| 335 | M | Palliative care | 81.86 | Intermediate | 0 | 0.36 | 1 |  |  | 2 | 1.218 | 0 | 27100 |
| 336 | F | 3+7 | 62.26 | Adverse | 0 | 1.22 | 1 |  |  | 2 |  |  | 1800 |
| 337 | F | 3+7 | 35.51 | Intermediate | 0 | 0.66 | 1 |  |  | 2 | 5.992 | 1 | 15900 |
| 338 | F |  | 39.82 | Intermediate | 1 | 71.95 | 0 | 70.61 | 0 | 0 | 2.404 | 1 | 119400 |
| 339 | F | 3+7 | 48.05 | Adverse | 1 | 62.69 | 0 | 61.54 | 0 | 0 | 0.767 | 0 | 16500 |
| 340 | F | 3+7 | 54.97 | Intermediate | 1 | 6.73 | 1 | 5.65 | 0 | 2 | 3.411 | 1 | 15800 |
| 341 | F | 3+7 | 56.14 | Adverse | 0 | 0.43 | 1 |  |  | 2 | 0.343 | 0 | 56900 |
| 342 | M | 3+7 | 46.72 | Favorable | 1 | 53.76 | 0 | 52.12 | 0 | 0 | 4.396 | 1 | 6100 |
| 343 | F | Palliative care | 70.15 | Favorable | 1 | 58.1 | 0 | 46.17 | 0 | 0 | 2.898 | 1 | 66300 |
| 344 | F | 3+7 | 31.91 | Favorable | 0 | 0.53 | 1 |  |  | 2 | 2.325 | 1 | 1500 |
| 345 | F | 3+7 | 49.2 | Favorable | 1 | 61.08 | 0 | 59.34 | 0 | 0 | 0.269 | 0 | 74400 |
| 346 | M | Palliative care | 74.33 | Intermediate | 0 | 0.39 | 1 |  |  | 2 | 2.761 | 1 | 2300 |
| 347 | M | Palliative care | 60.95 | Intermediate | 0 | 0.95 | 1 |  |  | 2 |  |  | 66700 |
| 348 | F | 3+7 | 57.54 | Intermediate | 1 | 56.91 | 0 | 55.37 | 0 | 0 | 0.342 | 0 | 341300 |
| 349 | F | Palliative care | 69.95 | Adverse | 0 | 0.89 | 1 |  |  | 2 | 2.376 | 1 | 860 |
| 350 | M | 3+7 | 60.24 | Favorable | 1 | 55.47 | 0 | 53.3 | 0 | 0 | 21.397 | 1 | 1560 |
| 351 | M | 3+7 | 44.04 | Intermediate | 1 | 2.63 | 1 | 0.53 | 0 | 2 | 0.813 | 0 | 13800 |
| 352 | F | 3+7 | 49.63 | Intermediate | 0 | 0.33 | 1 |  |  | 2 |  |  | 75800 |
| 353 | F | 3+7 | 60.42 | Favorable | 0 | 0.66 | 1 |  |  | 2 |  |  | 10600 |
| 354 | M | Palliative care | 67.86 | Favorable | 0 | 6.93 | 1 |  |  | 2 | 2.644 | 1 | 19100 |
| 355 | F | Palliative care | 71.67 | Favorable | 0 | 0.07 | 1 |  |  | 2 | 6.912 | 1 | 5200 |
| 356 | M | Palliative care | 63.44 | Adverse | 0 | 2.53 | 1 |  |  | 2 | 3.13 | 1 | 27800 |
| 357 | M | 3+7 | 65.44 | Intermediate | 0 | 0.92 | 1 |  |  | 2 | 2.669 | 1 | 49700 |
| 358 | F | Palliative care | 77.79 | Adverse | 0 | 2.2 | 1 |  |  | 2 | 1.426 | 0 | 5800 |
| 359 | M | 3+7 | 47.86 | Adverse | 1 | 7.09 | 1 | 5.62 | 1 | 1 | 2.475 | 1 | 20600 |
| 360 | F | Palliative care | 76.85 | Intermediate | 0 | 2.23 | 1 |  |  | 2 | 6.515 | 1 | 1000 |
| 361 | M | Palliative care | 76.97 | Intermediate | 0 | 8.37 | 1 |  |  | 2 | 5.171 | 1 | 1400 |
| 362 | F | Palliative care | 82.48 | Intermediate | 0 | 1.05 | 1 |  |  | 2 | 1.214 | 0 | 77600 |
| 363 | M | 3+7 | 61.44 | Intermediate | 0 | 0.85 | 1 |  |  | 2 | 0.307 | 0 | 4900 |
| 364 | F | 3+7 | 41.41 | Favorable | 0 | 0.46 | 1 |  |  | 2 | 3.785 | 1 | 92200 |
| 365 | F | 3+7 | 43.38 | Favorable | 0 | 0.23 | 1 |  |  | 2 |  |  | 144300 |
| 366 | F | 3+7 | 34.06 | Favorable | 1 | 27.88 | 0 | 26.96 | 0 | 0 | 0.27 | 0 | 133000 |
| 367 | M | 3+7 | 49.2 | Intermediate | 0 | 0.92 | 1 |  |  | 2 | 2.811 | 1 | 1200 |
| 368 | F | Palliative care | 85.98 | Adverse | 0 | 0.43 | 1 |  |  | 2 | 0.521 | 0 | 7200 |
| 369 | F | Palliative care | 83.28 | Favorable | 0 | 0.1 | 1 |  |  | 2 | 0.17 | 0 | 87100 |
| 370 | F | 3+7 | 53.38 | Favorable | 1 | 4.6 | 1 | 3.35 | 0 | 2 | 0.449 | 0 | 6200 |
| 371 | F | 3+7 | 67.01 | Favorable | 1 | 4.17 | 1 | 0.66 | 1 | 1 | 2.485 | 1 | 98200 |
| 372 | M | 3+7 | 66.11 | Intermediate | 0 | 0.72 | 1 |  |  | 2 | 0.648 | 0 | 206300 |
| 373 | M | 3+7 | 60.56 | Adverse | 1 | 4.33 | 1 | 3.02 | 0 | 2 | 0.684 | 0 | 138200 |
| 374 | F | 3+7 | 20.27 | Adverse | 1 | 25.81 | 0 | 23.22 | 0 | 0 | 0.529 | 0 | 14500 |
| 375 | M | 3+7 | 29.19 | Intermediate | 1 | 5.71 | 1 | 4.56 | 0 | 2 |  |  | 235500 |
| 376 | M | 3+7 | 55.39 | Intermediate | 1 | 22.79 | 0 | 21.08 | 0 | 0 | 0.208 | 0 | 13500 |
| 377 | F | 3+7 | 61.78 | Favorable | 0 | 0.56 | 1 |  |  | 2 | 0.697 | 0 | 45500 |
| 378 | M | 3+7 | 61.74 | Intermediate | 1 | 2.36 | 1 | 1.05 | 0 | 2 | 0.386 | 0 | 680 |
| 379 | M | 3+7 | 23.96 | Favorable | 1 | 21.61 | 0 | 20.66 | 0 | 0 | 0.5 | 0 | 74600 |
| 380 | F | 3+7 | 59.12 | Adverse | 1 | 16.06 | 1 | 3.94 | 1 | 1 | 0.689 | 0 | 3100 |
| 381 | M | 3+7 | 54.06 | Intermediate | 1 | 5.81 | 1 | 1.38 | 1 | 1 | 0.671 | 0 | 2000 |
| 382 | F | 3+7 | 59.89 | Intermediate | 1 | 6.63 | 1 | 5.42 | 0 | 2 | 0.766 | 0 | 1500 |
| 383 | F | Palliative care | 75.98 | Intermediate | 1 | 7.03 | 1 | 3.94 | 0 | 2 | 0.805 | 0 | 205200 |
| 384 | F | 3+7 | 26.67 | Intermediate | 1 | 13.63 | 1 | 8.8 | 1 | 1 | 1.383 | 0 | 2800 |
| 385 | M | 3+7 | 55.33 | Favorable | 0 | 0.72 | 1 |  |  | 2 | 1.094 | 0 | 52000 |
| 386 | F | 3+7 | 28.52 | Intermediate | 1 | 3.05 | 0 | 1.38 | 0 | 0 | 0.378 | 0 | 70600 |
| 387 | F | 3+7 | 45.82 | Adverse | 1 | 3.45 | 1 | 2.2 | 1 | 1 | 0.631 | 0 | 121500 |
| 388 | M | 3+7 | 51.62 | Intermediate | 1 | 13.86 | 0 | 11.46 | 0 | 0 | 0.733 | 0 | 33800 |
| 389 | M | Palliative care | 64.96 | Intermediate | 0 | 2.59 | 1 |  |  | 2 | 0.575 | 0 | 3300 |
| 390 | F | Palliative care | 75.33 | Intermediate | 0 | 0.53 | 1 |  |  | 2 | 1.842 | 1 | 18200 |
| 391 | M | 3+7 | 43.89 | Favorable | 1 | 8.9 | 0 | 7.42 | 0 | 0 | 1.092 | 0 | 3800 |
| 392 | M | 3+7 | 68.98 | Intermediate | 0 | 0.33 | 1 |  |  | 2 | 4.176 | 1 | 176500 |
| 393 | M | Palliative care | 69.4 | Intermediate | 0 | 4.2 | 1 |  |  | 2 | 0.715 | 0 | 8400 |
| 394 | M | 3+7 | 56.16 | Intermediate | 1 | 3.94 | 0 | 2.99 | 0 | 0 | 1.206 | 0 | 21900 |
| 395 | F | 3+7 | 63.44 | Intermediate | 0 | 0.3 | 1 |  |  | 2 | 4.252 | 1 | 39200 |
| 396 | F | 3+7 | 62.15 | Intermediate | 1 | 2.13 | 0 | 0.69 | 0 | 0 | 0.451 | 0 | 2100 |
| 397 | F | Ara-C + Imatinibe | 62.23 | Adverse | 1 | 28.57 | 0 | 27.22 | 0 | 0 | 0.37 | 0 | 5000 |
| 398 | F | 3+7 (Dauno) | 34.55 | Intermediate | 1 | 1.67 | 1 | 0.16 | 0 | 2 | 0.744 | 0 | 12000 |
| 399 | M | 3+7 (Dauno) | 30.89 | Adverse | 1 | 18.88 | 0 | 17.7 | 0 | 0 | 0.416 | 0 | 1000 |
| 400 | F | 3+7 (Dauno) | 45.67 | Intermediate | 1 | 12.48 | 1 | 7.36 | 1 | 1 | 3.069 | 1 | 35000 |
| 401 | F | 3+7 (Dauno) | 50.13 | Intermediate | 1 | 5.75 | 1 | 2.3 | 1 | 1 | 0.953 | 0 | 7000 |
| 402 | M | 3+7 (Dauno) | 62.93 | Intermediate | 0 | 1.41 | 1 |  |  | 2 | 0.757 | 0 | 55000 |
| 403 | M | 3+7 (Dauno) | 52.72 | Favorable | 0 | 0.99 | 1 |  |  | 2 | 0.428 | 0 | 7000 |
| 404 | F | 3+7 (Dauno) | 63.65 | Adverse | 1 | 10.15 | 1 | 8.8 | 1 | 1 | 0.302 | 0 | 6000 |
| 405 | F | 3+7 (Dauno) | 33.17 | Adverse | 1 | 12.71 | 1 | 8.11 | 1 | 1 | 2.735 | 1 | 17000 |
| 406 | M | 3+7 (Dauno) | 19.65 | Favorable | 1 | 11.99 | 1 | 10.77 | 1 | 1 | 2.042 | 1 | 62000 |
| 407 | F | 3+7 (Dauno) | 62.61 | Adverse | 1 | 9.52 | 1 | 7.75 | 1 | 1 | 0.56 | 0 | 23000 |
| 408 | F | 3+7 (Dauno) | 21.63 | Intermediate | 1 | 13.4 | 0 | 12.68 | 0 | 0 | 0.888 | 0 | 34000 |
| 409 | M | 3+7 | 21.98 | Adverse | 1 | 79.87 | 0 | 78.75 | 0 | 0 | 2.245 | 1 | 47000 |
| 410 | M | 3+7 (Dauno) | 31.91 | Intermediate | 1 | 45.55 | 1 | 16.29 | 1 | 1 | 3.408 | 1 | 1000 |
| 411 | F | 3+7 | 26.24 | Favorable | 1 | 67.22 | 0 | 66.21 | 0 | 0 | 1.208 | 0 | 8000 |
| 412 | M | 3+7 | 18.14 | Favorable | 1 | 65.62 | 0 | 64.6 | 0 | 0 | 0.636 | 0 | 4000 |
| 413 | M | 3+7 | 20.65 | Favorable | 1 | 3.28 | 1 | 1.12 | 0 | 2 |  |  | 3000 |
| 414 | F | Ara-C | 77.08 | Adverse | 0 | 1.48 | 1 |  |  | 2 |  |  | 3000 |
| 415 | M | 3+7 (Dauno) | 48.08 | Intermediate | 1 | 27.85 | 1 | 23.15 | 1 | 1 | 1.309 | 0 | 30000 |
| 416 | F | Ara-C SC | 68.57 | Intermediate | 1 | 16.68 | 1 | 15.01 | 0 | 2 | 0.193 | 0 | 19000 |
| 417 | M | Ara-C SC | 67.84 | Intermediate | 1 | 43.02 | 1 | 41.05 | 1 | 1 | 1.256 | 0 | 62000 |
| 418 | M | 3+7 (Dauno) | 57.41 | Intermediate | 1 | 31.2 | 0 | 29.16 | 0 | 0 | 0.307 | 0 | 2000 |
| 419 | M | 3+7 (Dauno) | 35.29 | Favorable | 1 | 61.31 | 0 | 60.26 | 0 | 0 | 0.846 | 0 | 37000 |
| 420 | M | 3+7 (Dauno) | 61.23 | Intermediate | 1 | 11.72 | 1 | 8.74 | 1 | 1 | 0.33 | 0 | 11000 |
| 421 | F | 3+7 (Dauno) | 61.28 | Adverse | 0 | 24.47 | 1 |  |  | 2 | 0.538 | 0 | 3000 |
| 422 | M | 3+7 (Dauno) | 54.16 | Intermediate | 1 | 38.19 | 1 | 17.7 | 1 | 1 | 1.216 | 0 | 61000 |
| 423 | F | Other | 75.12 | Intermediate | 1 | 24.7 | 1 | 18.85 | 1 | 1 | 0.901 | 0 | 4000 |
| 424 | F | HiDAC | 58.24 | Adverse | 1 | 3.38 | 1 | 2.53 | 0 | 2 | 0.264 | 0 | 1000 |
| 425 | M | 3+7 (Dauno) | 55.98 | Favorable | 1 | 57.24 | 0 | 55.86 | 0 | 0 | 0.499 | 0 | 17000 |
| 426 | M | 3+7 (Dauno) | 38.93 | Favorable | 1 | 52.15 | 0 | 11 | 1 | 1 |  |  | 2000 |
| 427 | F | 3+7 (Dauno) | 68.7 | Intermediate | 0 | 1.48 | 1 |  |  | 2 | 0.323 | 0 | 2000 |
| 428 | M | Ara-C SC | 87.81 | Favorable | 0 | 1.38 | 1 |  |  | 2 | 0.174 | 0 | 105000 |
| 429 | M | 3+7 (Dauno) | 51.61 | Intermediate | 1 | 54.88 | 0 | 53.4 | 0 | 0 | 0.283 | 0 | 8000 |
| 430 | M |  | 72.66 | Favorable | 1 | 6.67 | 0 | 4.6 | 0 | 0 |  |  | 1000 |
| 431 | F | Ara-C SC | 64.47 | Intermediate | 1 | 26.96 | 0 | 25.32 | 0 | 0 |  |  | 2000 |
| 432 | M | Ara-C SC | 75.89 | Favorable | 1 | 52.41 | 0 | 44.86 | 0 | 0 | 0.281 | 0 | 45000 |
| 433 | M | 3+7 (Dauno) | 60.55 | Favorable | 0 | 0.56 | 1 |  |  | 2 | 0.276 | 0 | 2000 |
| 434 | M | Ara-C SC | 69.96 | Intermediate | 1 | 36.12 | 1 | 29.52 | 1 | 1 | 0.866 | 0 | 2000 |
| 435 | M | Ara-C SC | 79.2 | Adverse | 0 | 9.59 | 1 |  |  | 2 |  |  | 14000 |
| 436 | M | 3+7 (Dauno) | 46.43 | Intermediate | 1 | 2.3 | 0 | 0.36 | 0 | 0 | 0.891 | 0 | 35000 |
| 437 | M | Ara-C SC | 68.76 | Adverse | 0 | 6.47 | 1 |  |  | 2 | 0.193 | 0 | 4000 |
| 438 | F | 3+7 (Dauno) | 37.57 | Intermediate | 0 | 1.05 | 1 |  |  | 2 | 1.022 | 0 | 51000 |
| 439 | F | 3+7 (Dauno) | 45.42 | Intermediate | 1 | 4.89 | 0 | 3.81 | 0 | 0 | 0.231 | 0 | 160000 |
| 440 | M | 3+7 (Dauno) | 72.78 | Favorable | 0 | 2.66 | 1 |  |  | 2 | 0.362 | 0 | 8000 |
| 441 | M | 3+7 (Dauno) | 20.02 | Favorable | 1 | 45.25 | 0 | 44.4 | 0 | 0 | 0.188 | 0 | 8000 |
| 442 | M | Ara-C SC | 79.85 | Intermediate | 0 | 11.07 | 1 |  |  | 2 | 0.465 | 0 | 16000 |
| 443 | F | 3+7 (Dauno) + dasatinib | 28.12 | Adverse | 1 | 2.07 | 0 | 1.12 | 0 | 0 | 1.399 | 0 | 37000 |
| 444 | M | 3+7 (Dauno) + dasatinib | 37.77 | Intermediate | 1 | 12.35 | 1 | 8.77 | 1 | 1 |  |  | 30000 |
| 445 | M | 3+7 (Dauno) | 68.27 | Favorable | 1 | 20.03 | 1 | 16.91 | 1 | 1 | 0.298 | 0 | 27000 |
| 446 | F | HiDAC | 59.95 | Intermediate | 0 | 1.41 | 1 |  |  | 2 | 0.272 | 0 | 135000 |
| 447 | M | 3+7 (Dauno) | 37.67 | Favorable | 1 | 33.1 | 0 | 12.02 | 1 | 1 |  |  | 18000 |
| 448 | M | 3+7 (Dauno) | 67.83 | Adverse | 1 | 7.52 | 1 | 6.08 | 1 | 1 | 0.652 | 0 | 5000 |
| 449 | M | Ara-C SC | 73.29 | Adverse | 0 | 0.99 | 1 |  |  | 2 | 0.39 | 0 | 127000 |
| 450 | M | Ara-C SC | 65 | Intermediate | 0 | 0.66 | 1 |  |  | 2 | 0.183 | 0 | 14000 |
| 451 | F | 3+7 (Dauno) | 40.58 | Favorable | 1 | 39.18 | 0 | 6.34 | 1 | 1 | 0.739 | 0 | 36000 |
| 452 | F | 3+7 (Dauno) | 20.92 | Favorable | 1 | 23.22 | 0 | 22.3 | 0 | 0 | 0.14 | 0 | 3000 |
| 453 | F | Ara-C SC | 70.96 | Intermediate | 0 | 1.05 | 1 |  |  | 2 | 0.257 | 0 | 3000 |
| 454 | F | Ara-C SC | 78.03 | Adverse | 0 | 4.86 | 1 |  |  | 2 | 0.684 | 0 | 1000 |
| 455 | M | Azacitidin+Venetoclax | 79.12 | Intermediate | 1 | 15.67 | 0 | 14.25 | 0 | 0 | 0.904 | 0 | 25000 |
| 456 | M | 3+7 (Dauno) | 50.15 | Adverse | 1 | 10.08 | 1 | 8.97 | 1 | 1 | 1.575 | 0 | 319000 |
| 457 | F | 3+7 (Dauno) | 23.34 | Adverse | 1 | 43.55 | 0 | 42.4 | 0 | 0 | 0.527 | 0 | 2000 |
| 458 | M | 3+7 (Dauno) | 63.15 | Adverse | 0 | 0.46 | 1 |  |  | 2 | 1.206 | 0 | 289 |
| 459 | M | 3+7 (Dauno) | 49.03 | Adverse | 1 | 3.05 | 1 | 1.28 | 1 | 1 | 4.252 | 1 | 3000 |
| 460 | F | Ara-C SC | 77.42 | Adverse | 0 | 5.78 | 1 |  |  | 2 | 0.451 | 0 | 100000 |
| 461 | F | 3+7 (Dauno) | 71.13 | Intermediate | 0 | 0.56 | 1 |  |  | 2 | 0.37 | 0 | 100000 |
| 462 | F | 3+7 (Dauno) | 30.61 | Adverse | 1 | 2.96 | 1 | 2 | 0 | 2 | 0.744 | 0 | 125000 |
| 463 | F | 3+7 (Dauno) | 50.63 | Adverse | 0 | 0.76 | 1 |  |  | 2 | 0.416 | 0 | 7000 |
| 464 | F | 3+7 (Dauno) | 50.55 | Favorable | 1 | 39.08 | 0 | 37.11 | 0 | 0 | 3.069 | 1 | 15000 |
| 465 | M | 3+7 (Dauno) | 52.59 | Intermediate | 1 | 3.51 | 0 | 1.94 | 0 | 0 | 0.953 | 0 | 2000 |
| 466 | M | Ara-C SC | 74.85 | Favorable | 0 | 1.02 | 1 |  |  | 2 | 0.757 | 0 | 11000 |
| 467 | M | 3+7 (Dauno) | 27.48 | Favorable | 1 | 32.02 | 0 | 29.92 | 0 | 0 | 0.428 | 0 | 57000 |
| 468 | F | 3+7 (Dauno) | 31.85 | Adverse | 0 | 1.9 | 1 |  |  | 2 | 0.302 | 0 | 52000 |
| 469 | F | 3+7 (Dauno) | 37.59 | Intermediate | 1 | 28.64 | 0 | 27.55 | 0 | 0 | 2.735 | 1 | 6000 |
| 470 | M | 3+7 (Dauno) | 45.09 | Intermediate | 1 | 28.24 | 0 | 1.28 | 1 | 1 | 2.042 | 1 | 6000 |
| 471 | F | 3+7 (Dauno) | 41.23 | Favorable | 1 | 1.97 | 1 | 1.05 | 0 | 2 | 0.56 | 0 | 11000 |
| 472 | F | 3+7 (Dauno) | 60.51 | Adverse | 1 | 25.16 | 0 | 24.27 | 0 | 0 | 0.888 | 0 | 22000 |
| 473 | F | 3+7 (Dauno) | 25.23 | Favorable | 0 | 0.62 | 1 |  |  | 2 | 2.245 | 1 | 43000 |
| 474 | M | 3+7 (Dauno) | 63 | Intermediate | 0 | 0.99 | 1 |  |  | 2 | 3.408 | 1 | 209000 |
| 475 | M | 3+7 (Dauno) | 58.8 | Adverse | 1 | 16.98 | 1 | 6.11 | 1 | 1 | 1.208 | 0 | 6000 |
| 476 | F | 3+7 (Dauno) | 21.89 | Adverse | 1 | 4.4 | 1 | 3.38 | 0 | 2 | 0.636 | 0 | 2000 |
| 477 | F | 3+7 | 42.41 | Intermediate | 1 | 3.51 | 0 | 2.46 | 0 | 0 | 0.499 | 0 | 69000 |
| 478 | M | 3+7 | 25.49 | Intermediate | 1 | 23.74 | 0 | 17.14 | 0 | 0 | 0.154 | 0 | 8900 |
| 479 | M | 3+7 | 23.12 | Adverse | 1 | 8.74 | 1 | 5.29 | 1 | 1 | 4.525 | 1 |  |
| 480 | M | 3+7 | 36.07 | Favorable | 1 | 32.18 | 0 | 30.9 | 0 | 0 | 1.18 | 0 | 165900 |
| 481 | F | 3+7 | 21.36 | Intermediate | 0 | 2.13 | 1 |  |  | 2 |  |  | 69210 |
| 482 | F | Palliative care | 61.25 | Intermediate | 0 | 0.53 | 1 |  |  | 2 |  |  | 87270 |
| 483 | M | 3+7 | 53.67 | Intermediate | 1 | 8.31 | 0 | 1.97 | 0 | 0 | 0.503 | 0 | 2040 |
| 484 | F | 3+7 | 43.61 | Intermediate | 0 | 5.29 | 1 |  |  | 2 |  |  | 28460 |
| 485 | F | 3+7 | 47.29 | Intermediate | 0 | 2.59 | 1 |  |  | 2 |  |  |  |
| 486 | M | Palliative care | 61.61 | Intermediate | 0 | 0.46 | 1 |  |  | 2 |  |  | 31150 |
| 487 | F | 3+7 | 45.79 | Intermediate | 0 | 0.82 | 1 |  |  | 2 |  |  | 71500 |
| 488 | F | 3+7 | 51.93 | Favorable | 1 | 3.84 | 0 | 2.53 | 0 | 0 | 0.112 | 0 |  |
| 489 | F | 3+7 | 35.15 | Favorable | 1 | 12.15 | 1 | 3.61 | 1 | 1 | 0.801 | 0 | 78290 |
| 490 | M | Palliative care | 69.47 | Intermediate | 1 | 1.12 | 0 | 19.24 | 0 | 0 | 0.091 | 0 | 227550 |
| 491 | F | 3+7 | 52.45 | Intermediate | 1 | 5.55 | 0 | 1.12 | 0 | 0 | 0.933 | 0 | 1890 |
| 492 | F | 3+7 | 49.51 | Favorable | 0 | 1.77 | 1 |  |  | 2 | 0.828 | 0 | 63720 |
| 493 | F | 3+7 | 26.92 | Intermediate | 1 | 8.64 | 1 | 5.55 | 1 | 1 | 4.278 | 1 | 92000 |
| 494 | F | Palliative care | 61.98 | Intermediate | 0 | 0.33 | 1 |  |  | 2 | 3.846 | 1 | 21630 |
| 495 | M | Palliative care | 72.94 | Intermediate | 0 | 1.15 | 1 |  |  | 2 |  |  | 93310 |
| 496 | F | 3+7 | 35.16 | Favorable | 1 | 23.48 | 0 | 20.39 | 0 | 0 | 0.318 | 0 | 50670 |
| 497 | M | 3+7 | 19.29 | Intermediate | 1 | 8.34 | 0 | 6.01 | 0 | 0 | 0.154 | 0 | 1710 |
| 498 | M | 3+7 | 42.97 | Adverse | 1 | 5.32 | 0 | 4.4 | 0 | 0 | 0.278 | 0 | 21900 |
| 499 | M | 3+7 | 57.74 | Adverse | 1 | 6.86 | 0 | 4.24 | 0 | 0 | 0.436 | 0 | 5500 |
| 500 | M | Palliative care | 70.05 | Favorable | 0 | 1.97 | 1 |  |  | 2 |  |  | 12630 |
| 501 | F | 3+7 | 68.18 | Adverse | 1 | 8.74 | 1 | 7.29 | 1 | 1 | 2.385 | 1 | 60000 |
| 502 | F | Palliative care | 84.97 | Adverse | 0 | 0.33 | 1 |  |  | 2 |  |  | 145140 |
| 503 | M | 3+7 | 66.75 | Intermediate | 0 | 3.65 | 1 |  |  | 2 |  |  | 6000 |
| 504 | M | 3+7 | 60.3 | Intermediate | 1 | 10.77 | 0 | 9.43 | 0 | 0 | 7.188 | 1 | 18700 |
| 505 | F | 3+7 | 26.51 | Intermediate | 0 | 2.69 | 1 |  |  | 2 | 11.479 | 1 | 2880 |
| 506 | F | 3+7 | 39.24 | Favorable | 1 | 16.06 | 0 | 13.2 | 0 | 0 | 0.027 | 0 | 20600 |
| 507 | M | 3+7 | 33.36 | Intermediate | 0 | 6.27 | 1 |  |  | 2 | 0.642 | 0 | 58000 |
| 508 | M | 3+7 | 54.25 | Intermediate | 1 | 3.42 | 1 | 1.58 | 1 | 1 | 2.308 | 1 |  |
| 509 | F | 3+7 | 55.47 | Intermediate | 1 | 8.77 | 1 | 5.55 | 1 | 1 | 13.364 | 1 | 49400 |
| 510 | F | 3+7 | 64.25 | Adverse | 0 | 14.58 | 1 |  |  | 2 | 6.212 | 1 | 13390 |
| 511 | F | 3+7 | 25.17 | Intermediate | 0 | 0.36 | 1 |  |  | 2 | 1.205 | 0 | 65200 |
| 512 | F | 3+7 | 61.62 | Intermediate | 0 | 2.76 | 1 |  |  | 2 |  |  | 1300 |
| 513 | M | 3+7 | 20.23 | Favorable | 1 | 3.02 | 1 | 2.04 | 1 | 1 | 15.428 | 1 | 30300 |
| 514 | F | 3+7 | 55.62 | Adverse | 1 | 8.51 | 1 | 7.29 | 1 | 1 | 2.329 | 1 | 22700 |
| 515 | F | 3+7 | 23.52 | Intermediate | 0 | 0.89 | 1 |  |  | 2 | 5.329 | 1 | 10400 |
| 516 | M | 3+7 | 63.54 | Favorable | 0 | 1.15 | 1 |  |  | 2 | 5.799 | 1 | 800 |
| 517 | F | Palliative care | 40.84 | Intermediate | 0 | 1.15 | 1 |  |  | 2 | 4.728 | 1 | 29,000 |
| 518 | F | 3+7 | 56.58 | Intermediate | 1 | 4.04 | 1 | 2.5 | 0 | 2 | 0.119 | 0 | 82500 |
| 519 | F | 3+7 | 37.28 | Favorable | 1 | 6.96 | 0 | 5.94 | 0 | 0 | 0.281 | 0 | 39300 |
| 520 | M | 3+7 | 26.01 | Intermediate | 1 | 9.79 | 1 | 7.55 | 1 | 1 | 1.312 | 0 | 157,000 |
| 521 | F | 3+7 | 43.42 | Adverse | 1 | 10.67 | 1 | 6.67 | 1 | 1 | 14.743 | 1 | 80570 |
| 522 | M | 3+7 | 68.22 | Favorable | 0 | 1.61 | 1 |  |  | 2 |  |  | 46080 |
| 523 | F | 3+7 | 52.63 | Favorable | 0 | 0.79 | 1 |  |  | 2 |  |  | 11400 |
| 524 | F | 3+7 | 42.84 | Favorable | 1 | 8.51 | 1 | 4.2 | 1 | 1 | 1.32 | 0 | 94530 |
| 525 | M | 3+7 | 62.81 | Intermediate | 1 | 12.61 | 0 | 4.7 | 1 | 1 | 3.061 | 1 | 62500 |
| 526 | M | 3+7 | 41.52 | Intermediate | 1 | 12.32 | 0 | 9.26 | 1 | 1 | 2.459 | 1 | 12800 |
| 527 | M | 2+5 | 64.61 | Intermediate | 0 | 1.94 | 1 |  |  | 2 | 11.859 | 1 | 35770 |
| 528 | F | 3+7 | 51.83 | Favorable | 1 | 9.03 | 0 | 7.82 | 0 | 0 | 0.236 | 0 | 1120 |
| 529 | F | Palliative care | 78.68 | Intermediate | 0 | 0.23 | 1 |  |  | 2 | 5.893 | 1 | 217500 |
| 530 | F | 3+7 | 52.54 | Intermediate | 1 | 10.31 | 0 | 7.19 | 0 | 0 | 0.612 | 0 | 73600 |
| 531 | F | 3+7 | 51.66 |  | 1 | 9.13 | 0 | 5.94 | 0 | 0 | 0.276 | 0 | 13000 |
| 532 | M | 3+7 | 40.77 | Intermediate | 1 | 4.93 | 0 | 0.26 | 0 | 0 | 0.568 | 0 |  |
| 533 | F | 3+7 | 51.45 | Intermediate | 1 | 4.37 | 1 | 0.59 | 0 | 2 | 0.559 | 0 | 19920 |
| 534 | M | 3+7 | 39.5 | Favorable | 1 | 5.29 | 0 | 2.59 | 0 | 0 | 0.843 | 0 | 33340 |
